# Supplementary material for: Noncovalent interaction with a spirobipyridine ligand enables efficient iridium-catalyzed C–H activation
Source: Nat Commun. 2024 Apr 17;15:2886. doi: 10.1038/s41467-024-46893-6 (PMC11024094; doi:10.1038/s41467-024-46893-6)
Supplement: Supplementary file 1 — Supplementary Information [file 41467_2024_46893_MOESM1_ESM.pdf]

Supplementary Information

Noncovalent Interaction with a Spirobipyridine Ligand Enables Efficient Iridium-Catalyzed C–H Activation

Yushu Jin, Boobalan Ramadoss, Sobi Asako,* Laurean Ilies*

RIKEN Center for Sustainable Resource Science, 2-1 Hirosawa, Wako, Saitama 351-0198, Japan

sobi.asako@riken.jp, laurean.ilies@riken.jp

Table of contents

I. Supplementary Methods

1. General information S2
2. Synthesis of ligands S3
3. Preparation of starting materials S8
4. Evaluation of various reaction parameters S14
5. Kinetic studies S15
6. Iridium-catalyzed C–H borylation of electron-rich arenes S17
7. Gram-scale synthesis of pharmaceutically relevant compounds S27
8. Mechanistic studies S29
9. DFT studies S35
10. NMR charts S38

II. Supplementary References S83

1. General information

All reactions dealing with air- or moisture-sensitive compounds were carried out in a dry sealed reaction tube under an atmosphere of nitrogen or argon. Air- or moisture-sensitive liquids and solutions were transferred with a syringe or Teflon cannula. Analytical thin-layer chromatography was performed on glass plates coated with 0.25 mm silica gel containing a fluorescent indicator (Merck #1.05715 TLC Silica gel 60 F254). Flash silica gel column chromatography was performed on silica gel 60N (Kanto, spherical and neutral, 40–50 µm) as described by Still et al.[1] ^{1}H NMR and ^{13}C NMR spectra were recorded on a JEOL ECA-500 spectrometers and reported in parts per million referenced to the signals of Me_4Si (^{1}H: 0.00 ppm), $CDCl_3$ (^{13}C: 77.0 ppm). ^{2}H NMR spectra were recorded on a JEOL ECA-500 spectrometers and reported in parts per million referenced to the signal of $CDCl_3$ (7.26 ppm). The data is presented as follows: chemical shift, multiplicity (s = singlet, d = doublet, t = triplet, q = quartet, sept = septet, m = multiplet and/or multiplet resonances, br = broad), coupling constant in Hertz (Hz), and integration. The carbon directly attached to the boron atom was not detected due to quadrupolar broadening. The melting points of solid materials were determined on a Yanaco MP-500 apparatus and were uncorrected. Gas chromatographic (GC) analyses were performed on Shimadzu GC-2025 equipped with an FID detector and a capillary column (HR-1, 0.25-mm i.d. x 25 m). Gel permeation chromatography (GPC) was performed on SHIMADZU LC-20A equipped with FP-2002 columns. Mass spectra (GC-MS) were taken at SHIMADZU QP2010SE equipped with a capillary column (Rxi-5Sil MS, 0.25-mm i.d. x 30 m). High resolution mass spectra were obtained on a Bruker microTOF-Q III (APCI).

Anhydrous tetrahydrofuran, 1,4-dioxane, hexane, and diethyl ether were purchased from KANTO Chemical Co., Inc. and Nacalai Tesque Inc. and purified prior to use by a solvent purification system (GlassContour) equipped with columns of activated alumina and supported copper catalyst.[2] $[Ir(OMe)(cod)]_2$ was purchased from Sigma Aldrich. HBpin was purchased from Acros organics and was used as received. All other reagents were purchased from commercial sources and used as received unless otherwise noted.

2. Synthesis of ligands

Spiro[cyclopenta[2,1-*b*:3,4-*b'*]dipyridine-5,9'-fluorene] [171856-25-0]

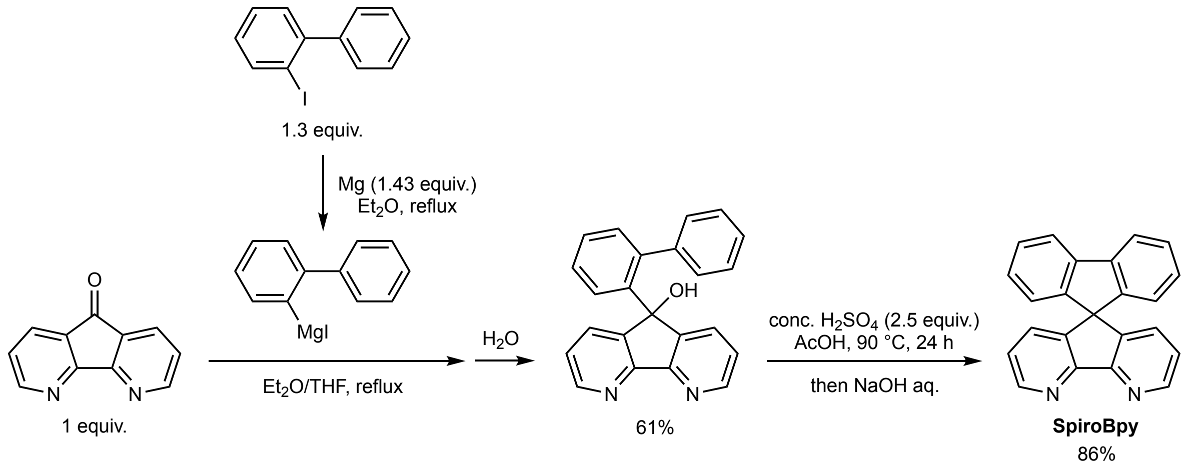

Magnesium turnings (1.1 g, 44 mmol) were placed in a two-neck flask, flamed-dried, and then stirred vigorously for 48 h under a nitrogen atmosphere. To the black powdery magnesium was added Et_2O (10 mL) and subsequently 2-iodo-1,1'-biphenyl (11.2 g, 40 mmol) in Et_2O (50 mL) dropwise over 30 min through a dropping funnel. After complete addition of the aryl iodide solution, the mixture was stirred under reflux (oil bath 50 ˚C) for 1.5 h. After cooling to room temperature, The resulting blackish mixture of [1,1'-biphenyl]-2-ylmagnesium iodide was transferred via cannula to another oven-dried two-neck flask containing 5*H*-cyclopenta[2,1-*b*:3,4-*b'*]dipyridin-5-one[3] (5.6 g, 31 mmol) in dry THF (100 mL) under nitrogen atmosphere. The reaction mixture was then stirred under reflux (oil bath 50 ˚C) for 20 h before quenched by the addition of 100 mL aqueous solution of Rochelle's salt (15 wt%). The mixture was then extracted with $CHCl_3$ three times. The combined organic layer was washed with brine, dried over $MgSO_4$, filtered, and concentrated under reduced pressure. The crude red-brown solid was reprecipitated from hexane/Et_2O/$CHCl_3$ to afford 5-([1,1'-biphenyl]-2-yl)-5*H*-cyclopenta[2,1-*b*:3,4-*b'*]dipyridin-5-ol[3] as an off-white solid (6.3 g, 19 mmol, 61%).

In a 100 mL two-neck flask, 5-([1,1'-biphenyl]-2-yl)-5*H*-cyclopenta[2,1-*b*:3,4-*b*']dipyridin-5-ol (2.6 g, 7.6 mmol) was heated to dissolve in acetic acid (30 mL) under nitrogen atmosphere. Sulfuric acid (1.0 mL, 19 mmol) was then added to the solution, and the mixture was stirred at 90 °C for 24 h. After cooling to room temperature, a large amount of light yellow solid precipitated, and it was collected by filtration and washed with acetic acid and heptane. The solid was neutralized with NaOH (aq.) and extracted with CH_2Cl_2 three times. The combined organic solution was dried over $MgSO_4$, filtered, and concentrated under reduced pressure. The resulting solid was passed through a pad of silica gel (CH_2Cl_2/EtOAc = 1:1) to afford **SpiroBpy**[3] as a colorless solid (2.1 g, 6.6 mmol, 86%). It can be further purified by recrystallization from $CHCl_3$/Et_2O/pentane to afford a colorless crystalline solid.

^{1}H NMR (500 MHz, $CDCl_3$): δ 8.74 (dd, J = 3.5, 2.8 Hz, 2H), 7.87 (d, J = 7.4 Hz, 2H), 7.41 (dd, J = 8.3, 7.2 Hz, 2H), 7.16–7.12 (m, 6H), 6.73 (d, J = 7.4 Hz, 2H).

^{13}C NMR (125 MHz, $CDCl_3$): δ 158.9, 150.3, 146.0, 143.5, 141.8, 131.6, 128.3, 128.0, 123.7, 123.5, 120.3, 61.5.

2',7'-Dibromospiro[cyclopenta[2,1-*b*:3,4-*b'*]dipyridine-5,9'-fluorene] [198142-63-1]

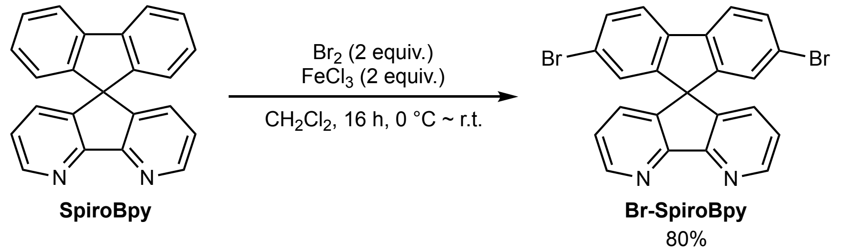

To an oven-dried 100 mL Schlenk flask was added **SpiroBpy** (1.6 g, 5.0 mmol) and dry iron(III) chloride (1.6 g, 10 mmol) in dry CH_2Cl_2 (20 mL) under N_2 atmosphere. The mixture was cooled to 0 °C, and bromine (0.5 mL, 10 mmol) was added slowly. The mixture was warmed to room temperature and stirred for 16 h. The reaction was quenched with saturated $NaHCO_3$ aq. and extracted with CH_2Cl_2 three times. The combined organic layer was dried over Na_2SO_4, filtered, and concentrated under reduced pressure. The crude residue was purified by silica gel column chromatography (eluent: hexane/EtOAc = 1:1) to afford **Br-SpiroBpy**[3] as an off-white solid (1.9 g, 4.0 mmol, 80%).

^{1}H NMR (500 MHz, $CDCl_3$): δ 8.79 (dd, J = 4.9, 1.4 Hz, 2H), 7.70 (d, J = 8.0 Hz, 2H), 7.55 (dd, J = 8.0, 1.7 Hz, 2H), 7.18 (dd, J = 8.0, 4.6 Hz, 2H), 7.13 (dd, J = 7.4, 1.7 Hz, 2H), 6.85 (d, J = 1.7 Hz, 2H).

^{13}C NMR (125 MHz, $CDCl_3$): δ 158.9, 150.9, 147.9, 142.1, 139.7, 131.9, 131.8, 127.2, 123.9, 122.2, 121.8, 61.2.

2',7'-Bis(4,4,5,5-tetramethyl-1,3,2-dioxaborolan-2-yl)spiro[cyclopenta[2,1-*b*:3,4-*b'*]dipyridine-5,9'-fluorene] [2763205-15-6]

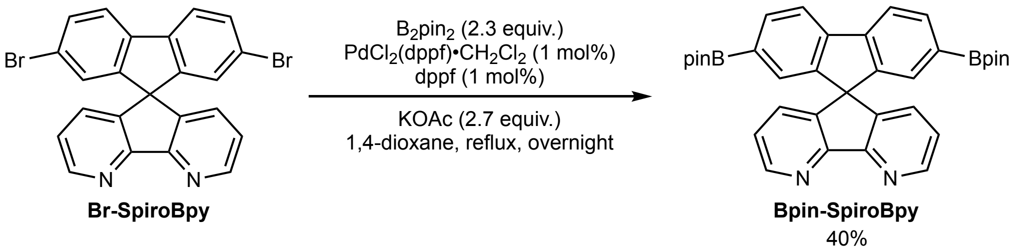

To an oven-dried 200 mL Schlenk flask equipped with a reflux condenser and a septum was added **Br-SpiroBpy** (500 mg, 1.1 mmol), KOAc (295 mg, 3 mmol), bis(pinacolato)diboron (670 mg, 2.5 mmol), $[PdCl_2(dppf)]$•CH_2Cl_2 (86 mg, 10 μmol), and 1,1'-bis(diphenylphosphino)ferrocene (52 mg, 10 μmol) under argon atmosphere. Anhydrous 1,4-dioxane (100 mL) was added and the mixture was stirred under reflux (oil bath temperature: 110 °C) overnight. After cooling to room temperature, the reaction was quenched by the addition of water, and then extracted with CH_2Cl_2 several times. The combined organic layer was washed with brine, dried over $MgSO_4$, filtered, and concentrated under reduced pressure. The residue was purified by silica gel column chromatography (eluent: EtOAc) to afford **Bpin-SpiroBpy**[3] as an off-white solid (240 mg, 0.42 mmol, 40%). It could be further purified by passing through a short Florisil pad (eluent: $CHCl_3$), followed by recrystallization ($Et_2O/CHCl_3$) to obtain **Bpin-SpiroBpy** as colorless plates.

^{1}H NMR (500 MHz, $CDCl_3$): δ 8.74 (dd, *J* = 4.6, 1.1 Hz, 2H), 7.90–7.87 (m, 4H), 7.16 (s, 2H), 7.13–7.07 (m, 4H), 1.25 (s, 24H).
^{13}C NMR (125 MHz, $CDCl_3$): δ 159.2, 150.4, 146.0, 144.5, 143.5, 135.1, 131.9, 130.0, 123.7, 120.1, 83.9, 61.5, 24.7.

2',7'-Diphenylspiro[cyclopenta[2,1-*b*:3,4-*b'*]dipyridine-5,9'-fluorene] [2763205-06-5]

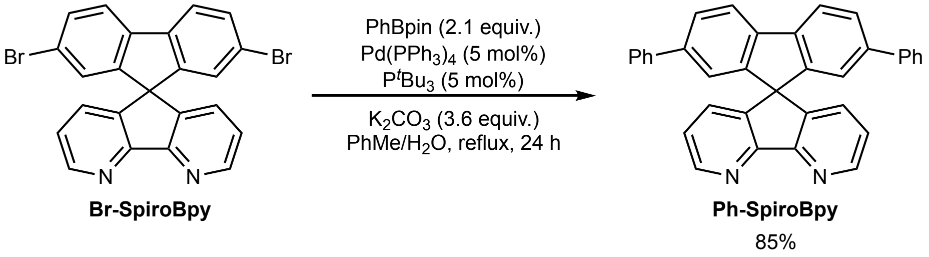

To an oven-dried 50 mL Schlenk tube was added **Br-SpiroBpy** (500 mg, 1.1 mmol), phenylboronic acid pinacol ester (500 mg, 2.4 mmol), and $Pd(PPh_3)_4$ (60.6 mg, 52 µmol) in toluene (20 mL) under N_2 atmosphere. 2 M K_2CO_3 aq. (2.0 mL, 4.0 mmol) and tri-*tert*-butylphosphine (0.1 M in toluene, 0.5 mL, 50 µmol) were added via syringe, and the reaction was stirred under reflux (oil bath temperature: 130 ºC) for 24 h. After cooling to room temperature, water was added, and the mixture was extracted with $CHCl_3$ several times. The combined organic layer was dried over $MgSO_4$, filtered, and concentrated under reduced pressure. The crude residue was passed through a short silica gel pad, and then recrystallized from $CHCl_3$/EtOAc/hexane = 2/1/2 solution to afford **Ph-SpiroBpy**[3] as a pale-yellow solid (420 mg, 0.89 mmol, 85%).
^{1}H NMR (500 MHz, $CDCl_3$): δ 8.74 (dd, *J* = 4.9, 1.4 Hz, 2H), 7.93 (d, *J* = 8.0 Hz, 2H), 7.67 (dd, *J* = 1.5, 8.0 Hz, 2H), 7.40 (d, *J* = 6.9 Hz, 4H), 7.32 (t, *J* = 7.4 Hz, 4H), 7.26–7.23 (m, 2H), 7.20 (dd, *J* = 7.7, 1.4 Hz, 2H), 7.12 (dd, *J* = 7.5, 4.6 Hz, 2H), 6.94 (d, *J* = 1.1 Hz, 2H).
^{13}C NMR (125 MHz, $CDCl_3$): δ 159.0, 150.5, 147.2, 143.6, 141.3, 140.7, 140.3, 132.0, 128.7, 127.51, 127.46, 126.9, 123.8, 122.4, 120.7, 61.7.

2',7'-Di-*tert*-butylspiro[cyclopenta[2,1-*b*:3,4-*b'*]dipyridine-5,9'-fluorene]

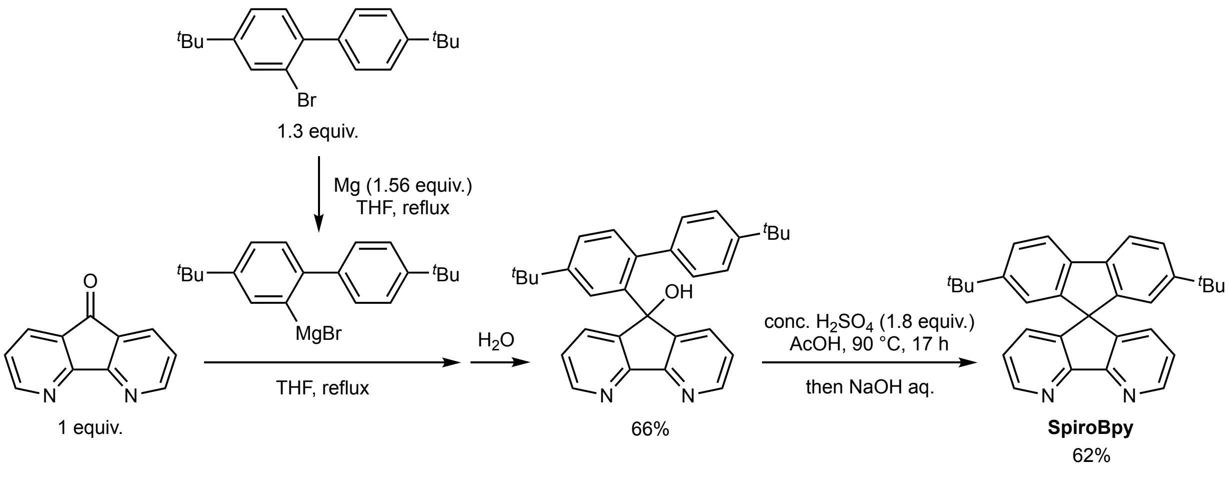

Magnesium turnings (600 mg, 24.7 mmol) were placed in a two-neck flask, flamed-dried, and then stirred vigorously for 48 h under a nitrogen atmosphere. To the black powdery magnesium was added THF (5 mL) and subsequently 2-bromo-4,4'-di-*tert*-butylbiphenyl[4] (7.0 g, 20.2 mmol) in THF (45 mL) dropwise over 30 min through a dropping funnel. The reaction vessel was occasionally heated to reflux at the beginning of the dropwise addition to accelerate the generation of the Grignard reagent. After complete addition of the aryl bromide solution, the mixture was stirred under reflux for 30 min. To the resulting black mixture of (4,4'-di-*tert*-butyl-[1,1'-biphenyl]-2-yl)magnesium bromide was added 5*H*-cyclopenta[2,1-*b*:3,4-*b'*]dipyridin-5-one[3] (2.8 g, 15.4 mmol) in one portion at room temperature. The reaction mixture was then stirred under reflux for 19 h before quenched by the addition of 100 mL aqueous solution of Rochelle's salt (15 wt%). The mixture was then extracted with EtOAc (70 mL) three times. The combined organic layer was washed with brine (50 mL), dried over Na_2SO_4, filtered, and concentrated under reduced pressure. The crude red-brown solid was reprecipitated from hexane/CH_2Cl_2 to afford the target compound 5-(4,4'-di-*tert*-butyl-[1,1'-biphenyl]-2-yl)-5*H*-cyclopenta[2,1-*b*:3,4-*b'*]dipyridin-5-ol (4.56 g, 10.2 mmol, 66%) as an off-white solid.
^{1}H NMR (500 MHz, $CDCl_3$) δ 8.50 (d, J = 2.3 Hz, 1H), 8.39 (d, J = 5.2 Hz, 2H), 7.52 (dd, J = 7.4, 1.7 Hz, 2H), 7.36 (dd, J = 8.0, 2.3 Hz, 1H), 7.04 (dd, J = 8.0, 5.0 Hz, 2H), 6.92 (d, J = 8.0 Hz, 1H), 6.66 (d, J = 8.0 Hz, 2H), 5.94 (d, J = 8.0 Hz, 2H), 2.92 (s, 1H), 1.49 (s, 9H), 1.20 (s, 9H).

To a suspension of 5-(4,4'-di-*tert*-butyl-[1,1'-biphenyl]-2-yl)-5*H*-cyclopenta[2,1-*b*:3,4-*b'*]dipyridin-5-ol (4.55 g, 10.1 mmol) in AcOH (30 mL) was added conc. H_2SO_4 (1.0 mL, 18.8 mmol) slowly to form a clear red solution. The solution was stirred at 90 °C for 17 h, and then cooled to room temperature to form a precipitate. The precipitates were collected by filtration, washed with AcOH and hexane, dried under air to give 5.84 g of the corresponding pyridinium sulfate salt as a yellow-orange solid. The salt was then dissolved in 1*N* aqueous NaOH (75 mL), and extracted with CH_2Cl_2 (50 mL) three times. The combined organic layer was washed with 1*N* aqueous NaOH (20 mL) and brine (20 mL), dried over Na_2SO_4, filtered, and concentrated under reduced pressure. The resulting solid was triturated in MeCN with sonication, filtered, washed with MeCN, and dried under vacuum to afford the target compound ***ᵗ*Bu-SpiroBpy** (2.68 g, 6.21 mmol, 62% yield) as a pale-yellow solid.
^{1}H NMR (500 MHz, $CDCl_3$): δ 8.76–8.74 (m, 2H), 7.73 (d, J = 8.0 Hz, 2H), 7.42 (dd, J = 8.0, 1.7 Hz, 2H), 7.13–7.12 (m, 4H), 6.67 (d, J = 1.7 Hz, 2H), 1.15 (s, 18H).
^{13}C NMR (125 MHz, $CDCl_3$): δ 159.0, 151.3, 150.2, 146.3, 144.4, 139.3, 131.9, 125.5, 123.7, 120.4, 119.4, 61.8, 34.9, 31.4.
HRMS (APCI) *m*/*z*: $[M + H]^+$ Calcd for $C_{31}H_{31}N_2$ 431.2482; Found 431.2481.
Melting point: 273–274 °C.

5*H*-Cyclopenta[2,1-*b*:3,4-*b'*]dipyridine (L1) [245-37-4]

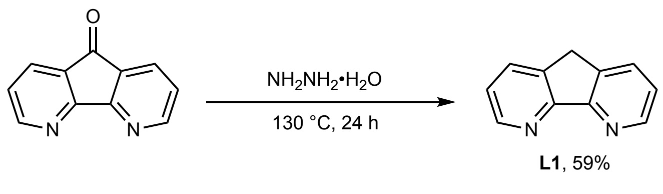

To a 50 mL Schlenk flask was added 5*H*-cyclopenta[2,1-*b*:3,4-*b'*]dipyridin-5-one (1.0 g, 5.5 mmol) and $NH_2NH_2 \cdot H_2O$ (5.0 mL). The reaction was stirred at 130 °C for 24 h. After cooling to room

temperature, the mixture was extracted with CH_2Cl_2 three times. The combined organic layer was dried over $MgSO_4$, filtered, and concentrated under reduced pressure. The crude product was purified by silica gel column chromatography (eluent: EtOAc) to afford the target compound **L1**[3] as a colorless solid (540 mg, 3.2 mmol, 59%).
^{1}H NMR (500 MHz, $CDCl_3$): δ 8.75 (d, J = 4.6 Hz, 2H), 7.89 (d, J = 7.4 Hz, 2H), 7.31 (dd, J = 7.7, 4.9 Hz, 2H), 3.89 (s, 2H).
^{13}C NMR (125 MHz, $CDCl_3$): δ 158.7, 149.2, 137.1, 132.6, 122.4, 32.0.

5,5-Diphenyl-5*H*-cyclopenta[2,1-*b*:3,4-*b′*]dipyridine (L2) [1664365-55-2]

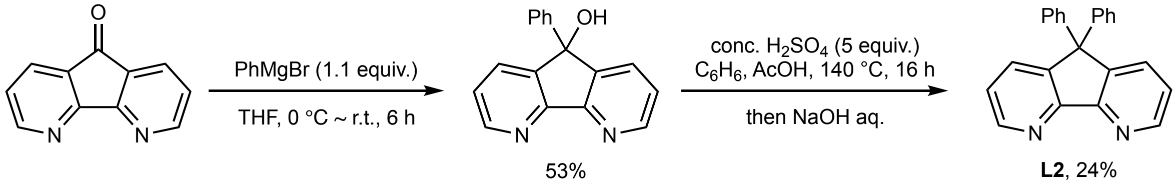

To an oven-dried 50 mL Schlenk flask was added 5*H*-cyclopenta[2,1-*b*:3,4-*b′*]dipyridin-5-one (182 mg, 1.0 mmol) under N_2 atmosphere. Dry THF (5 mL) was charged via syringe, and the mixture was cooled to 0 °C. Phenylmagnesium bromide (1.0 M in THF, 1.1 mL, 1.1 mmol) was slowly added to the mixture via syringe. After addition, the mixture was gradually warmed to room temperature and stirred for 6 h. The reaction was quenched by adding water, and the mixture was extracted with $CHCl_3$ three times. The combined organic layer was dried over $MgSO_4$, filtered, and concentrated under reduced pressure. The crude residue was purified by silica gel column chromatography (eluent: hexane/EtOAc = 1:2, then EtOAc) to afford 5-phenyl-5*H*-cyclopenta[2,1-*b*:3,4-*b′*]dipyridin-5-ol[5] as a colorless solid (137 mg, 0.53 mmol, 53%).
^{1}H NMR (500 MHz, $CDCl_3$): δ 8.59 (d, J = 5.2 Hz, 2H), 7.68 (dd, J = 7.7, 1.4 Hz, 2H), 7.37 (dd, J = 7.7, 2.0 Hz, 2H), 7.31–7.26 (m, 3H), 7.21–7.18 (m, 2H), 3.38 (br, 1H).

In a screw cap test tube, to a mixture of 5-phenyl-5*H*-cyclopenta[2,1-*b*:3,4-*b′*]dipyridin-5-ol (52 mg, 0.2 mmol) in AcOH (0.5 mL) and benzene (0.5 mL) was added conc. H_2SO_4 (50 µL, 1 mmol) under N_2 atmosphere. The tube was tightly sealed and stirred at 140 °C for 16 h. After cooling to room temperature, the mixture was poured into crushed ice with NaOH, and then extracted with $CHCl_3$ three times. The combined organic layer was dried over $MgSO_4$, filtered, and concentrated under reduced pressure. The crude residue was purified by silica gel column chromatography (eluent: EtOAc) to afford the target compound **L2** as a colorless solid (15.2 mg, 47 µmol, 24%). The spectral data of **L2** was in good agreement with the literature.[5]
^{1}H NMR (500 MHz, $CDCl_3$): δ 8.74 (dd, J = 4.9, 1.4 Hz, 2H), 7.77 (dd, J = 8.0, 1.1 Hz, 2H), 7.29–7.24 (m, 8H), 7.18–7.16 (m, 4H).
^{13}C NMR (125 MHz, $CDCl_3$): δ 157.6, 150.2, 145.6, 143.7, 133.7, 128.6, 127.8, 127.4, 123.5, 61.6.

3. Preparation of starting materials

Preparation of substrates **1e**, **1f**, **1k**, **1m**, **1n**, **1o**, **1p**, **1q**, **1v**, **1w**, **3b**, and **3c** is described below. Other substrates were purchased from commercial sources and were used as received.

Triisopropyl(*m*-tolyloxy)silane (1e) [186374-69-6]

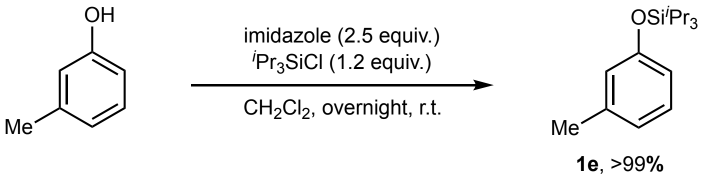

To an oven-dried 50 mL Schlenk flask was added imidazole (340 mg, 5.0 mmol) under N_2 atmosphere. Subsequently, dry CH_2Cl_2 (8 mL), *m*-cresol (216 mg, 2.0 mmol), and triisopropylsilyl chloride (0.5 mL, 2.4 mmol) were added via syringe. The mixture was stirred at room temperature overnight. The mixture was added H_2O, and then extracted with CH_2Cl_2 three times. The combined organic layer was dried over Na_2SO_4, filtered, and concentrated under reduced pressure. The crude residue was purified by silica gel column chromatography (eluent: hexane) to afford the target compound **1e** as colorless oil (525 mg, 2.0 mmol, >99%). The spectral data of product **1e** was in good agreement with the literature.[6]

^{1}H NMR (500 MHz, $CDCl_3$): δ 7.08 (t, *J* = 7.7 Hz, 1H), 6.74 (d, *J* = 7.4 Hz, 1H), 6.70 (s, 1H), 6.67 (dd, *J* = 8.0, 2.3 Hz, 1H), 2.29 (s, 3H), 1.25 (sept, *J* = 7.4 Hz, 3H), 1.10 (d, *J* = 7.4 Hz, 18H).

^{13}C NMR (125 MHz, $CDCl_3$): δ 155.9, 139.3, 129.0, 121.7, 120.7, 116.7, 21.4, 17.9, 12.7.

4,4''-Di-*tert*-butyl-1,1':3',1''-terphenyl (1f) [164155-50-4]

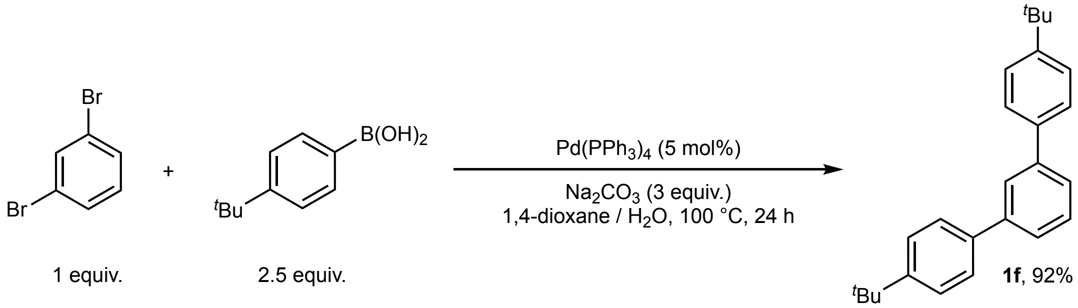

To an oven-dried 100 mL Schlenk flask was added $Pd(PPh_3)_4$ (289 mg, 0.25 mmol), (4-(*tert*-butyl)phenyl)boronic acid (2.23 g, 12.5 mmol), and Na_2CO_3 (1.59 g, 15 mmol) under N_2 atmosphere. Subsequently, 1,4-dioxane (20 mL), H_2O (5 mL), and 1,3-dibromobenzene (1.18 g, 5.0 mmol) were added via syringe. The mixture was stirred at 100 °C for 24 h. After cooling to room temperature, the mixture was diluted with EtOAc, and then extracted with EtOAc three times. The combined organic layer was dried over $MgSO_4$, filtered, and concentrated under reduced pressure. The crude residue was purified by silica gel column chromatography (eluent: hexane) to afford the target compound **1f** as a colorless solid (1.58 g, 4.6 mmol, 92%). The spectral data of product **1f** was in good agreement with the literature.[7]

^{1}H NMR (500 MHz, $CDCl_3$): δ 7.80 (t, *J* = 1.7 Hz, 1H), 7.60–7.58 (m, 4H), 7.56–7.54 (m, 2H), 7.49–7.47 (m, 5H), 1.38 (s, 18H).

^{13}C NMR (125 MHz, $CDCl_3$): δ 150.4, 141.5, 138.3, 129.0, 126.9, 125.9, 125.7, 34.5, 31.4.

1,3-Bis(trimethylsilyl)benzene (1k) [2060-89-1]

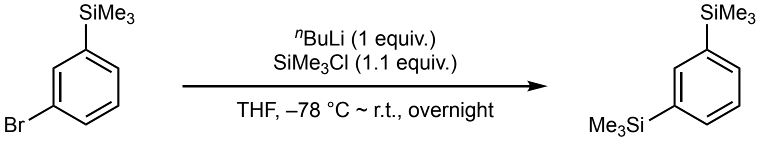

1k, 57%

To an oven-dried 50 mL Schlenk flask was added (3-bromophenyl)trimethylsilane (688 mg, 3.0 mmol) in dry THF (15 mL) under N_2 atmosphere. The solution was cooled to –78 °C, and then ^{n}BuLi (1.57 M in hexane, 1.9 mL, 3.0 mmol) was added dropwise via syringe. The mixture was stirred at –78 °C for 1 h. Subsequently, chlorotrimethylsilane (360 mg, 3.3 mmol) was added dropwise to the mixture via syringe at –78 °C. The mixture was gradually warmed up to room temperature and stirred for overnight. The reaction was quenched by adding water, and then extracted with CH_2Cl_2 two times. The combined organic layer was dried over Na_2SO_4, filtered, and concentrated under reduced pressure. The crude residue was passed through a short silica gel plug (eluent: hexane/EtOAc = 10/1) to afford the target compound **1k** as colorless oil (378 mg, 1.7 mmol, 57%). The spectral data of product **1k** was in good agreement with the literature.[8]
^{1}H NMR (500 MHz, $CDCl_3$): δ 7.67 (s, 1H), 7.52 (dd, J = 7.2, 1.4 Hz, 2H), 7.34 (t, J = 7.2 Hz, 1H), 0.27 (s, 18H).
^{13}C NMR (125 MHz, $CDCl_3$): δ 139.5, 138.2, 133.9, 127.1, –1.0.

***N*^{1},*N*^{1},*N*^{3},*N*^{3}-Tetraethylbenzene-1,3-diamine (1m) [64287-26-9] & 3-chloro-*N*,*N*-diethylaniline (1v) [6375-75-3]**

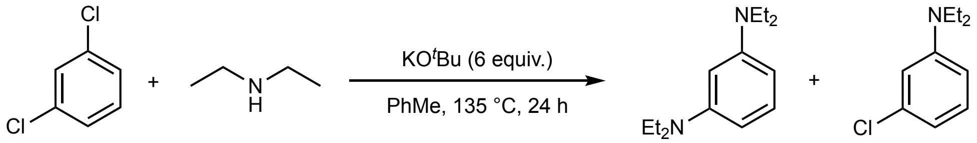

1 equiv. 3 equiv. **1m**, 36% **1v**, 23%

The preparation was according to a slightly modified literature procedure.[9] To an oven-dried 100 mL Schlenk flask was added potassium *tert*-butoxide (2.7 g, 24 mmol) under N_2 atmosphere. Toluene (10 mL), 1,3-dichlorobenzene (588 mg, 4.0 mmol), and diethylamine (878 mg, 12 mmol) were added via syringe. The mixture was stirred vigorously at 135 °C under reflux for 24 h. After cooling to room temperature, to the mixture was added water and then it was extracted with CH_2Cl_2 three times. The combined organic layer was dried over Na_2SO_4, filtered, and concentrated under reduced pressure. The crude residue was purified by silica gel column chromatography (eluent: hexane/EtOAc = 50/1 + 0.5% Et_3N, then hexane/EtOAc = 20/1 + 0.5% Et_3N) to afford the target compound **1v** as colorless oil (172 mg, 0.94 mmol, 23%), and a mixture containing **1m** and impurities. The mixture was further purified by PTLC (eluent: hexane/EtOAc = 10/1 + 0.5% Et_3N) to afford the target compound **1m** as colorless oil (317 mg, 1.44 mmol, 36%).

Compound **1m**. The spectral data of product **1m** was in good agreement with the literature.[10]
^{1}H NMR (500 MHz, $CDCl_3$): δ 7.05 (t, J = 8.0 Hz, 1H), 6.07 (dd, J = 8.3, 2.6 Hz, 2H), 6.00 (t, J = 2.6 Hz, 1H), 3.33 (q, J = 7.1 Hz, 8H), 1.16 (t, J = 6.9 Hz, 12H).

^{13}C NMR (125 MHz, $CDCl_3$): δ 149.0, 129.8, 100.7, 96.1, 44.5, 12.8.

Compound **1v**. The spectral data of product **1v** was in good agreement with the literature.[11]
^{1}H NMR (500 MHz, $CDCl_3$): δ 7.09 (t, J = 8.3 Hz, 1H), 6.62–6.58 (m, 2H), 6.52 (dd, J = 8.6, 3.4 Hz, 1H), 3.33 (q, J = 7.1 Hz, 4H), 1.15 (t, J = 6.9 Hz, 6H).
^{13}C NMR (125 MHz, $CDCl_3$): δ 148.8, 135.2, 130.1, 115.0, 111.4, 109.7, 44.3, 12.4.

1,3-Di(piperidin-1-yl)benzene (1n) [27594-19-0] & 1-(3-chlorophenyl)piperidine (1w) [27594-19-0]

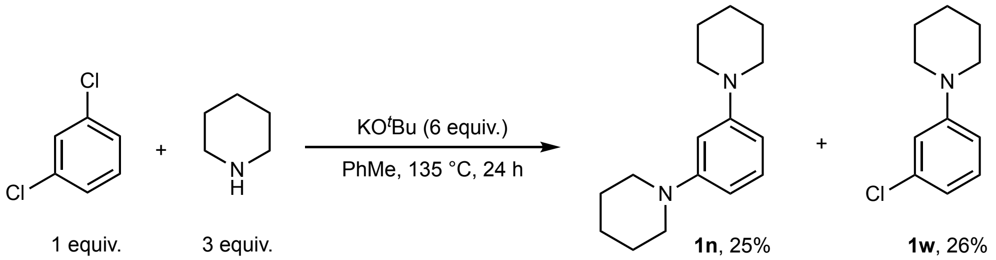

The preparation was according to a slightly modified literature procedure.[9] To an oven-dried 100 mL Schlenk flask was added potassium *tert*-butoxide (2.7 g, 24 mmol) under N_2 atmosphere. Toluene (10 mL), 1,3-dichlorobenzene (588 mg, 4.0 mmol), and piperidine (1.0 g, 12 mmol) were added via syringe. The mixture was stirred vigorously at 135 °C under reflux for 24 h. After cooling to room temperature, to the mixture was added water and then it was extracted with CH_2Cl_2 three times. The combined organic layer was dried over Na_2SO_4, filtered, and concentrated under reduced pressure. The crude residue was purified by silica gel column chromatography (eluent: hexane/EtOAc = 20/1) to afford the target compound **1n** as a pale-yellow oil (243 mg, 1.0 mmol, 25%), and a mixture containing **1w** and impurities. The mixture was further purified by PTLC (eluent: hexane) to afford the target compound **1w** a pale-yellow oil (203 mg, 1.0 mmol, 26%).

Compound **1n**. The spectral data of product **1n** was in good agreement with the literature.[9]
^{1}H NMR (500 MHz, $CDCl_3$): δ 7.12 (t, J = 8.0 Hz, 1H), 6.54 (t, J = 2.3 Hz, 1H), 6.46 (dd, J = 8.0, 2.3 Hz, 2H), 3.14–3.11 (m, 8H), 1.72–1.68 (m, 8H), 1.58–1.55 (m, 4H).
^{13}C NMR (125 MHz, $CDCl_3$): δ 153.3, 129.2, 108.5, 105.9, 51.1, 26.0, 24.4.

Compound **1w**. The spectral data of product **1w** was in good agreement with the literature.[12]
^{1}H NMR (500 MHz, $CDCl_3$): δ 7.13 (t, J = 8.0 Hz, 1H), 6.87 (t, J = 2.3 Hz, 1H), 6.79 (dd, J = 8.0, 2.3 Hz, 1H), 6.76–6.74 (m, 1H), 3.17–3.15 (m, 4H), 1.71–1.66 (m, 4H), 1.60–1.57 (m, 4H).
^{13}C NMR (125 MHz, $CDCl_3$): δ 153.1, 134.8, 129.9, 118.6, 116.0, 114.2, 50.1, 25.6, 24.2.

***N*^{1},*N*^{1},*N*^{2},*N*^{2}-Tetramethylbenzene-1,2-diamine (1o) [704-01-8]**

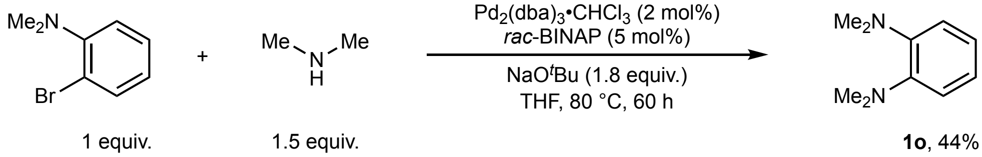

The preparation was according to a slightly modified literature procedure.[13] In an argon-filled glove box, $Pd_2(dba)_3$•$CHCl_3$ (41.4 mg, 40 μmol), racemic BINAP (62.3 mg, 0.10 mmol), and NaO^{t}Bu (346 mg, 3.6 mmol) were placed in a J-Young Schlenk tube. After removal from the glove box, 2-bromo-*N*,*N*-dimethylaniline (400 mg, 2.0 mmol) and dimethylamine (2.0 M in THF, 1.5 mL, 3.0 mmol) were charged to the tube via syringe. The tube was sealed tightly, and the reaction mixture was stirred at 80 °C for 60 h. After cooling to room temperature, to the mixture was added water and then it was extracted with EtOAc three times. The combined organic layer was dried over $MgSO_4$, filtered, and concentrated under reduced pressure. The crude residue was purified by silica gel column chromatography (eluent: hexane/EtOAc = 30/1) to afford the target compound **1o** as colorless oil (144 mg, 0.88 mmol, 44%). The spectral data of product **1o** was in good agreement with the literature.[14]
^{1}H NMR (500 MHz, $CDCl_3$): δ 6.93–6.88 (m, 4H), 2.79 (s, 12H).
^{13}C NMR (125 MHz, $CDCl_3$): δ 145.2, 121.6, 117.8, 41.4.

1,4-Dimethyl-1,2,3,4-tetrahydroquinoxaline (1p) [2427-06-7]

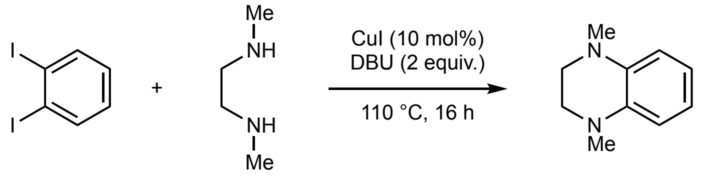

To an oven-dried 50 mL Schlenk flask was added copper(I) iodide (38 mg, 0.20 mmol) under N_2 atmosphere. Subsequently, 1,2-diiodobenzene (660 mg, 2.0 mmol), 1,8-diazabicyclo[5.4.0]-7-undecene (609 mg, 4.0 mmol), and N^1,N^2-dimethylethane-1,2-diamine (0.43 mL, 4.0 mmol) were added via syringe. The mixture was stirred at 110 °C for 16 h. After cooling to room temperature, the mixture was directly subjected to silica gel column chromatography (eluent: hexane/EtOAc = 10:1) to afford the target compound **1p** as a pale-yellow oil (170 mg, 1.0 mmol, 52%). The spectral data of product **1p** was in good agreement with the literature.[15]
^{1}H NMR (500 MHz, $CDCl_3$): δ 6.69–6.66 (m, 2H), 6.55–6.52 (m, 2H), 3.33 (s, 4H), 2.86 (s, 6H).
^{13}C NMR (125 MHz, $CDCl_3$): δ 136.7, 118.2, 110.7, 49.9, 39.2.

3,6-Di-*tert*-butyl-9-(3-methoxyphenyl)-9*H*-carbazole (1q) [1567716-30-6]

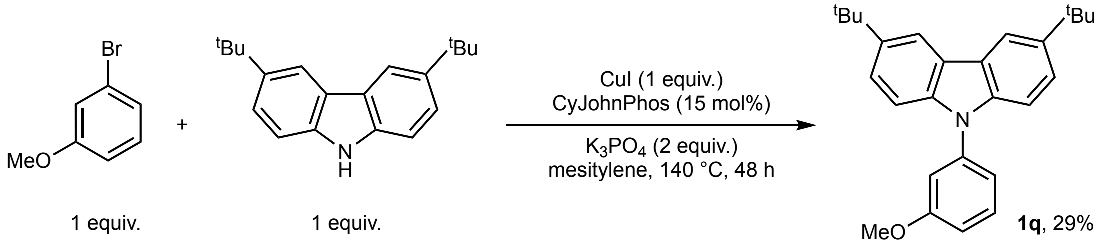

The preparation was according to a slightly modified literature procedure.[16] To an oven-dried 100 mL Schlenk flask was added 3,6-di-*tert*-butyl-9*H*-carbazole (671 mg, 2.4 mmol), copper(I) iodide (457 mg, 2.4 mmol), CyJohnPhos (126 mg, 0.36 mmol), and K_3PO_4 (1.0 g, 4.8 mmol) under N_2

atmosphere. Subsequently, dry mesitylene (10 mL) and 3-bromoanisole (450 mg, 2.4 mmol) were added via syringe. The mixture was stirred at 140 °C for 48 h. After cooling to room temperature, the mixture was added water and then extracted with EtOAc three times. The combined organic layer was dried over $MgSO_4$, filtered, and concentrated under reduced pressure. The crude residue was purified by silica gel column chromatography (eluent: hexane, then hexane/EtOAc = 40/1) to afford the target compound **1q** as a colorless solid (264 mg, 0.68 mmol, 29%). The spectral data (^{1}H NMR only) of product **1q** was in good agreement with the literature.[17]

^{1}H NMR (500 MHz, $CDCl_3$): δ 8.13 (d, *J* = 1.8 Hz, 2H), 7.47 (t, *J* = 8.0 Hz, 1H), 7.46 (dd, *J* = 8.6, 1.8 Hz, 2H), 7.38 (d, *J* = 8.6 Hz, 2H), 7.16–7.14 (m, 1H), 7.10 (dd, *J* = 2.3, 2.3 Hz, 1H), 6.99–6.96 (m, 1H), 3.85 (s, 3H), 1.46 (s, 18H).

^{13}C NMR (125 MHz, $CDCl_3$): δ 160.7, 142.8, 139.3, 139.1, 130.4, 123.6, 123.3, 118.9, 116.2, 112.9, 112.1, 109.3, 55.5, 34.7, 32.0.

HRMS (APCI) *m/z*: $[M + H]^+$ Calcd for $C_{27}H_{32}NO$ 386.2478; Found 386.2472.

Melting point: 152–153 °C.

Methyl 2-((*tert*-butoxycarbonyl)amino)-3-(3-methoxyphenyl)propanoate (3b) [190905-50-1]

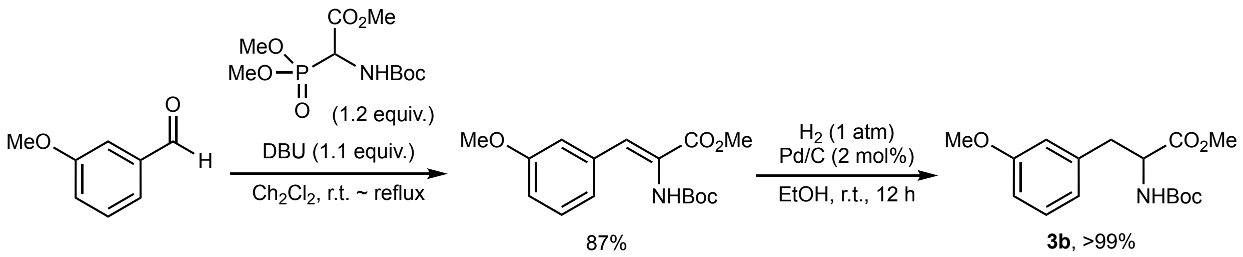

To a solution of *N*-(*tert*-butoxycarbonyl)-2-phosphonoglycine trimethyl ester (1.8 g, 6.1 mmol) in dry CH_2Cl_2 (10 mL) was added 1,8-diazabicyclo[5.4.0]-7-undecene (0.82 mL, 5.5 mmol). After stirring at room temperature for 10 min, *m*-anisaldehyde (0.61 mL, 5.0 mmol) was slowly added via syringe. The mixture was stirred at room temperature for 2.5 h and then reflux for 1 h. After cooling to room temperature, all volatiles were removed under vacuum. The residue was added 1N HCl aq. and extracted with EtOAc three times. The combined organic layer was dried over $MgSO_4$, filtered, and concentrated under reduced pressure. The crude residue was purified by silica gel column chromatography (eluent: hexane/EtOAc = 10:1 then 3:1) to afford methyl 2-((*tert*-butoxycarbonyl)amino)-3-(3-methoxyphenyl)acrylate[18] as a colorless solid (1.33 g, 4.3 mmol, 87%).

^{1}H NMR (500 MHz, $CDCl_3$): δ 7.28 (t, *J* = 7.4 Hz, 1H), 7.21 (s, 1H), 7.12 (d, *J* = 7.4 Hz, 1H), 7.09 (t, *J* = 2.0 Hz, 1H), 6.16 (br, 1H), 3.86 (s, 3H), 3.81 (s, 3H), 1.40 (s, 9H).

To a 100 mL Schlenk flask was added methyl 2-((*tert*-butoxycarbonyl)amino)-3-(3-methoxy phenyl)acrylate (1.23 g, 4.0 mmol) and Pd/C (10 wt% Pd, 5 wt% H_2O, 90 mg, 80 μmol) under H_2 atmosphere. Subsequently, EtOH (10 mL) was added via syringe. The reaction was stirred at room temperature for 12 h. The mixture was carefully filtered through a celite pad and washed with EtOAc. Removal of all volatiles under reduced pressure afforded the target compound **3b** as a colorless solid (1.26 g, 4.0 mmol, >99%). The spectral data of product **3b** was in good agreement with the literature.[18]

^{1}H NMR (500 MHz, $CDCl_3$): δ 7.21 (t, J = 7.7 Hz, 1H), 6.79 (dd, J = 8.0, 2.3 Hz, 1H), 6.71 (d, J = 7.4 Hz, 1H), 6.67 (s, 1H), 4.97 (d, J = 8.0 Hz, 1H), 4.58 (dd, J = 13.7, 5.7 Hz, 1H), 3.79 (s, 3H), 3.72 (s, 3H), 3.09 (dd, J = 13.7, 5.7 Hz, 2H), 3.03 (dd, J = 13.7, 5.7 Hz, 2H), 1.42 (s, 9H).
^{13}C NMR (125 MHz, $CDCl_3$): δ 172.3, 159.6, 155.1, 137.5, 129.5, 121.6, 114.9, 112.4, 79.9, 55.1, 54.3, 52.2, 38.3, 28.3.

Ethyl 4-methoxy-1-(triisopropylsilyl)-1*H*-indole-3-carboxylate (3c) [2210231-60-8]

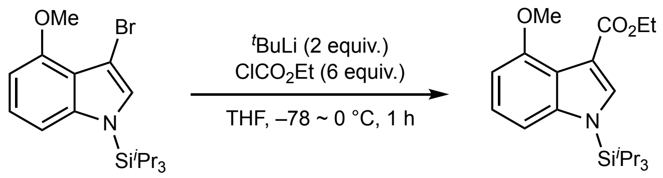

3c, 81%

To an oven-dried 100 mL Schlenk flask was added 3-bromo-4-methoxy-1-(triisopropylsilyl)-1*H*-indole[19] (1.9 g, 5.0 mmol) under N_2 atmosphere. Dry THF (10 mL) was added via syringe, and the solution was cooled to –78 °C. ^{t}BuLi (1.62 M in pentane, 6.2 mL, 10 mmol) was slowly added to the solution via syringe to give a yellowish solution. The solution was stirred at –78 °C for 15 min, and then ethyl chloroformate (2.85 mL, 30 mmol) was added dropwise at –78 °C. The mixture was stirred at –78 °C for another 10 min, and then gradually warmed to room temperature. The mixture was stirred at room temperature for 1 h, and quenched by adding sat. NH_4Cl aq. The mixture was then extracted with EtOAc several times, washed with brine. The combined organic layer was dried over $MgSO_4$, filtered, and concentrated under reduced pressure. The crude residue was purified by silica gel column chromatography (eluent: hexane/EtOAc = 20:1 then 10:1) to afford the target compound 3c as an off-white solid (1.5 g, 4.0 mmol, 81%). The spectral data of product **3c** was in good agreement with the literature.[20]
^{1}H NMR (500 MHz, $CDCl_3$): δ 7.85 (s, 1H), 7.15–7.11 (m, 2H), 6.67 (dd, J = 6.9, 1.7 Hz, 1H), 4.36 (q, J = 7.1 Hz, 2H), 3.97 (s, 3H), 1.71 (sept, J = 7.5 Hz, 3H), 1.40 (t, J = 7.2 Hz, 3H), 1.15 (d, J = 8.0 Hz, 18H).
^{13}C NMR (125 MHz, $CDCl_3$): δ 164.8, 154.1, 143.3, 137.9, 123.3, 118.2, 111.3, 107.4, 102.8, 59.9, 55.6, 18.0, 14.5, 12.7.

4. Evaluation of various reaction parameters

General procedure: An oven-dried J-young Schlenk tube (ca. 13 mL) fitted with a septum was charged with (1,5-cyclooctadiene)(methoxy)iridium(I) dimer (1.3 mg, 2 μmol, 2 mol%) and ligand (4 μmol, 4 mol%), then it was evacuated and purged with nitrogen gas three times. Subsequently, **1a** (13.8 mg, 0.10 mmol) and solvent (1.0 mL) were added via syringe under a nitrogen atmosphere. Next, pinacolborane (25.6 mg, 0.20 mmol) was added via microsyringe and the reaction mixture was stirred at the target temperature for 16 h. After cooling to room temperature, the reaction mixture was diluted with EtOAc. A sample of the crude mixture was passed through a plug of Florisil with EtOAc for GC analysis. The yields were estimated by GC analysis with hexadecane as an internal standard, after calibration of the response curves.

Supplementary Table 1. Investigation of key reaction parameters

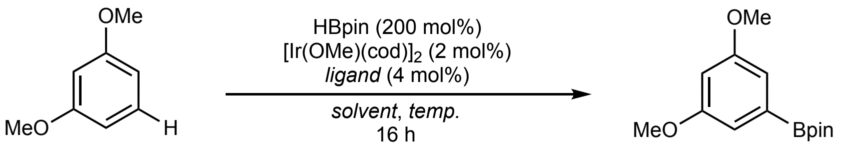

1a (0.1 mmol) **2a**

entry	ligand	solvent	temperature (°C)	**2a** (%)	**1a** (%)
1	**SpiroBpy**	1,4-dioxane	90	75	15
2	**SpiroBpy**	1,4-dioxane	70	73	18
3	**SpiroBpy**	1,4-dioxane	50	81	10
4	**SpiroBpy**	THF	50	82 (78)	13
5[a]	**SpiroBpy**	THF	50	60	31
6[b]	**SpiroBpy**	THF	50	56	38
7	**SpiroBpy**	THF	40	59	34
8	**SpiroBpy**	THF	30	34	59
9	**SpiroBpy**	hexane	40	56	36
10	**SpiroBpy**	CyMe	40	58	35
11	**SpiroBpy**	CyH	40	65	26
12	**SpiroBpy**	CyH	50	79	9
13	**dtbpy**	THF	50	20	78
14	**tmphen**	THF	50	50	44
15	**Bpin-SpiroBpy**	THF	50	48	47
16	**Ph-SpiroBpy**	THF	50	61	31
17	**^{t}Bu-SpiroBpy**	THF	50	80	8
18	**L1** 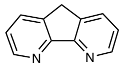	THF	50	26	69
19	**L2** 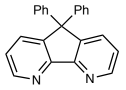	THF	50	65	23

[a]8 h. [b]HBpin 120 mol%.

5. Kinetic studies

Procedure: An oven-dried J-young Schlenk tube (ca. 13 mL) fitted with a septum was charged with $[Ir(OMe)(cod)]_2$ (1.3 mg, 2 µmol, 2 mol%) and ligand (4 µmol, 4 mol%), then it was evacuated and purged with nitrogen gas three times. Subsequently, arene **1** (0.10 mmol), hexadecane (7–15 mg), and solvent (1.0 mL) were added via syringe under a nitrogen atmosphere. Next, pinacolborane (25.6 mg, 0.20 mmol) was added via microsyringe and the reaction mixture was stirred at 50 or 70 °C. At the target time, the Schlenk tube was opened under a stream of N_2 and a sample of the reaction mixture (10 µL) was taken by a microsyringe. The sample was passed with EtOAc through a plug of Florisil into a GC vial. The yields were estimated by GC analysis with hexadecane as an internal standard, after calibration of the response curves.

Comment: the yields estimated during these experiments were slightly higher than those obtained under the standard conditions (closed reaction vessel). We speculate that reaction sampling may release accumulated hydrogen and slightly boost the conversion.[21]

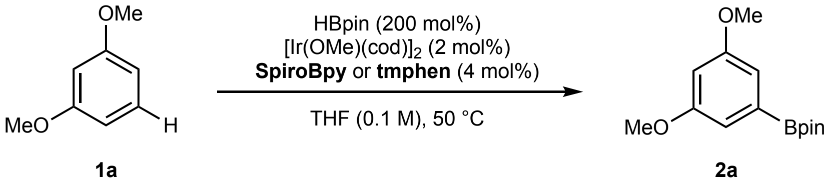

SpiroBpy

time	**2a** (mol/L)	**1a** (mol/L)
5 min	0.0005	0.1020
10 min	0.0011	0.0999
15 min	0.0018	0.0987
30 min	0.0038	0.0970
1 h	0.0079	0.0921
2 h	0.0182	0.0793
4 h	0.0427	0.0498
8 h	0.0732	0.0154

tmphen

time	**2a** (mol/L)	**1a** (mol/L)
5 min	0.0003	0.1040
10 min	0.0005	0.1023
15 min	0.0007	0.1027
30 min	0.0018	0.0989
1 h	0.0041	0.0985
2 h	0.0087	0.0907
4 h	0.0193	0.0795
8 h	0.0371	0.0591

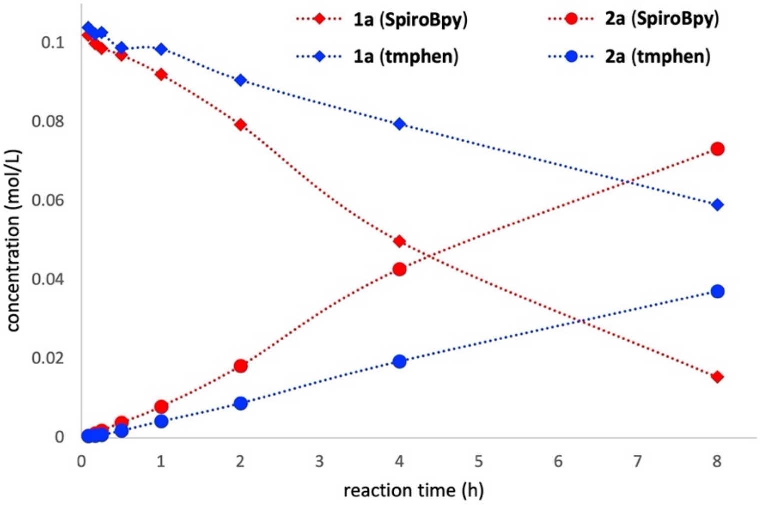

Supplementary Figure 1. Reaction profile of borylation of **1a** with **SpiroBpy** and **tmphen**

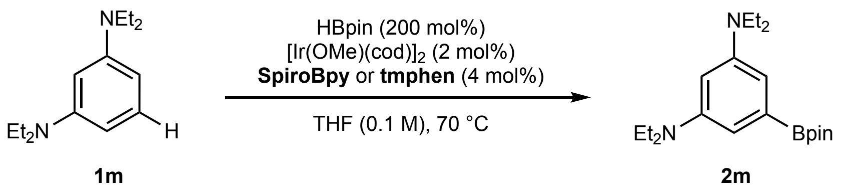

SpiroBpy		
time	**2m** (mol/L)	**1m** (mol/L)
10 min	0.0031	0.0892
30 min	0.0099	0.0834
1 h	0.0231	0.0703
2 h	0.0484	0.0465
4 h	0.0775	0.0223
8 h	0.0941	0.0077

tmphen		
time	**2m** (mol/L)	**1m** (mol/L)
10 min	0.0005	0.0946
30 min	0.0016	0.0932
1 h	0.0032	0.0935
2 h	0.0063	0.0906
4 h	0.0125	0.0832
8 h	0.0243	0.0696

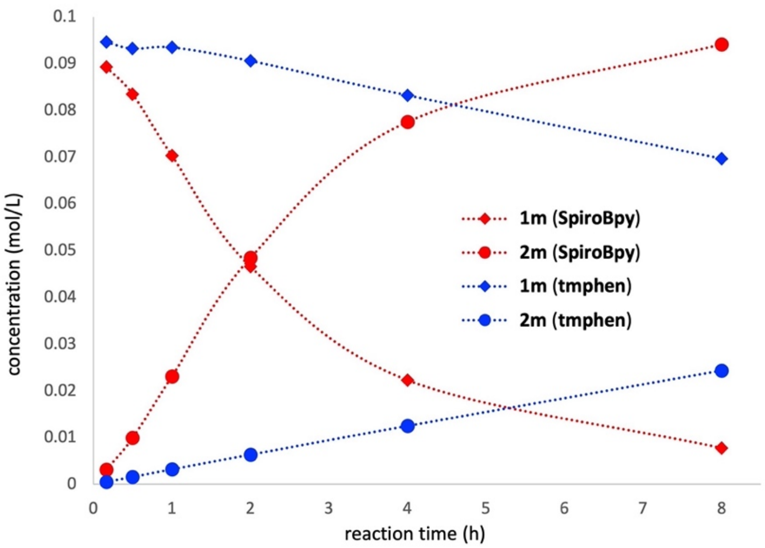

Supplementary Figure 2. Reaction profile of borylation of **1m** with **SpiroBpy** and **tmphen**

6. Iridium-catalyzed C–H borylation of electron-rich arenes

General procedure: An oven-dried J-young Schlenk tube (ca. 13 mL) fitted with a septum was charged with (1,5-cyclooctadiene)(methoxy)iridium(I) dimer (1.3 mg, 2 μmol, 2 mol%) and **SpiroBpy** (1.3 mg, 4 μmol, 4 mol%), then it was evacuated and purged with nitrogen gas three times. When the arene substrate **1** (0.10 mmol) was a solid, it was also added together with $[Ir(OMe)(cod)]_2$ and **SpiroBpy**. When the arene substrate **1** (0.10 mmol) was an oil, it was added via syringe under a nitrogen atmosphere. Next, dry THF (1.0 mL) and pinacolborane (25.6 mg, 0.20 mmol) were added via (micro)syringe and the reaction mixture was stirred at 50 °C for 16 h. Upon heating, the reaction mixture turned dark brown and appeared homogeneous. After cooling to room temperature, the reaction mixture was diluted with EtOAc. The yield of **2** was determined by analyzing the crude mixture by GC (with hexadecane as an internal standard) or ^{1}H NMR (with 1,3,5-trimethoxybenzene as an internal standard). After removing all volatiles under reduced pressure, the product was isolated by silica gel column chromatography or gel permeation chromatography (GPC). The reactions using **dtbpy** and **tmphen** as ligands were performed under the same conditions using the same procedure with the one described above for **SpiroBpy** as the ligand. The yields were determined by GC (with hexadecane as an internal standard) or ^{1}H NMR (with 1,3,5-trimethoxybenzene as an internal standard). Comment: For the reactions using **tmphen**, especially for low yielding substrates, we sometimes observed variation in yields when using different batches of Ir precursor or HBpin. For those cases, we report the yields as average of two runs. We did not observe significant variations in yield when **SpiroBpy** was used as the ligand. Exposure of the reaction mixture to atmospheric air, even for a short time (1 min), decreased the yield by 10–20%.

2-(3,5-Dimethoxyphenyl)-4,4,5,5-tetramethyl-1,3,2-dioxaborolane (2a) [365564-07-4]

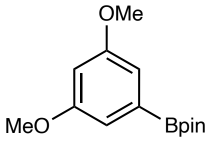

The reaction was conducted following the general procedure using 1,3-dimethoxybenzene **1a** (13.8 mg, 0.10 mmol) as the substrate. The yield of the product was determined by GC analysis of the crude mixture using hexadecane as an internal standard to be 82% yield, after calibration. The crude mixture was purified by GPC (eluent: $CHCl_3$) to afford the title compound **2a** as a colorless solid (20.6 mg, 78 μmol, 78%). The spectral data of product **2a** was in good agreement with the literature.[22]

^{1}H NMR (500 MHz, $CDCl_3$): δ 6.95 (d, J = 2.3 Hz, 2H), 6.57 (t, J = 2.6 Hz, 1H), 3.82 (s, 6H), 1.34 (s, 12H).

^{13}C NMR (125 MHz, $CDCl_3$): δ 160.4, 111.6, 104.5, 83.9, 55.4, 24.8.

2-(3,4-Dimethoxyphenyl)-4,4,5,5-tetramethyl-1,3,2-dioxaborolane (2b) [365564-10-9]

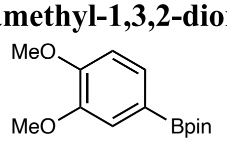

The reaction was conducted following the general procedure using 1,2-dimethoxybenzene **1b** (13.8 mg, 0.10 mmol) as the substrate. The yield of the product was determined by GC analysis of the crude mixture using hexadecane as an internal standard to be >99% yield, after calibration. The crude mixture was purified by silica gel column chromatography (eluent: hexane/EtOAc = 5/1) to

afford the title compound **2b** as a colorless solid (18.9 mg, 72 μmol, 72%). The spectral data of product **2b** was in good agreement with the literature.[23]
^{1}H NMR (500 MHz, $CDCl_3$): δ 7.43 (dd, *J* = 8.0, 1.1 Hz, 1H), 7.29 (d, *J* = 1.1 Hz, 1H), 6.89 (d, *J* = 8.0 Hz, 1H), 3.93 (s, 3H), 3.91 (s, 3H), 1.34 (s, 12H).
^{13}C NMR (125 MHz, $CDCl_3$): δ 151.6, 148.3, 128.5, 116.5, 110.5, 83.6, 55.8, 55.7, 24.8.

4,4,5,5-Tetramethyl-2-(3,4,5-trimethoxyphenyl)-1,3,2-dioxaborolane (2c) [214360-67-5]

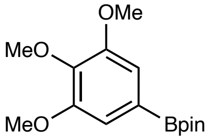

The reaction was conducted following the general procedure using 1,2,3-trimethoxybenzene **1c** (17.0 mg, 0.10 mmol) as the substrate. The yield of the product was determined by ^{1}H NMR analysis of the crude mixture using 1,3,5-trimethoxybenzene as an internal standard to be 93% yield. The crude mixture was purified by GPC (eluent: $CHCl_3$) to afford the title compound **2c** as a colorless solid (25.5 mg, 87 μmol, 86%). The spectral data of product **2c** was in good agreement with the literature.[22]
^{1}H NMR (500 MHz, $CDCl_3$): δ 7.04 (s, 2H), 3.91 (s, 6H), 3.87 (s, 3H), 1.35 (s, 12H).
^{13}C NMR (125 MHz, $CDCl_3$): δ 152.9, 140.8, 111.2, 83.9, 60.8, 56.1, 24.8.

2-(4-Methoxy-3,5-dimethylphenyl)-4,4,5,5-tetramethyl-1,3,2-dioxaborolane (2d) [568572-19-0]

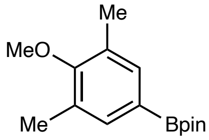

The reaction was conducted following the general procedure using 2-methoxy-1,3-dimethylbenzene **1d** (13.6 mg, 0.10 mmol) as the substrate at 70 °C. The yield of the product was determined by ^{1}H NMR analysis of the crude mixture using 1,3,5-trimethoxybenzene as an internal standard to be 83% yield. The crude mixture was purified by GPC (eluent: $CHCl_3$) to afford the title compound **2d** as a colorless solid (21.0 mg, 80 μmol, 80%). The spectral data of product **2d** was in good agreement with the literature.[21]
^{1}H NMR (500 MHz, $CDCl_3$): δ 7.49 (s, 2H), 3.73 (s, 3H), 2.29 (s, 6H), 1.33 (s, 12H).
^{13}C NMR (125 MHz, $CDCl_3$): δ 159.8, 135.6, 130.3, 83.6, 59.5, 24.8, 15.9.

Triisopropyl(3-methyl-5-(4,4,5,5-tetramethyl-1,3,2-dioxaborolan-2-yl)phenoxy)silane (2e)

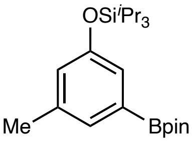

The reaction was conducted following the general procedure using 2-methoxy-1,3-dimethylbenzene **1e** (26.4 mg, 0.10 mmol) as the substrate. The yield of the product was determined by ^{1}H NMR analysis of the crude mixture using 1,3,5-trimethoxybenzene as an internal standard to be 80% yield. The crude mixture was purified by GPC (eluent: $CHCl_3$) to afford the title compound **2e** as a colorless oil (29.6 mg, 76 μmol, 76%).

^{1}H NMR (500 MHz, $CDCl_3$): δ 7.20 (s, 1H), 7.10 (d, J = 2.3 Hz, 1H), 6.77 (t, J = 1.7 Hz, 1H), 2.29 (s, 3H), 1.25 (sept, J = 7.4 Hz, 3H), 1.10 (d, 6.9H).
^{13}C NMR (125 MHz, $CDCl_3$): δ 155.5, 138.6, 128.0, 123.3, 122.8, 83.6, 24.8, 21.2, 18.0, 12.7.
HRMS (APCI) *m*/*z*: $[M + H]^+$ Calcd for $C_{22}H_{40}BO_3Si$ 391.2834; Found 391.2828.

2-(4,4''-Di-*tert*-butyl-[1,1':3',1''-terphenyl]-5'-yl)-4,4,5,5-tetramethyl-1,3,2-dioxaborolane (2f)

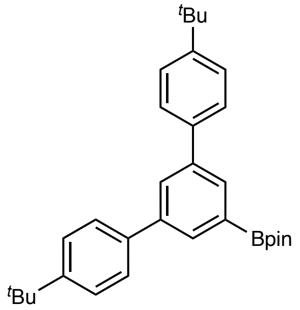

The reaction was conducted following the general procedure using 4,4''-di-*tert*-butyl-1,1':3',1''-terphenyl **1f** (34.3 mg, 0.10 mmol) as the substrate. The yield of the product was determined by ^{1}H NMR analysis of the crude mixture using 1,3,5-trimethoxybenzene as an internal standard to be >99% yield. The crude mixture was purified by GPC (eluent: $CHCl_3$) to afford the title compound **2f** as a colorless solid (46.9 mg, 0.10 mmol, quant.).
^{1}H NMR (500 MHz, $CDCl_3$): δ 8.00 (d, J = 2.3 Hz, 2H), 7.90 (t, J = 1.7 Hz, 1H), 7.63–7.61 (m, 4H), 7.48–7.46 (m, 4H), 1.37 (s, 30H).
^{13}C NMR (125 MHz, $CDCl_3$): δ 150.2, 140.9, 138.3, 132.2, 128.6, 127.0, 125.6, 83.8, 34.5, 31.4, 24.9.
HRMS (APCI) *m*/*z*: $[M + H]^+$ Calcd for $C_{32}H_{42}BO_2$ 469.3272; Found 469.3273.
Melting point: 250–251 °C.

2-(3,5-Dimethylphenyl)-4,4,5,5-tetramethyl-1,3,2-dioxaborolane (2g) [325142-93-6]

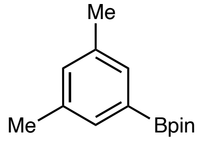

The reaction was conducted following the general procedure using *m*-xylene **1g** (10.6 mg, 0.10 mmol) as the substrate at 60 °C. The yield of the product was determined by ^{1}H NMR analysis of the crude mixture using 1,3,5-trimethoxybenzene as an internal standard to be 76% yield. The crude mixture was purified by GPC (eluent: $CHCl_3$) to afford the title compound **2g** as a colorless solid (16.3 mg, 70 μmol, 70%). The spectral data of product **2g** was in good agreement with the literature.[24]
^{1}H NMR (500 MHz, $CDCl_3$): δ 7.44 (s, 2H), 7.10 (s, 1H), 2.32 (s, 6H), 1.34 (s, 12H).
^{13}C NMR (125 MHz, $CDCl_3$): δ 137.1, 133.0, 132.4, 83.7, 24.8, 21.1.

2-(3,4-Dimethylphenyl)-4,4,5,5-tetramethyl-1,3,2-dioxaborolane (2h) [401797-00-0]

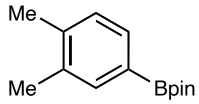

The reaction was conducted following the general procedure using *o*-xylene **1h** (10.6 mg, 0.10 mmol) as the substrate. The yield of the product was determined by ^{1}H NMR analysis of the crude mixture using 1,3,5-trimethoxybenzene as an internal standard to be 72% yield. The crude mixture was purified by GPC (eluent: $CHCl_3$) to afford the title compound **2h** as colorless oil (16.0 mg, 69 μmol, 69%). The spectral data of product **2h** was in good agreement with the literature.[24]
^{1}H NMR (500 MHz, $CDCl_3$): δ 7.58 (s, 1H), 7.55 (d, *J* = 7.4 Hz, 1H), 7.15 (d, *J* = 7.4 Hz, 1H), 2.28 (s, 3H), 2.27 (s, 3H), 1.34 (s, 12H).
^{13}C NMR (125 MHz, $CDCl_3$): δ 140.1, 135.91, 135.87, 132.4, 129.1, 83.5, 24.8, 20.0, 19.5.

2-(3,5-Diisopropylphenyl)-4,4,5,5-tetramethyl-1,3,2-dioxaborolane (2i) [1025719-26-9]

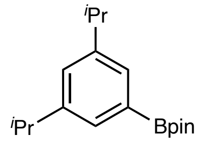

The reaction was conducted following the general procedure using 1,3-diisopropylbenzene **1i** (16.2 mg, 0.10 mmol) as the substrate. The yield of the product was determined by GC analysis of the crude mixture using hexadecane as an internal standard to be 92% yield, after calibration. The crude mixture was purified by silica gel column chromatography (eluent: hexane/EtOAc = 30/1) to afford the title compound **2i** as a colorless solid (23.9 mg, 83 μmol, 83%). The spectral data of product **2i** was in good agreement with the literature.[21]
^{1}H NMR (500 MHz, $CDCl_3$): δ 7.51 (s, 1H), 7.50 (s, 1H), 7.19–7.18 (m, 1H), 2.91 (sept, *J* = 6.9 Hz, 2H), 1.35 (s, 12H), 1.26 (d, *J* = 6.9 Hz, 6H).
^{13}C NMR (125 MHz, $CDCl_3$): δ 148.1, 130.4, 127.7, 83.5, 34.2, 24.8, 24.1.

2-(3,5-Di-*tert*-butylphenyl)-4,4,5,5-tetramethyl-1,3,2-dioxaborolane (2j) [1071924-13-4]

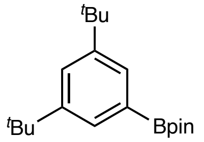

The reaction was conducted following the general procedure using 1,3-di-*tert*-butylbenzene **1j** (18.7 mg, 0.10 mmol) as the substrate. The yield of the product was determined by ^{1}H NMR analysis of the crude mixture using 1,3,5-trimethoxybenzene as an internal standard to be 96% yield. The crude mixture was purified by silica gel column chromatography (eluent: hexane/EtOAc = 25/1) to afford the title compound **2j** as a colorless solid (27.0 mg, 85 μmol, 87%). The spectral data of product **2j** was in good agreement with the literature.[3]
^{1}H NMR (500 MHz, $CDCl_3$): δ 7.67 (d, *J* = 1.7 Hz, 2H), 7.55 (t, *J* = 2.0 Hz, 1H), 1.344 (s, 18H), 1.339 (s, 12H).
^{13}C NMR (125 MHz, $CDCl_3$): δ 149.8, 128.8, 125.5, 83.5, 34.8, 31.5, 24.9.

(5-(4,4,5,5-Tetramethyl-1,3,2-dioxaborolan-2-yl)-1,3-phenylene)bis(trimethylsilane) (2k) [1111096-37-7]

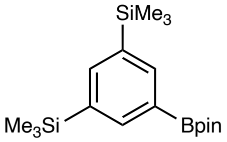

The reaction was conducted following the general procedure using 1,3-bis(trimethylsilyl)benzene **1k** (22.3 mg, 0.10 mmol) as the substrate. The yield of the product was determined by ^{1}H NMR analysis of the crude mixture using 1,3,5-trimethoxybenzene as an internal standard to be >99% yield. The crude mixture was purified by GPC (eluent: $CHCl_3$) to afford the title compound **2k** as a colorless solid (35.0 mg, 0.10 mmol, quant). The spectral data of product **2k** was in good agreement with the literature.[25]
^{1}H NMR (500 MHz, $CDCl_3$): δ 7.96 (d, J = 1.7 Hz, 2H), 7.76 (t, J = 1.4 Hz, 1H), 1.35 (s, 12H), 0.28 (s, 18H).
^{13}C NMR (125 MHz, $CDCl_3$): δ 141.1, 140.3, 138.3, 83.6, 24.8, –1.0.

***N*,*N*,3-Trimethyl-5-(4,4,5,5-tetramethyl-1,3,2-dioxaborolan-2-yl)aniline (2l) [1433982-97-8]**

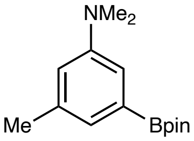

The reaction was conducted following the general procedure using *N*,*N*,3-trimethylaniline **1l** (13.5 mg, 0.10 mmol) as the substrate at 70 °C. The yield of the product was determined by ^{1}H NMR analysis of the crude mixture using 1,3,5-trimethoxybenzene as an internal standard to be 93% yield. The crude mixture was purified by GPC (eluent: $CHCl_3$) to afford the title compound **2l** as a colorless solid (22.7 mg, 87 μmol, 87%). The spectral data of product **2l** was in good agreement with the literature.[21]
^{1}H NMR (500 MHz, $CDCl_3$): δ 7.02 (s, 1H), 7.01 (d, J = 2.9 Hz, 1H), 6.68 (s, 1H), 2.95 (s, 6H), 2.32 (s, 3H), 1.34 (s, 12H).
^{13}C NMR (125 MHz, $CDCl_3$): δ 150.4, 138.1, 124.1, 116.7, 115.9, 83.5, 40.9, 24.8, 21.6.

***N*^{1},*N*^{1},*N*^{3},*N*^{3}-Tetraethyl-5-(4,4,5,5-tetramethyl-1,3,2-dioxaborolan-2-yl)benzene-1,3-diamine (2m)**

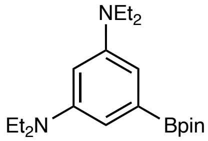

The reaction was conducted following the general procedure using *N*^{1},*N*^{1},*N*^{3},*N*^{3}-tetraethylbenzene-1,3-diamine **1m** (22.0 mg, 0.10 mmol) as the substrate at 70 °C. The yield of product was determined by ^{1}H NMR analysis of the crude mixture using 1,3,5-trimethoxybenzene as an internal standard to be 78% yield. The crude mixture was purified by GPC (eluent: $CHCl_3$) to afford the title compound **2m** as a colorless solid (24.6 mg, 71 μmol, 71%).
^{1}H NMR (500 MHz, $CDCl_3$): δ 6.55 (d, J = 2.3 Hz, 2H), 6.15 (t, J = 2.3 Hz, 1H), 3.35 (q, J = 7.1 Hz, 8H), 1.31 (s, 12H), 1.15 (t, J = 6.9 Hz, 12H).
^{13}C NMR (125 MHz, $CDCl_3$): δ 148.6, 107.5, 100.2, 83.2, 44.4, 24.8, 12.8.
HRMS (APCI) *m*/*z*: $[M + H]^+$ Calcd for $C_{20}H_{36}BN_2O_2$ 347.2864; Found 347.2862.
Melting point: 122–123 °C.

1,1'-(5-(4,4,5,5-Tetramethyl-1,3,2-dioxaborolan-2-yl)-1,3-phenylene)dipiperidine (2n)

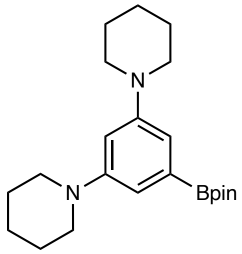

The reaction was conducted following the general procedure using 1,3-di(piperidin-1-yl)benzene **1n** (24.4 mg, 0.10 mmol) as the substrate at 70 °C. The yield of the product was determined by ^{1}H NMR analysis of the crude mixture using 1,3,5-trimethoxybenzene as an internal standard to be 68% yield. The crude mixture was purified by GPC (eluent: $CHCl_3$) to afford the title compound **2n** as a colorless solid (22.3 mg, 60 μmol, 60%).
^{1}H NMR (500 MHz, $CDCl_3$): δ 6.93 (d, *J* = 2.3 Hz, 2H), 6.66 (s, 1H), 3.14 (t, *J* = 5.4 Hz, 8H), 1.72–1.67 (m, 8H), 1.57–1.53 (m, 4H), 1.32 (s, 12H).
^{13}C NMR (125 MHz, $CDCl_3$): δ 152.9, 115.0, 109.6, 83.5, 51.3, 26.0, 24.8, 24.3.
HRMS (APCI) *m*/*z*: $[M + H]^+$ Calcd for $C_{22}H_{36}BN_2O_2$ 371.2864; Found 371.2859.
Melting point: 183–184 °C.

***N*¹,*N*¹,*N*²,*N*²-Tetramethyl-4-(4,4,5,5-tetramethyl-1,3,2-dioxaborolan-2-yl)benzene-1,2-diamine (2o)**

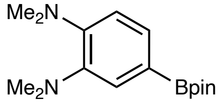

The reaction was conducted following the general procedure using N^1,N^1,N^2,N^2-tetramethylbenzene-1,2-diamine **1o** (15.8 mg, 0.10 mmol) as the substrate. The yield of the product was determined by ^{1}H NMR analysis of the crude mixture using 1,3,5-trimethoxybenzene as an internal standard to be 88% yield. The crude mixture was purified by GPC (eluent: $CHCl_3$) to afford the title compound **2o** as a colorless solid (23.5 mg, 81 μmol, 84%).
^{1}H NMR (500 MHz, $CDCl_3$): δ 7.39 (dd, *J* = 7.7, 1.4 Hz, 1H), 7.33 (d, *J* = 1.1 Hz, 1H), 6.86 (d, *J* = 8.0 Hz, 1H), 2.84 (s, 6H), 2.78 (s, 6H), 1.32 (s, 12H).
^{13}C NMR (125 MHz, $CDCl_3$): δ 148.0, 144.0, 129.1, 124.1, 117.0, 83.3, 41.3, 41.0, 24.8.
HRMS (APCI) *m*/*z*: $[M + H]^+$ Calcd for $C_{16}H_{28}BN_2O_2$ 291.2238; Found 291.2233.
Melting point: 115–116 °C.

1,4-Dimethyl-6-(4,4,5,5-tetramethyl-1,3,2-dioxaborolan-2-yl)-1,2,3,4-tetrahydroquinoxaline (2p)

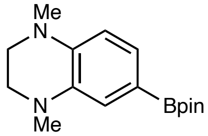

The reaction was conducted following the general procedure using 1,4-dimethyl-1,2,3,4-tetrahydroquinoxaline **1p** (16.0 mg, 0.10 mmol) as the substrate at 70 °C. The yield of the product was determined by ^{1}H NMR analysis of the crude mixture using 1,3,5-trimethoxybenzene as an internal standard to be 76% yield. The crude mixture was purified by GPC (eluent: $CHCl_3$) to afford the title compound **2p** as a colorless solid (18.5 mg, 64 μmol, 65%).

^{1}H NMR (500 MHz, $CDCl_3$): δ 7.21 (dd, J = 7.7, 1.4 Hz, 1H), 6.94 (s, 1H), 6.50 (d, J = 8.0 Hz, 1H), 3.41 (dd, J = 5.4, 4.3 Hz, 2H), 3.25 (dd, J = 5.7, 4.6 Hz, 2H), 2.90 (s, 6H), 1.31 (s, 12H).
^{13}C NMR (125 MHz, $CDCl_3$): δ 139.6, 135.7, 126.7, 116.4, 109.4, 83.0, 50.0, 49.6, 39.6, 38.8, 24.8.
HRMS (APCI) *m*/*z*: $[M + H]^+$ Calcd for $C_{16}H_{26}BN_2O_2$ 289.2082; Found 289.2077.
Melting point: 128–129 °C.

3,6-Di-*tert*-butyl-9-(3-methoxy-5-(4,4,5,5-tetramethyl-1,3,2-dioxaborolan-2-yl)phenyl)-9*H*-carbazole (2q)

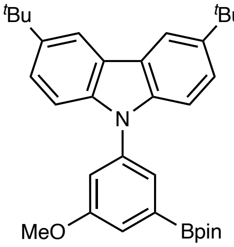

The reaction was conducted following the general procedure using 3,6-di-*tert*-butyl-9-(3-methoxyphenyl)-9*H*-carbazole **1q** (38.6 mg, 0.10 mmol) as the substrate at 70 °C. The yield of the product was determined by ^{1}H NMR analysis of the crude mixture using 1,3,5-trimethoxybenzene as an internal standard to be 86% yield. The crude mixture was purified by GPC (eluent: $CHCl_3$) to afford the title compound **2q** as a colorless solid (36.5 mg, 71 μmol, 71%).
^{1}H NMR (500 MHz, $CDCl_3$): δ 8.12 (d, J = 1.4 Hz, 2H), 7.58 (d, J = 1.1 Hz, 1H), 7.45 (dd, J = 8.9, 2.0 Hz, 2H), 7.39 (d, J = 1.7 Hz, 1H), 7.35 (d, J = 8.6 Hz, 2H), 7.18–7.17 (m, 1H), 3.88 (s, 3H), 1.46 (s, 18H), 1.34 (s, 12H).
^{13}C NMR (125 MHz, $CDCl_3$): δ 160.2, 142.6, 139.3, 138.8, 125.4, 123.5, 123.3, 117.8, 116.1, 115.9, 109.4, 84.1, 55.6, 34.7, 32.0, 24.9.
HRMS (APCI) *m*/*z*: $[M + H]^+$ Calcd for $C_{33}H_{43}BNO_3$ 512.3331; Found 512.3331.
Melting point: 180–181 °C.

2-(2-Fluoro-5-methoxyphenyl)-4,4,5,5-tetramethyl-1,3,2-dioxaborolane (2r) [1190129-83-9]

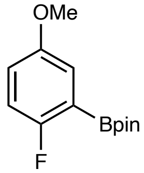

The reaction was conducted following the general procedure using 1-fluoro-4-methoxybenzene **1r** (12.6 mg, 0.10 mmol) as the substrate. The yields of monoborylated product **2r** (54%) and diborylated product **2r'** (25%) were determined by ^{1}H NMR analysis of the crude mixture using 1,3,5-trimethoxybenzene as an internal standard. The crude mixture was purified by GPC (eluent: $CHCl_3$) to afford the title compound **2r** as colorless oil (11.6 mg, 46 μmol, 46%). The spectral data of product **2r** was in good agreement with the literature.[26] **2r'** could not be isolated in pure form.
^{1}H NMR (500 MHz, $CDCl_3$): δ 7.20–7.19 (m, 1H), 6.97–6.93 (m, 2H), 3.80 (s, 3H), 1.36 (s, 12H).
^{13}C NMR (125 MHz, $CDCl_3$): δ 161.6 (d, $J_{C–F}$ = 243.5 Hz), 155.2, 119.7 (d, $J_{C–F}$ = 8.4 Hz), 119.3 (d, $J_{C–F}$ = 8.4 Hz),116.0 (d, $J_{C–F}$ = 26.4 Hz), 83.9, 55.8, 24.8.

2-(3-Bromo-5-methoxyphenyl)-4,4,5,5-tetramethyl-1,3,2-dioxaborolane (2s) [401797-04-4]

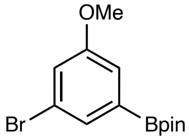

The reaction was conducted following the general procedure using 1-bromo-3-methoxybenzene **1s** (18.7 mg, 0.10 mmol) as the substrate. The yield of the product was determined by ^{1}H NMR analysis of the crude mixture using 1,3,5-trimethoxybenzene as an internal standard to be 90% yield. The crude mixture was purified by GPC (eluent: $CHCl_3$) to afford the title compound **2s** as colorless oil (25.9 mg, 83 μmol, 83%). The spectral data of product **2s** was in good agreement with the literature.[22]

^{1}H NMR (500 MHz, $CDCl_3$): δ 7.52 (d, J = 1.1 Hz, 1H), 7.23 (d, J = 2.3 Hz, 1H), 7.14 (t, J = 2.3 Hz, 1H), 3.81 (s, 3H), 1.34 (s, 12H).

^{13}C NMR (125 MHz, $CDCl_3$): δ 159.9, 129.7, 122.7, 120.6, 117.9, 84.2, 55.5, 24.8.

2-(3-Bromo-5-(*tert*-butyl)phenyl)-4,4,5,5-tetramethyl-1,3,2-dioxaborolane (2t) [1644527-00-3]

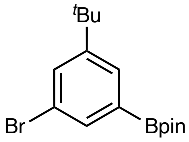

The reaction was conducted following the general procedure using 1-bromo-3-(*tert*-butyl)benzene **1t** (21.2 mg, 0.10 mmol) as the substrate. The yield of the product was determined by ^{1}H NMR analysis of the crude mixture using 1,3,5-trimethoxybenzene as an internal standard to be >99% yield. The crude mixture was purified by GPC (eluent: $CHCl_3$) to afford the title compound **2t** as colorless oil (29.8 mg, 88 μmol, 88%). The spectral data of product **2t** was in good agreement with the literature.[18]

^{1}H NMR (500 MHz, $CDCl_3$): δ 7.75 (d, J = 1.1 Hz, 1H), 7.72 (d, J = 1.7 Hz, 1H), 7.59 (t, J = 1.7 Hz, 1H), 1.34 (s, 12H), 1.32 (s, 9H).

^{13}C NMR (125 MHz, $CDCl_3$): δ 152.9, 134.6, 131.4, 130.0, 122.4, 84.0, 34.9, 31.2, 24.8.

(3-Bromo-5-(4,4,5,5-tetramethyl-1,3,2-dioxaborolan-2-yl)phenyl)trimethylsilane (1u) [1111096-40-2]

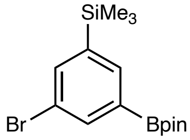

The reaction was conducted following the general procedure using (3-bromophenyl)trimethylsilane **1u** (22.9 mg, 0.10 mmol) as the substrate. The yield of the product was determined by ^{1}H NMR analysis of the crude mixture using 1,3,5-trimethoxybenzene as an internal standard to be >99% yield. The crude mixture was purified by GPC (eluent: $CHCl_3$) to afford the title compound **2u** as a colorless solid (33.0 mg, 93 μmol, 93%). The spectral data of product **2u** was in good agreement with the literature.[27]

^{1}H NMR (500 MHz, $CDCl_3$): δ 7.911–7.906 (m, 1H), 7.822–7.817 (m, 1H), 7.69–7.68 (m, 1H), 1.34 (s, 12H), 0.28 (s, 9H).

^{13}C NMR (125 MHz, $CDCl_3$): δ 143.0, 138.7, 137.79, 137.77, 122.8, 84.1, 24.8, –1.2.\

3-Chloro-*N*,*N*-diethyl-5-(4,4,5,5-tetramethyl-1,3,2-dioxaborolan-2-yl)aniline (2v)

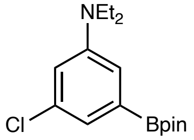

The reaction was conducted following the general procedure using 3-chloro-*N*,*N*-diethylaniline **1v** (18.4 mg, 0.10 mmol) as the substrate. The yield of the product was determined by ^{1}H NMR analysis of the crude mixture using 1,3,5-trimethoxybenzene as an internal standard to be 90% yield. The crude mixture was purified by GPC (eluent: $CHCl_3$) to afford the title compound **2v** as colorless oil (25.9 mg, 84 μmol, 84%).
^{1}H NMR (500 MHz, $CDCl_3$): δ 7.03 (d, J = 2.3 Hz, 1H), 6.96 (d, J = 2.3 Hz, 1H), 6.71 (d, J = 2.3 Hz, 1H), 3.35 (q, J = 7.1 Hz, 4H), 1.33 (s, 12H), 1.15 (t, J = 7.2 Hz, 6H).
^{13}C NMR (125 MHz, $CDCl_3$): δ 148.5, 134.9, 121.1, 115.9, 114.2, 83.8, 44.2, 24.8, 12.5.
HRMS (APCI) *m*/*z*: $[M + H]^+$ Calcd for $C_{16}H_{26}BClNO_2$ 310.1740; Found 310.1743.

1-(3-Chloro-5-(4,4,5,5-tetramethyl-1,3,2-dioxaborolan-2-yl)phenyl)piperidine (2w) [2096334-12-0]

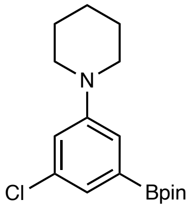

The reaction was conducted following the general procedure using 1-(3-chlorophenyl)piperidine **1w** (19.6 mg, 0.10 mmol) as the substrate. The yield of the product was determined by ^{1}H NMR analysis of the crude mixture using 1,3,5-trimethoxybenzene as an internal standard to be 93% yield. The crude mixture was purified by GPC (eluent: $CHCl_3$) to afford the title compound **2w** as colorless oil (28.0 mg, 87 μmol, 87%).
^{1}H NMR (500 MHz, $CDCl_3$): δ 7.23 (d, J = 1.7 Hz, 1H), 7.20 (d, J = 1.7 Hz, 1H), 6.96 (t, J = 2.3 Hz, 1H), 3.19–3.16 (m, 4H), 1.71–1.66 (m, 4H), 1.60–1.55 (m, 2H), 1.33 (s, 12H).
^{13}C NMR (125 MHz, $CDCl_3$): δ 152.8, 134.6, 124.6, 120.5, 118.7, 83.9, 50.2, 25.7, 24.8, 24.2.
HRMS (APCI) *m*/*z*: $[M + H]^+$ Calcd for $C_{17}H_{26}BClNO_2$ 322.1740; Found 322.1742.

2-(3,5-Dichlorophenyl)-4,4,5,5-tetramethyl-1,3,2-dioxaborolane (2x) [68716-51-8]

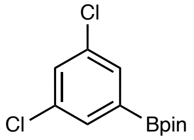

The reaction was conducted following the general procedure using 1,3-dichlorobenzene **1x** (14.7 mg, 0.10 mmol) as the substrate at room temperature. The yield of the product was determined by ^{1}H NMR analysis of the crude mixture using 1,3,5-trimethoxybenzene as an internal standard to be 93% yield. The crude mixture was purified by GPC (eluent: $CHCl_3$) to afford the title compound **2x** as colorless oil (23.3 mg, 85 μmol, 85%). The spectral data of product **2x** was in good agreement with the literature.[28]
^{1}H NMR (500 MHz, $CDCl_3$): δ 7.65 (d, J = 2.3 Hz, 2H), 7.43 (t, J = 2.3 Hz, 1H), 1.34 (s, 12H).
^{13}C NMR (125 MHz, $CDCl_3$): δ 134.7, 132.7, 131.1, 84.5, 24.8.

Unsuccessful Substrates

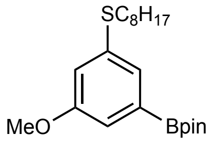

SpiroBpy: n.d.
dtbpy: n.d.
tmphen: n.d.

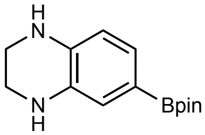

SpiroBpy: n.d.
dtbpy: n.d.
tmphen: n.d.

7. Gram-scale synthesis of pharmaceutically relevant compounds

2-(Diethylamino)-*N*-(2,6-dimethyl-4-(4,4,5,5-tetramethyl-1,3,2-dioxaborolan-2-yl)phenyl)acetamide (4a) [2648559-40-2]

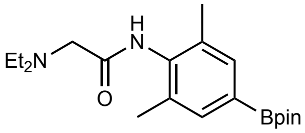

An oven-dried 100 mL Schlenk flask fitted with a septum was charged with Lidocaine (1.00 g, 4.28 mmol), (1,5-cyclooctadiene)(methoxy)iridium(I) dimer (57 mg, 86 μmol, 2.0 mol%), and **SpiroBpy** (55 mg, 172 μmol, 4.0 mol%), then it was evacuated and purged with nitrogen gas three times. Subsequently, THF (21.5 mL) and pinacolborane (1.65 g, 12.9 mmol) were added via syringe under a nitrogen atmosphere. The reaction mixture was stirred at room temperature for 10 min, then heated to 70 °C for 24 h. After cooling to room temperature, to the reaction mixture was added MeOH (5 mL), and then it was stirred for 15 min at room temperature. After removing all volatiles under reduced pressure, the crude mixture was passed through a Florisil pad (eluent: hexane, then $CHCl_3$) to give a yellow oil. To the yellow oil was added small amount of pentane and then it was cooled to –30 °C to result in precipitation of the target product **4a** as a colorless solid (803 mg, 2.23 mmol). After collecting the colorless solid by filtration, the yellowish mother liquor was collected and concentrated to give a yellow residue. The residue was purified by GPC (eluent: $CHCl_3$) to afford a second batch of **4a** as a colorless solid (158 mg, 0.44 mmol). The overall yield of **4a** was calculated to be 62% (961 mg, 2.67 mmol).
^{1}H NMR (500 MHz, $CDCl_3$): δ 8.96 (s, 1H), 7.54, (s, 2H), 3.21 (s, 2H), 2.69 (q, J = 7.1 Hz, 4H), 2.24 (s, 6H), 1.34 (s, 12H), 1.13 (t, J = 7.2 Hz, 6H).
^{13}C NMR (125 MHz, $CDCl_3$): δ 170.1, 136.9, 134.7, 134.1, 83.7, 57.5, 48.9, 24.8, 18.3, 12.6.
HRMS (APCI) m/z: $[M + H]^+$ Calcd for $C_{20}H_{34}BN_2O_3$ 361.2657; Found 361.2656.
Melting point: 129–130 °C.

Methyl 2-((*tert*-butoxycarbonyl)amino)-3-(3-methoxy-5-(4,4,5,5-tetramethyl-1,3,2-dioxaborolan-2-yl)phenyl)propanoate (4b) [2648559-43-5]

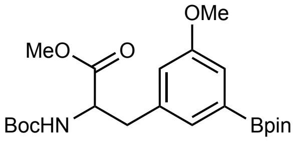

An oven-dried 100 mL Schlenk flask fitted with a septum was charged with methyl 2-((*tert*-butoxycarbonyl)amino)-3-(3-methoxyphenyl)propanoate (1.02 g, 3.30 mmol), (1,5-cyclooctadiene) (methoxy)iridium(I) dimer (44 mg, 66 μmol, 2.0 mol%), and **SpiroBpy** (42 mg, 132 μmol, 4.0 mol%), then it was evacuated and purged with nitrogen gas three times. Subsequently, THF (33 mL) and pinacolborane (1.28 g, 10.0 mmol) were added via syringe under a nitrogen atmosphere. The reaction mixture was stirred at room temperature for 10 min, then heated to 70 °C for 16 h. After cooling to room temperature, to the reaction mixture was added MeOH (5 mL) and it was stirred for 15 min at room temperature. After removing all volatiles under reduced pressure, the crude mixture was purified by silica gel column chromatography (eluent: hexane/$CHCl_3$ = 1/1) to afford the title compound **4b** as a colorless gel (1.38 g, 3.16 mmol, 96%). The spectral data of product **4b** was in good agreement with the literature.[29]

^{1}H NMR (500 MHz, $CDCl_3$): δ 7.20 (d, *J* = 2.3 Hz, 1H), 7.16 (s, 1H), 6.77 (s, 1H), 4.96 (d, *J* = 8.0 Hz, 1H), 4.55 (q, *J* = 6.5 Hz, 1H), 3.81 (s, 3H), 3.72 (s, 3H), 3.12–3.08 (m, 1H), 3.03–2.99 (m, 1H), 1.42 (s, 9H), 1.34 (s, 12H).
^{13}C NMR (125 MHz, $CDCl_3$): δ 172.3, 159.1, 155.0, 136.9, 128.2, 118.7, 117.2, 83.8, 79.8, 55.2, 54.4, 52.1, 37.9, 28.2, 24.8, 24.8.

Ethyl 4-methoxy-6-(4,4,5,5-tetramethyl-1,3,2-dioxaborolan-2-yl)-1-(triisopropylsilyl)-1*H*-indole-3-carboxylate (4c) [2210231-61-9]

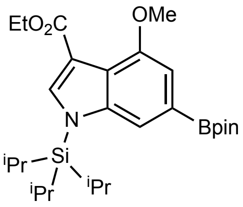

An oven-dried 100 mL Schlenk flask fitted with a septum was charged with ethyl 4-methoxy-1-(triisopropylsilyl)-1*H*-indole-3-carboxylate (1.00 g, 2.66 mmol), (1,5-cyclooctadiene)(methoxy)iridium(I) dimer (35 mg, 53 μmol, 2.0 mol%), and **SpiroBpy** (34 mg, 107 μmol, 4.0 mol%), then it was evacuated and purged with nitrogen gas three times. Subsequently, THF (27 mL) and pinacolborane (683 mg, 5.34 mmol) were added via syringe under a nitrogen atmosphere. The reaction mixture was stirred at 50 °C for 16 h. After cooling to room temperature, all volatiles were removed under reduced pressure. The crude mixture was purified by silica gel column chromatography (eluent: hexane/EtOAc = 10/1 to 5/1) to afford the title compound **4c** as a colorless solid (1.27 g, 2.54 mmol, 95%). The spectral data of product **4c** was in good agreement with the literature.[20]
^{1}H NMR (500 MHz, $CDCl_3$): δ 7.89 (s, 1H), 7.58 (s, 1H), 7.05 (s, 1H), 4.35 (q, *J* = 7.3 Hz, 2H), 4.01 (s, 3H), 1.72 (sept, *J* = 7.5 Hz, 3H), 1.40 (t, *J* = 7.2 Hz, 3H), 1.35 (s, 12H), 1.15 (d, *J* = 7.4 Hz, 18H).
^{13}C NMR (125 MHz, $CDCl_3$): δ 164.9, 153.6, 143.2, 139.2, 120.8, 114.7, 111.5, 107.9, 83.6, 60.0, 55.9, 25.0, 18.2, 14.6, 12.8.

8. Mechanistic studies
Synthesis of SpiroBpy-d_8 ligand

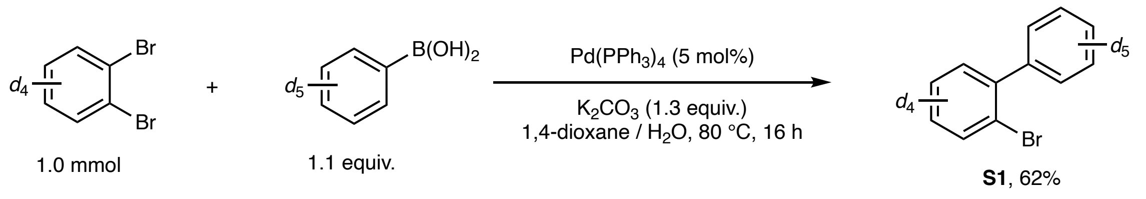

To an oven-dried 50 mL Schlenk flask was added $Pd(PPh_3)_4$ (58 mg, 0.050 mmol), (phenyl-d_5)boronic acid[30] (140 mg, 1.1 mmol), and K_2CO_3 (180 mg, 1.3 mmol) under N_2 atmosphere. Subsequently, 1,4-dioxane (5 mL), H_2O (1.25 mL), and 1,2-dibromobenzene-3,4,5,6-d_4 (240 mg, 1.0 mmol) were added via syringe. The mixture was stirred at 80 °C for 16 h. After cooling to room temperature, the mixture was diluted with $CHCl_3$, and then extracted with $CHCl_3$ three times. The combined organic layer was dried over $MgSO_4$, filtered, and concentrated under reduced pressure. The crude residue was purified by PTLC (eluent: hexane) to afford the target compound 2-bromo-1,1'-biphenyl-2',3,3',4,4',5,5',6,6'-d_9 **S1** as colorless oil (151 mg, 0.62 mmol, 62%).
^{2}H NMR (77 MHz, $CHCl_3$): δ 7.69 (br, 1D), 7.43–7.35 (m, 7D), 7.23 (br, 1D).
^{13}C NMR (125 MHz, $CDCl_3$): δ 142.4, 140.9, 132.7 (t, J = 25.2 Hz), 130.8 (t, J = 24.6 Hz), 128.9 (t, J = 24.6 Hz), 128.2 (t, J = 25.2 Hz), 127.6–126.6 (m), 122.5.
HRMS (APCI) m/z: $[M]^+$ Calcd for $C_{12}D_9Br$ 241.0453; Found 241.0444.

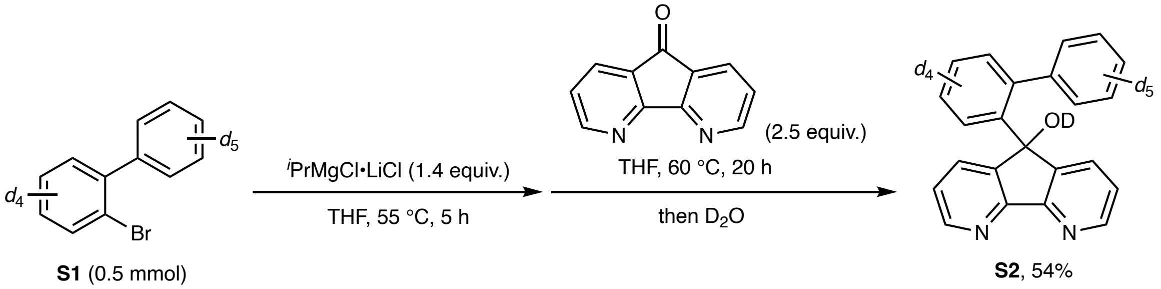

To an oven-dried 50 mL Schlenk flask was added 2-bromo-1,1'-biphenyl-2',3,3',4,4',5,5',6,6'-d_9 **S1** (121 mg, 0.50 mmol) in dry THF (1.0 mL) under N_2. To the solution was added ^{i}PrMgCl•LiCl (1.25 M in THF, 0.56 mL, 0.70 mmol) via syringe at room temperature. The mixture was stirred at 55 °C for 5 h. The generation of the Grignard reagent was confirmed by GC-MS analysis of a small reaction sample quenching with H_2O. To the Grignard solution was added 5*H*-cyclopenta[2,1-*b*:3,4-*b'*]dipyridin-5-one[3] (228 mg, 1.25 mmol) in one portion under N_2 flow at room temperature. The reaction mixture was charged with 5 mL dry THF, and then stirred at 60 °C for 20 h. After cooling to room temperature, to the mixture was added D_2O (ca. 5 mL), and diluted by adding $CHCl_3$. The mixture was filtered through a celite pad, and then extracted with $CHCl_3$ three times. The combined organic layer was dried over $MgSO_4$, filtered, and concentrated under reduced pressure. The crude material was purified by silica gel column chromatography (eluent: hexand/EtOAc = 1/1, then EtOAc), followed by GPC (eluent: $CHCl_3$) to afford the target compound 5-([1,1'-biphenyl]-2-yl-d_9)-5*H*-cyclopenta[2,1-*b*:3,4-*b'*]dipyridin-5-ol-*d* **S2** (93.4 mg, 0.27 mmol, 54%) as an off-white solid.

^1H NMR (500 MHz, $CDCl_3$): δ 8.47 (t, J = 4.6 Hz, 2H), 7.55 (dd, J = 7.4, 1.7 Hz, 2H), 7.14–7.11 (m, 2H).
^2H NMR (77 MHz, $CHCl_3$): δ 8.57 (br, 1D), 7.57 (br, 1D), 7.38 (br, 1D), 6.88 (br, 2D), 6.61 (br, 2D), 5.89 (br, 2D).
^{13}C NMR (125 MHz, $CDCl_3$): δ 157.1, 150.0, 145.9, 140.3, 139.3, 137.6, 132.1, 130.7 (t, J = 24 Hz), 128.1 (t, J = 23.4 Hz), 127.1–125.6 (m), 123.3, 78.3.
HRMS (APCI) m/z: $[M]^+$ Calcd for $C_{23}H_6D_{10}N_2O$ 346.1890; Found 346.1891.

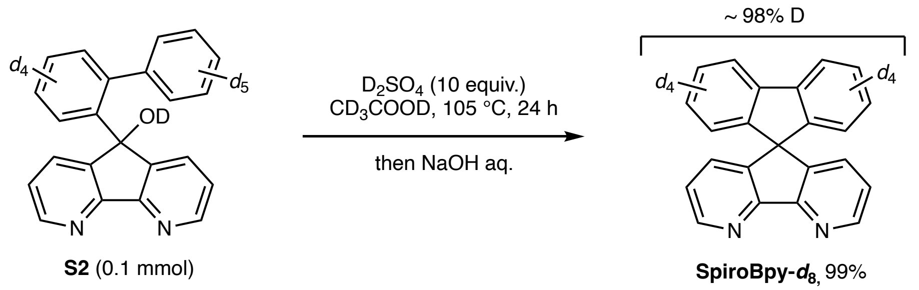

To a J-young Schlenk tube was added 5-([1,1'-biphenyl]-2-yl-d_9)-5H-cyclopenta[2,1-b:3,4-b']dipyridin-5-ol-d **S2** (34.6 mg, 0.10 mmol) under N_2. Subsequently, acetic acid-d_4 (0.5 mL) and D_2SO_4 (98% in D_2O, 1 mmol, 55 μL) were added via syringe. The tube was tightly sealed with a J-young cap and stirred at 105 °C for 24 h. After cooling to room temperature, a light-yellow precipitate formed. The crude material was poured into crushed ice with NaOH aq. to ensure pH > 7. The mixture was extracted with $CHCl_3$ three times. The combined organic layer was dried over $MgSO_4$, filtered, and concentrated under reduced pressure. The crude material was purified by silica gel column chromatography (eluent: EtOAc) to afford the target compound spiro[cyclopenta[2,1-b:3,4-b']dipyridine-5,9'-fluorene]-1',2',3',4',5',6',7',8'-d_8 **SpiroBpy-*d*$_8$** (32.4 mg, 99 μmol, 99%) as a colorless solid.
^1H NMR (500 MHz, $CDCl_3$): δ 8.74 (t, J = 3.2 Hz, 2H), 7.12 (d, J = 2.9 Hz, 4H).
^2H NMR (77 MHz, $CHCl_3$): δ 7.93 (br, 2D), 7.47 (br, 2D), 7.20 (br, 2D), 6.79 (br, 2D).
^{13}C NMR (125 MHz, $CDCl_3$): δ 159.0, 150.4, 146.0, 143.7, 141.8, 131.8, 127.9 (t, J = 24.0 Hz), 127.6 (t, J = 24.0 Hz), 123.6, 123.4 (t, J = 24.6 Hz), 120.0 (t, J = 24.0 Hz), 61.5.
HRMS (APCI) m/z: $[M + H]^+$ Calcd for $C_{23}H_7D_8N_2$ 327.1732; Found 327.1733.

General procedure for the kinetic experiments: SpiroBpy vs SpiroBpy-*d*₈

Stock solution A: In an argon-filled glove box, N^1,N^1,N^3,N^3-tetramethylbenzene-1,3-diamine **5** (684.5 mg, 4.17 mmol), hexadecane (625.1 mg, 2.76 mmol) were placed in a 25 mL measuring flask. Dry dioxane was added to the flask to afford 25 mL of Stock Solution A (0.167 M of arene, 0.110 M of hexadecane in dioxane). The solution was transferred to a 30 mL J-young Schlenk tube and stored at –30 °C under inert atmosphere.

Stock solution B: In an argon-filled glove box, $[Ir(OMe)(cod)]_2$ (82.9 mg, 0.125 mmol) was placed in a 25 mL measuring flask. Dry dioxane was added to the flask to give 25 mL of Stock Solution B (0.0050 M of $[Ir(OMe)(cod)]_2$ in dioxane). The solution was separated and transferred to several 10 mL J-young Schlenk tubes (2–3 mL for each tube) and stored at –30 °C under inert atmosphere.

Stock solution C1: In an argon-filled glove box, **SpiroBpy** (15.9 mg, 0.050 mmol) was accurately weighed and placed in a 5 mL measuring flask. Dry dioxane was added to the flask to give 5 mL of Stock Solution C1 (0.010 M of **SpiroBpy** in dioxane). The solution was transferred to a 10 mL J-young Schlenk tube and stored at –30 °C under inert atmosphere.

Stock solution C2: In an argon-filled glove box, **SpiroBpy-d_8** (16.3 mg, 0.050 mmol) was accurately weighed and placed in a 5 mL measuring flask. Dry dioxane was added to the flask to give 5 mL of Stock Solution C2 (0.010 M of **SpiroBpy-d_8** in dioxane). The solution was transferred to a 10 mL J-young Schlenk tube and stored at –30 °C under inert atmosphere.

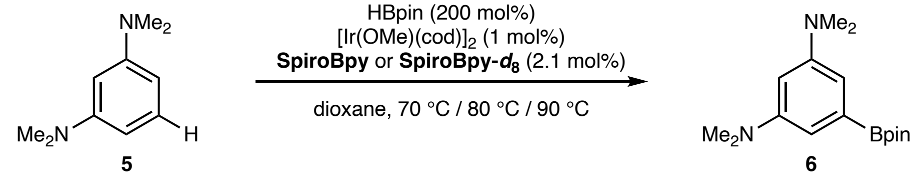

An oven-dried J-young Schlenk tube (ca. 13 mL) fitted with a septum was evacuated and purged with nitrogen gas three times. Subsequently, stock solution A (0.60 mL, 0.10 mmol of arene), stock solution B (200 µL, 1.0 µmol of $[Ir(OMe)(cod)]_2$), and stock solution C1 or C2 (210 µL, 2.1 µmol of ligand) were added via syringe under a nitrogen atmosphere. Next, pinacolborane (29 µL, 25.6 mg, 0.20 mmol) was added via microsyringe and the reaction mixture was stirred at the target temperature. At the target time, the Schlenk tube was opened under a stream of N_2 and a sample of the reaction mixture (10 µL) was taken by a microsyringe. The sample was passed with EtOAc through a plug of Florisil into a GC vial. The yields were estimated by GC analysis with hexadecane as an internal standard, after calibration of the response curves. For these studies, each reaction was performed twice using the same procedure under identical conditions. It should be noted that hydrogen gas is released from these reactions, and therefore the sampling is expected to slightly affect the results because of the release of hydrogen. We attempted the reaction in an NMR tube, but the yields were very low, either because of the different shape/size of the reaction vessel, or because of ineffective stirring.

The ligand kinetic isotope effect (KIE) values were calculated according to the initial reaction rates (average of two runs) to be 0.78 ± 0.04 (70 °C), 0.71 ± 0.03 (80 °C), and 0.67 ± 0.03 (90 °C) (Figures S3 and S4).

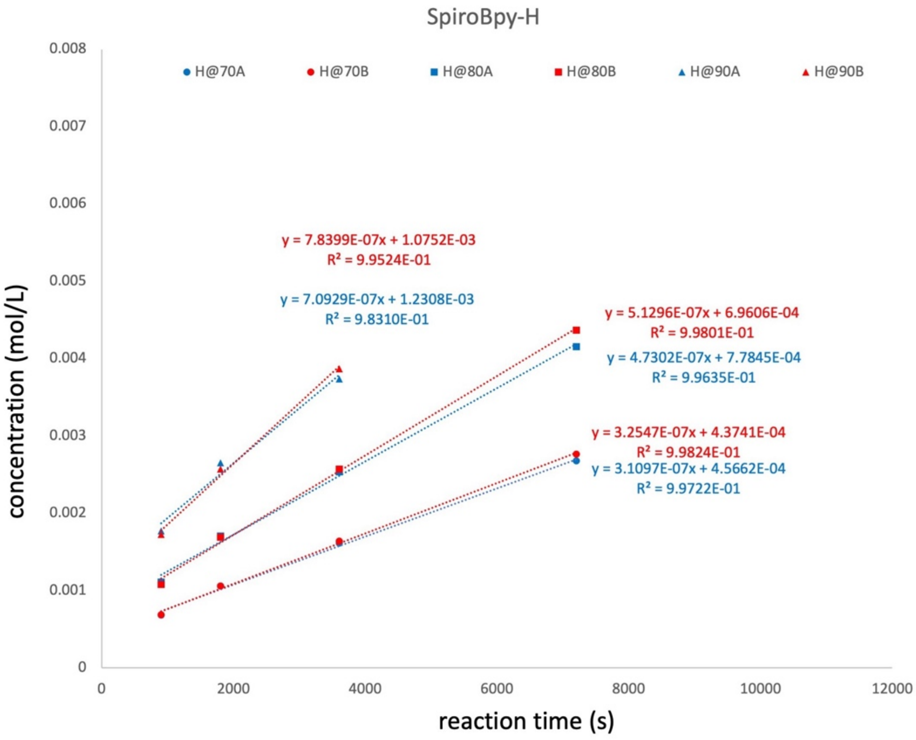

Supplementary Figure 3. Reaction profile of borylation of **5** with **SpiroBpy** at 70 °C, 80 °C, and 90 °C

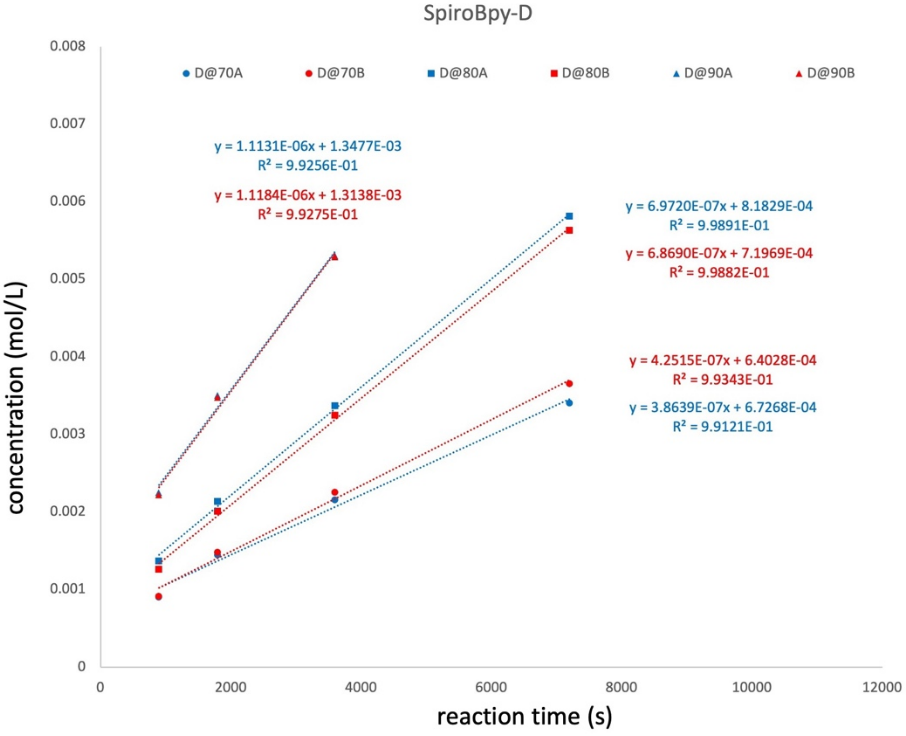

Supplementary Figure 4. Reaction profile of borylation of **5** with **SpiroBpy-*d*$_8$** at 70 °C, 80 °C, and 90 °C

Differential Eyring Analysis

The C–H borylation of **5** using **SpiroBpy** or **SpiroBpy-*d*$_8$** was performed at 70 °C, 80 °C, and 90 °C, respectively. The following Eyring equations hold for **SpiroBpy** (Supplementary equation 1) and **SpiroBpy-*d*$_8$** (Supplementary equation 2).

$$\ln\frac{k_H}{T} = -\frac{\Delta H_H{}^{\ddagger}}{R}\frac{1}{T} + \ln\frac{k_\mathrm{B}}{h} + \frac{\Delta S_H{}^{\ddagger}}{R} \quad \text{(Supplementary equation 1)}$$

$$\ln\frac{k_D}{T} = -\frac{\Delta H_D{}^{\ddagger}}{R}\frac{1}{T} + \ln\frac{k_\mathrm{B}}{h} + \frac{\Delta S_D{}^{\ddagger}}{R} \quad \text{(Supplementary equation 2)}$$

(k: rate constant, T: temperature, k_B: Boltzmann constant, h: Planck constant, R: gas constant, $\Delta H^{\ddagger}$: activation enthalpy, $\Delta S^{\ddagger}$: activation entropy)

Supplementary equation 3 is derived by taking the difference between Supplementary equation 1 and Supplementary equation 2.

$$\ln\frac{k_H}{k_D} = -\frac{\Delta\Delta H^{\ddagger}_{H-D}}{R}\frac{1}{T} + \frac{\Delta\Delta S^{\ddagger}_{H-D}}{R} \quad \text{(Supplementary equation 3)}$$

Assuming that the reaction mechanism remains the same with **SpiroBpy** and **SpiroBpy-*d*$_8$**, the ratio of the reaction rate constants ($k_\mathrm{H}/k_\mathrm{D}$) for the turnover-limiting C–H activation step can be expressed as the ratio of the experimentally determined initial reaction rates ($r_{\mathrm{obs}\ H}/r_{\mathrm{obs}\ D}$) (Supplementary equation 4).

$$\frac{r_{obs\,H}}{r_{obs\,D}} = \frac{k_H[Arene][LIr(Bpin)_3]}{k_D[Arene][LIr(Bpin)_3]} = \frac{k_H}{k_D} \quad \text{(Supplementary equation 4)}$$

Thus, the differences in activation enthalpy ($\Delta\Delta H^{\ddagger}_{\mathrm{H-D}}$) and activation entropy ($\Delta\Delta S^{\ddagger}_{\mathrm{H-D}}$) were calculated by differential Eyring plot based on Supplementary equation 3 and Supplementary Figure 5 to be –2.0±0.8 kcal mol^{-1} and –6.2±2.2 cal mol^{-1} K^{-1}, respectively. The errors were calculated according to the literature.[31] These results suggest that the reaction with **SpiroBpy** is enthalpically more favored but entropically less favored compared with the reaction with **SpiroBpy-*d*$_8$**. Although speculative at the moment, this could be partially explained by the stronger CH–π interaction and shorter CH–π distance, which are a result of the longer C–H bond than the C–D bond and the slightly larger polarizability of hydrogen than that of deuterium.

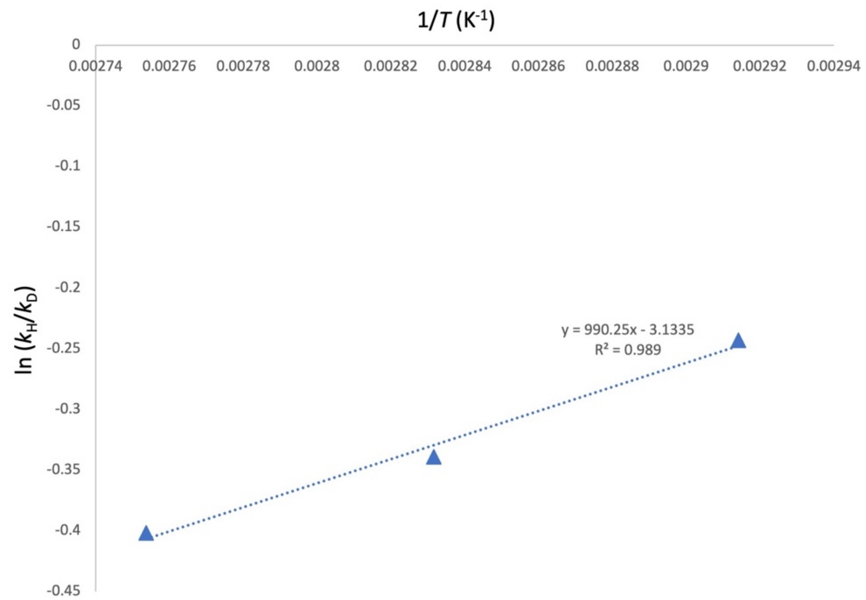

Supplementary Figure 5. Differential Eyring plot.

9. DFT studies

All calculations were performed with the Gaussian 16 program package.[32] Structures were optimized using B3LYP-D3 and a basis set consisting of the Stuttgart–Dresden (SDD) basis set and effective core potential (ECP) for Ir and 6-31+G(d,p) for the rest. Single point calculations were performed on the B3LYP-D3 geometries using M06 functional and a basis set consisting of the Stuttgart–Dresden (SDD) basis set and effective core potential (ECP) for Ir and 6-311+G(d,p) for the rest. Solvent effects were taken into consideration with the SMD method (THF as a solvent) as implemented in Gaussian 16. Each stationary point was adequately characterized by normal coordinate analysis (no imaginary frequencies for an equilibrium structure and one imaginary frequency for a transition structure) and thermal corrections were calculated at 298.15 K. The computational studies were performed using 1,3-$(Me_2N)_2$benzene as a substrate and **L1**, **SpiroBpy**, and **tmphen** as ligands. An independent gradient model based on the Hirshfeld partition (IGMH) analysis (fragment 1, SpiroBpy; fragment 2, $IrBpin_3$_arene)[33] and non-covalent interaction (NCI) analysis[34] were performed with Multiwfn 3.8[35] with the high-quality grid option and visualized by VMD software.[36]

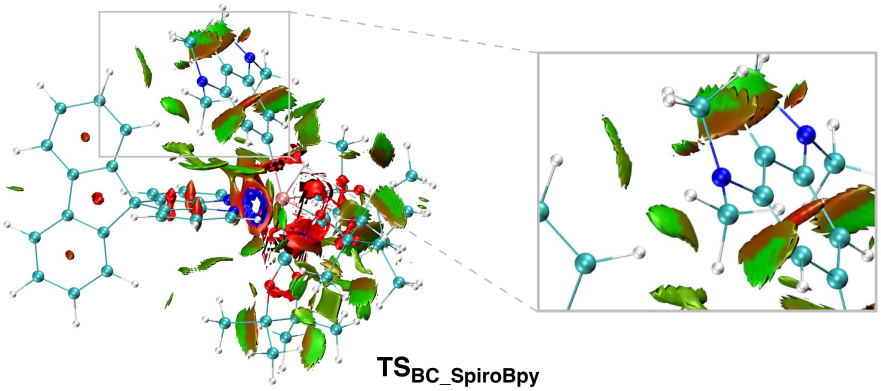

$TS_{BC\_SpiroBpy}$

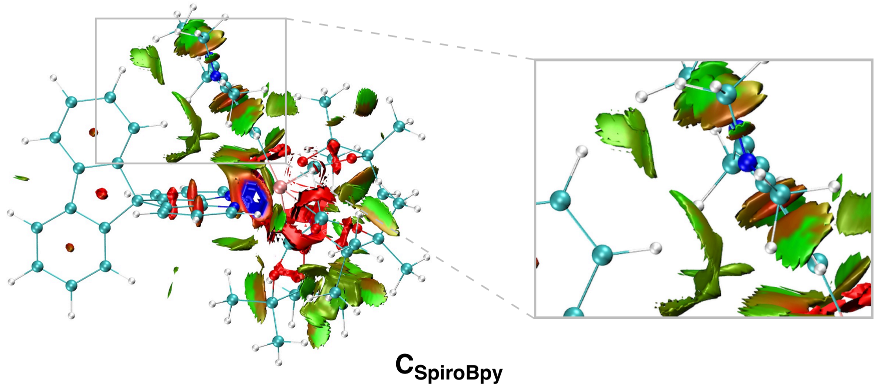

$C_{SpiroBpy}$

Supplementary Figure 6. Noncovalent interaction plots for **$TS_{BC\_SpiroBpy}$** and **$C_{SpiroBpy}$**. The surfaces are colored on a blue-green-red scale with a reduced density gradient (RDG) = 0.6 au, according to values of sign(λ_2)ρ ranging from –0.03 to 0.03 au. The blue and red region indicates strong attractive interactions and repulsive interactions, respectively.

An NBO analysis (ver 7.0)[37] indicates that the C–H bond cleavage transition state (**TS$_{BC_SpiroBpy}$**) and the resulting Ir(V) intermediate (**C$_{SpiroBpy}$**) are stabilized by donor–acceptor interactions between the π orbitals of the arene substrate (donor) and the C–H σ* orbital of the ligand backbone (acceptor: BD*(1) C31–H36). When the substrate is 1,3-$(Me_2N)_2$benzene, there is also an additional stabilization by the interaction between the lone pair of the nitrogen atom of the arene (LP(1) N123) and the C–H σ* orbital (BD*(1) C31–H36) in **TS$_{BC_SpiroBpy}$**. These CH–π interactions were found to be more significant for more electron-rich arenes (1,3-$(Me_2N)_2$benzene, 1,3-Me_2benzene > benzene), which is consistent with the experimental observation that the larger acceleration effect by **SpiroBpy** was observed when electron-rich substrates were used.

Supplementary Table 2. NBO analysis of **TS$_{BC_SpiroBpy}$** and **C$_{SpiroBpy}$** at the B3LYP-D3/SDD:6-31+G(d,p) level of theory.

	Donor NBOs	Acceptor NBOs	E(2) kcal/mol	
TS$_{BC_SpiroBpy}$ (1,3-$(Me_2N)_2$benzene)	94. LP (1) N123	297. BD*(1) C 31- H 36	0.41	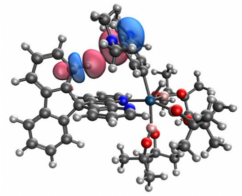
	219. BD (2) C107- C108	297. BD*(1) C 31- H 36	0.58	
	↑ π orbitals of the arene substrate ↓	↑ C–H σ* orbital of the ligand backbone ↓		
C$_{SpiroBpy}$ (1,3-$(Me_2N)_2$benzene)	212. BD (2) C104- C105	298. BD*(1) C 31- H 36	0.09	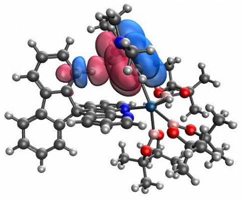
	217. BD (2) C106- C107	298. BD*(1) C 31- H 36	0.70	
	222. BD (2) C108- C109	298. BD*(1) C 31- H 36	0.50	
TS$_{BC_SpiroBpy}$ (1,3-Me_2benzene)	213. BD (2) C107- C108	282. BD*(1) C 31- H 36	1.18	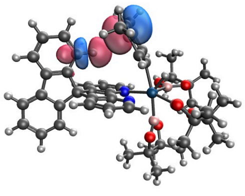
	↑ π orbitals of the arene substrate ↓	↑ C–H σ* orbital of the ligand backbone ↓		
C$_{SpiroBpy}$ (1,3-Me_2benzene)	206. BD (2) C104- C105	282. BD*(1) C 31- H 36	0.12	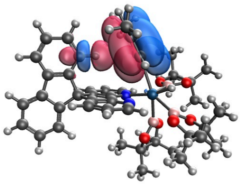
	211. BD (2) C106- C107	282. BD*(1) C 31- H 36	0.48	
	216. BD (2) C108- C109	282. BD*(1) C 31- H 36	0.46	
TS$_{BC_SpiroBpy}$ (benzene)	204. BD (1) C104- H110	273. BD*(1) C 31- H 36	0.08	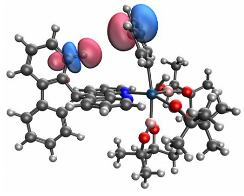
	211. BD (2) C107- C108	273. BD*(1) C 31- H 36	0.09	
	↑ π orbitals of the arene substrate ↓	↑ C–H σ* orbital of the ligand backbone ↓		
C$_{SpiroBpy}$ (benzene)	204. BD (2) C104- C105	274. BD*(1) C 31- H 36	0.05	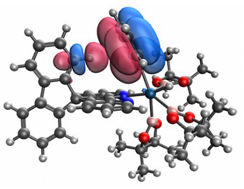
	209. BD (2) C106- C107	274. BD*(1) C 31- H 36	0.44	
	214. BD (2) C108- C109	274. BD*(1) C 31- H 36	0.27	

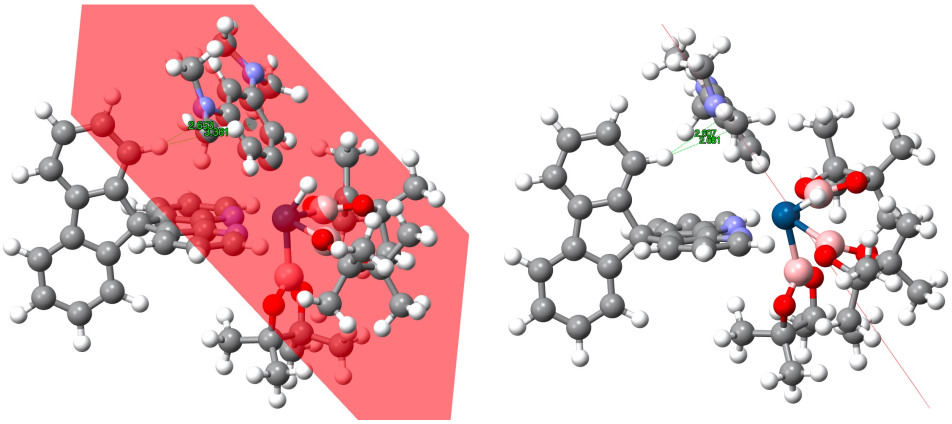

$TS_{BC\_SpiroBpy}$ $C_{SpiroBpy}$

Supplementary Figure 7. CH–π distance. a) distance between the H atom and the plane of the arene substrate: 2.653 Å for $\mathbf{TS_{BC\_SpiroBpy}}$ and 2.607 Å for $\mathbf{C_{SpiroBpy}}$. b) distance between the H atom and the centroid of the benzene ring of the arene substrate: 3.361 Å for $\mathbf{TS_{BC\_SpiroBpy}}$ and 2.681 Å for $\mathbf{C_{SpiroBpy}}$.

Energies of Stationary Points

Supplementary Table 3. Electronic Energies (E), Zero-point Energy Corrected Energies (E + ZPE), Electronic and Thermal Enthalpies (H), Gibbs Free Energies (G) of Stationary Points (in hartrees) Calculated at the M06/SDD:6-311+G(d,p)$_{THF(SMD)}$//B3LYP-D3/SDD:6-31+G(d,p) level of theory.

	E	*E+ZPE*	*H*	*G*
1,3-($Me_2N)_2$benzene	-500.3582769	-500.1118699	-500.0980319	-500.1509589
$\mathbf{A_{L1}}$	-1872.445542	-1871.73401	-1871.690675	-1871.810213
$\mathbf{B_{L1}}$	-2372.829779	-2371.869791	-2371.812145	-2371.961013
$\mathbf{TS_{L1}}$	-2372.776388	-2371.821318	-2371.763964	-2371.912786
$\mathbf{C_{L1}}$	-2372.794669	-2371.83741	-2371.78007	-2371.927615
$\mathbf{A_{spirobpy}}$	-2333.496902	-2332.645353	-2332.593675	-2332.730959
$\mathbf{B_{spirobpy}}$	-2833.881854	-2832.781774	-2832.715838	-2832.881781
$\mathbf{TS_{spirobpy}}$	-2833.829802	-2832.734936	-2832.669151	-2832.836868
$\mathbf{C_{spirobpy}}$	-2833.847393	-2832.75034	-2832.684565	-2832.850506
$\mathbf{A_{tmphen}}$	-2067.909507	-2067.080327	-2067.030014	-2067.163497
$\mathbf{B_{tmphen}}$	-2568.29827	-2567.220138	-2567.155838	-2567.317941
$\mathbf{TS_{tmphen}}$	-2568.24132	-2567.168539	-2567.104366	-2567.267126
$\mathbf{C_{tmphen}}$	-2568.260551	-2567.185849	-2567.121585	-2567.283662

10. NMR charts

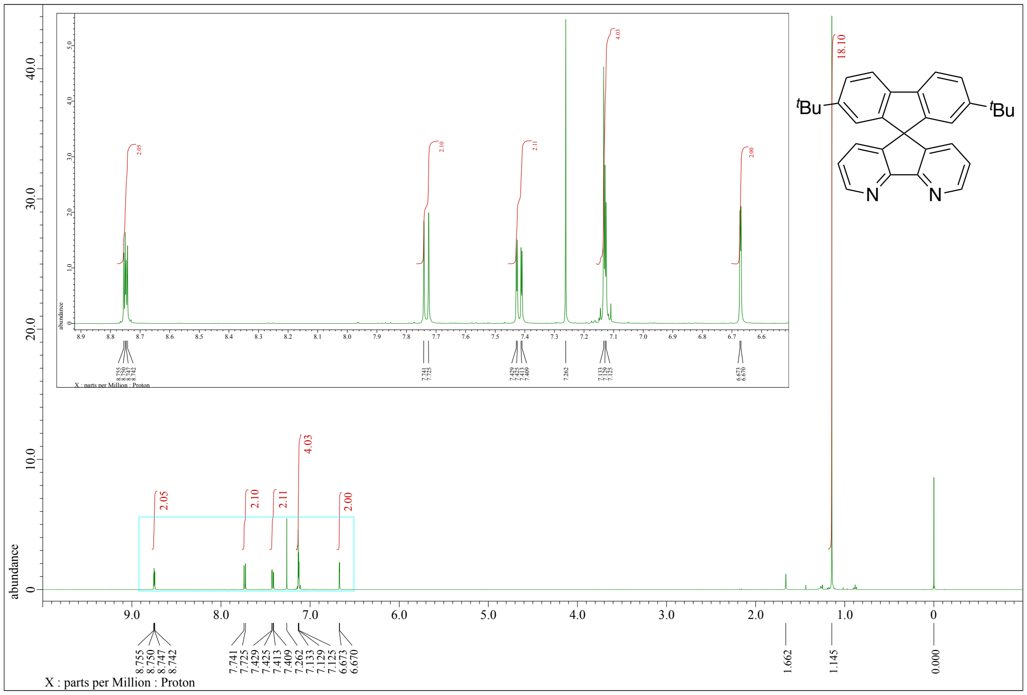

Supplementary Figure 8. ^{1}H NMR spectrum of ***t*Bu-SpiroBpy** (500 MHz, $CDCl_3$)

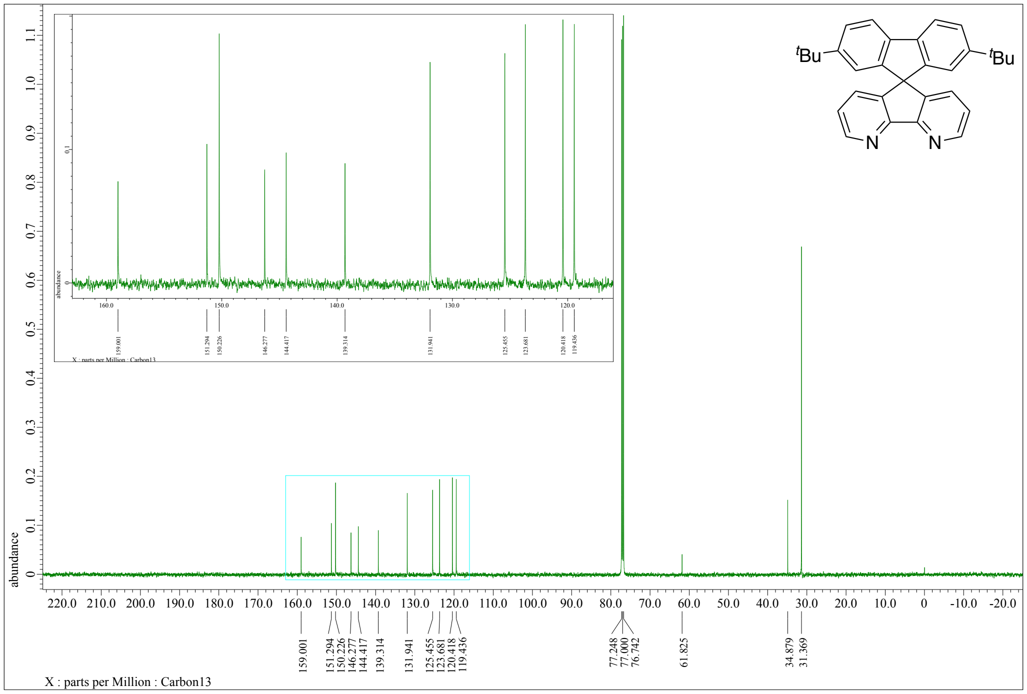

Supplementary Figure 9. ^{13}C NMR spectrum of ***t*Bu-SpiroBpy** (125 MHz, $CDCl_3$)

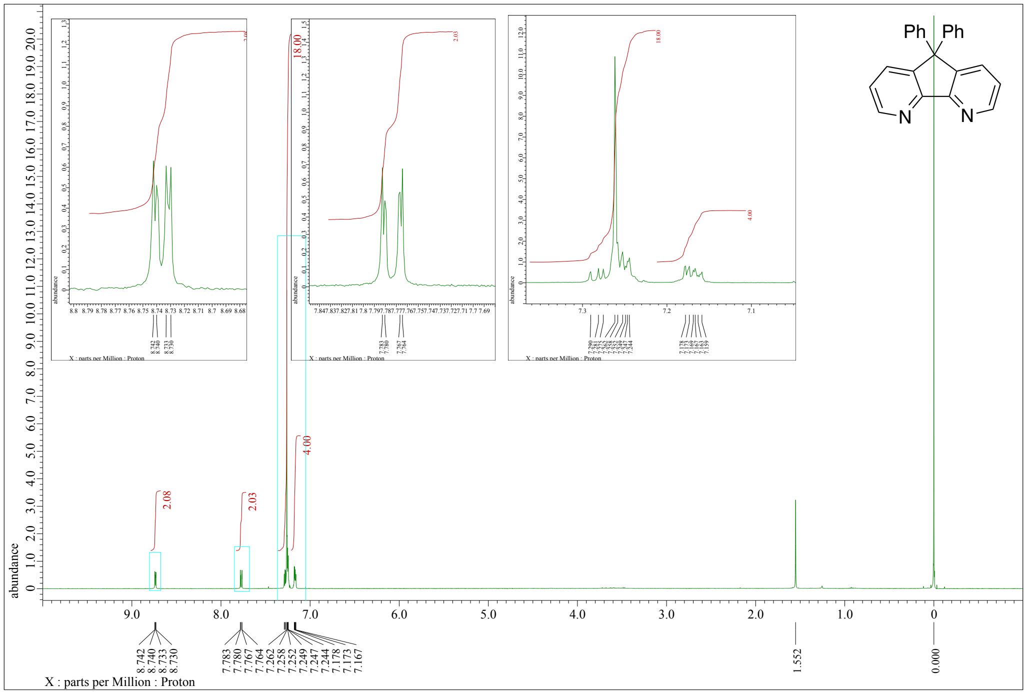

Supplementary Figure 10. ^{1}H NMR spectrum of **L2** (500 MHz, $CDCl_3$)

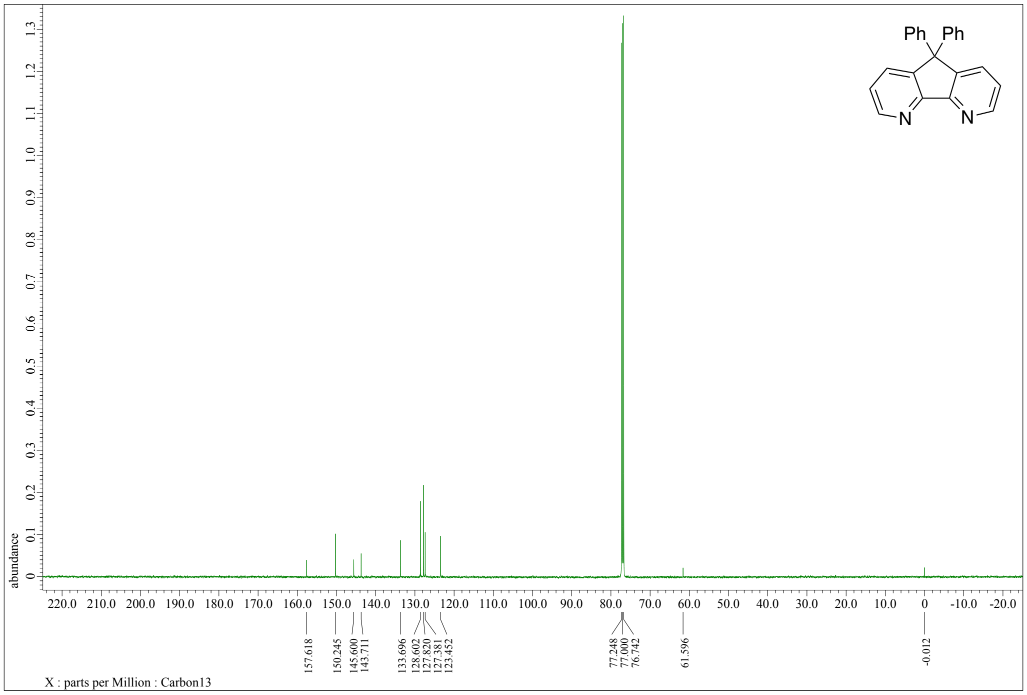

Supplementary Figure 11. ^{13}C NMR spectrum of **L2** (125 MHz, $CDCl_3$)

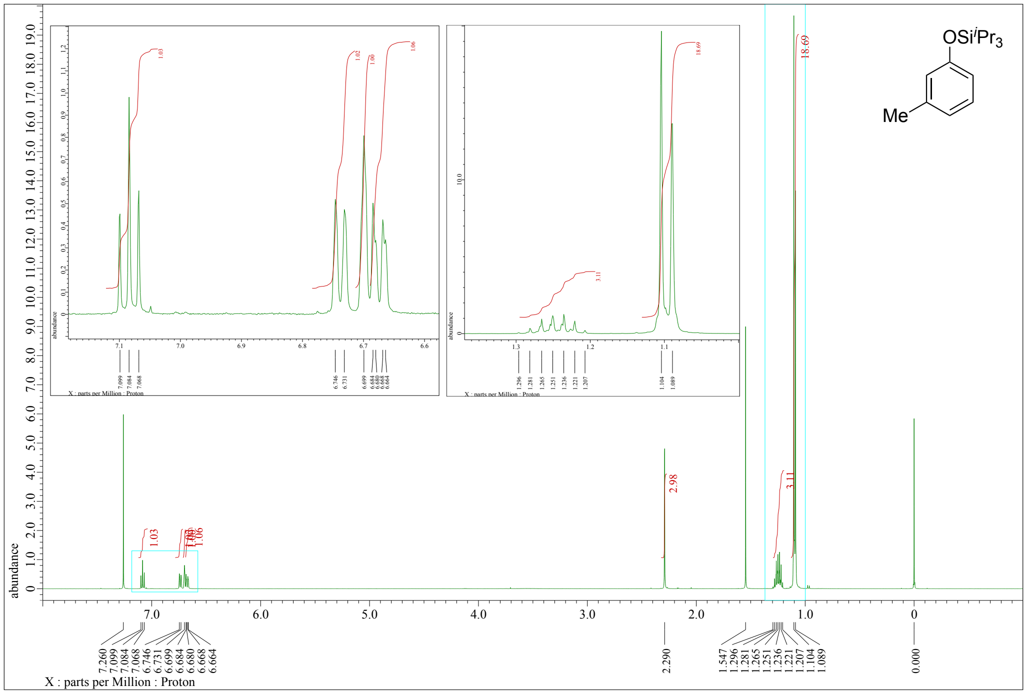

Supplementary Figure 12. ^{1}H NMR spectrum of **1e** (500 MHz, $CDCl_3$)

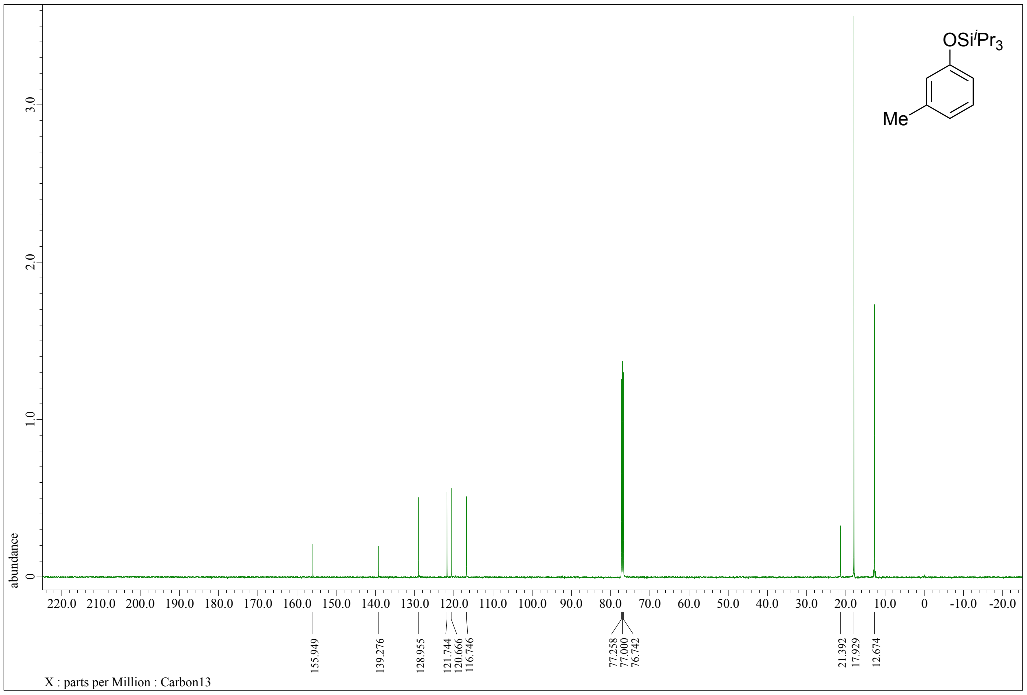

Supplementary Figure 13. ^{13}C NMR spectrum of **1e** (125 MHz, $CDCl_3$)

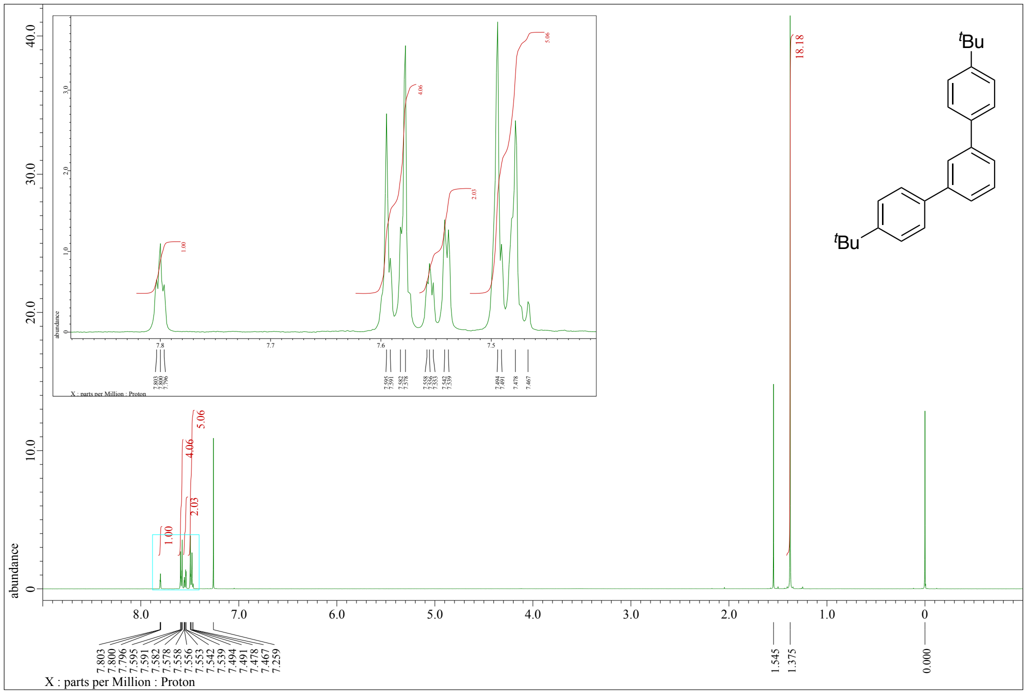

Supplementary Figure 14. ^{1}H NMR spectrum of **1f** (500 MHz, $CDCl_3$)

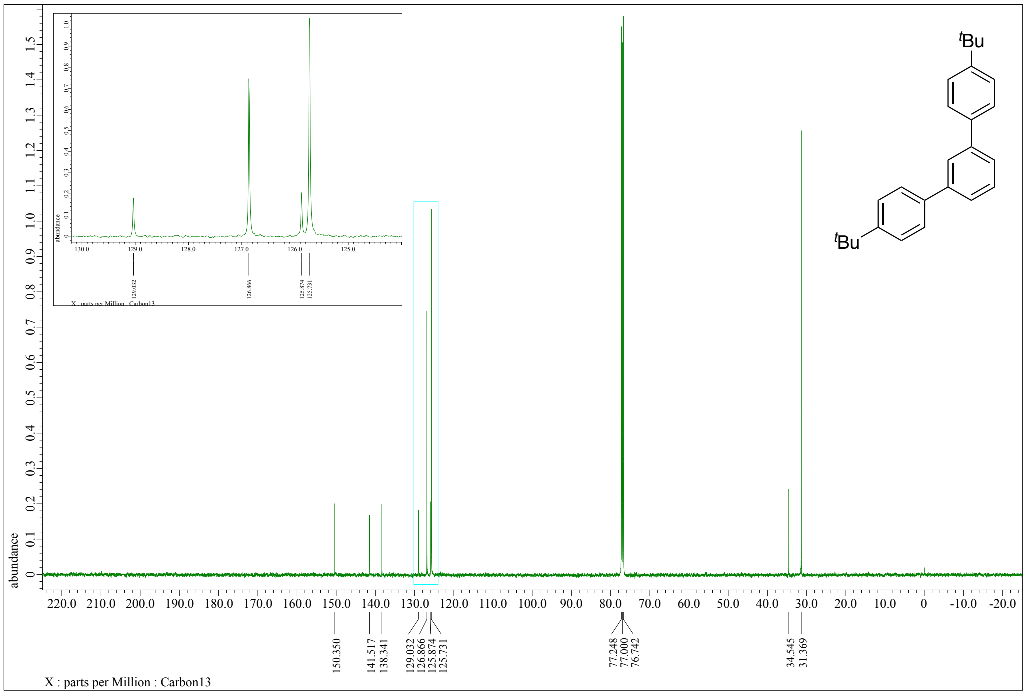

Supplementary Figure 15. ^{13}C NMR spectrum of **1f** (125 MHz, $CDCl_3$)

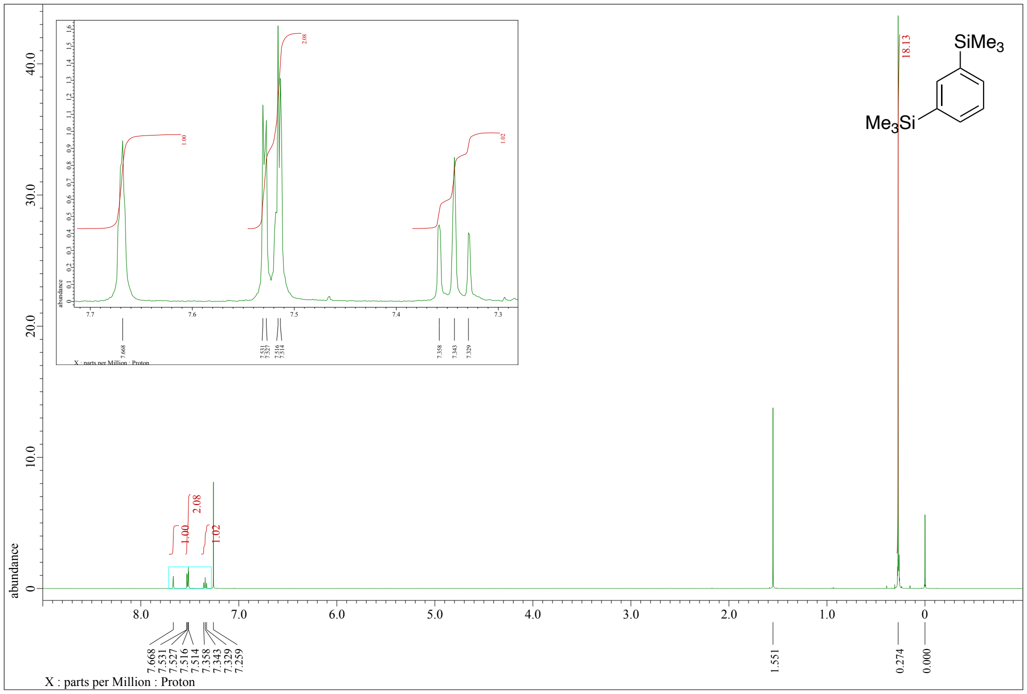

Supplementary Figure 16. ^{1}H NMR spectrum of **1k** (500 MHz, $CDCl_3$)

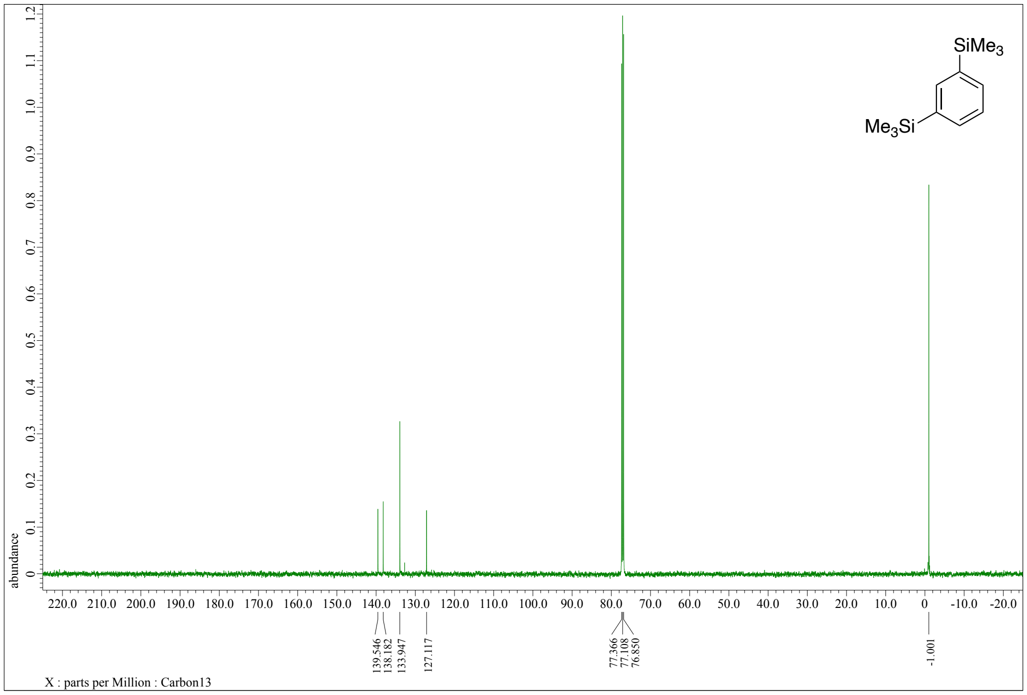

Supplementary Figure 17. ^{13}C NMR spectrum of **1k** (125 MHz, $CDCl_3$)

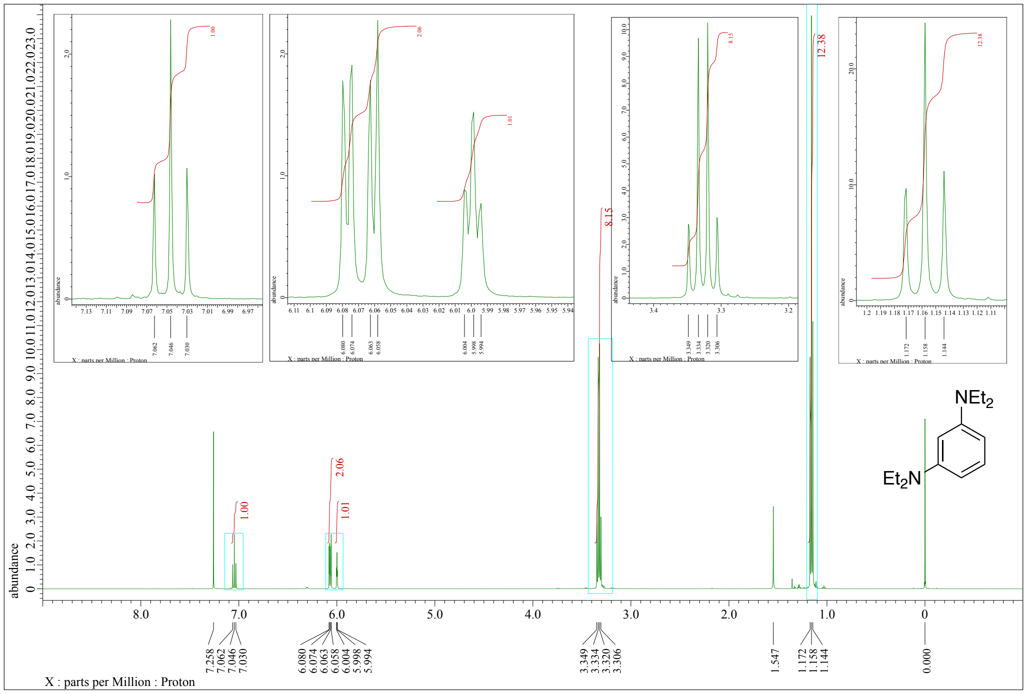

Supplementary Figure 18. ^{1}H NMR spectrum of **1m** (500 MHz, $CDCl_3$)

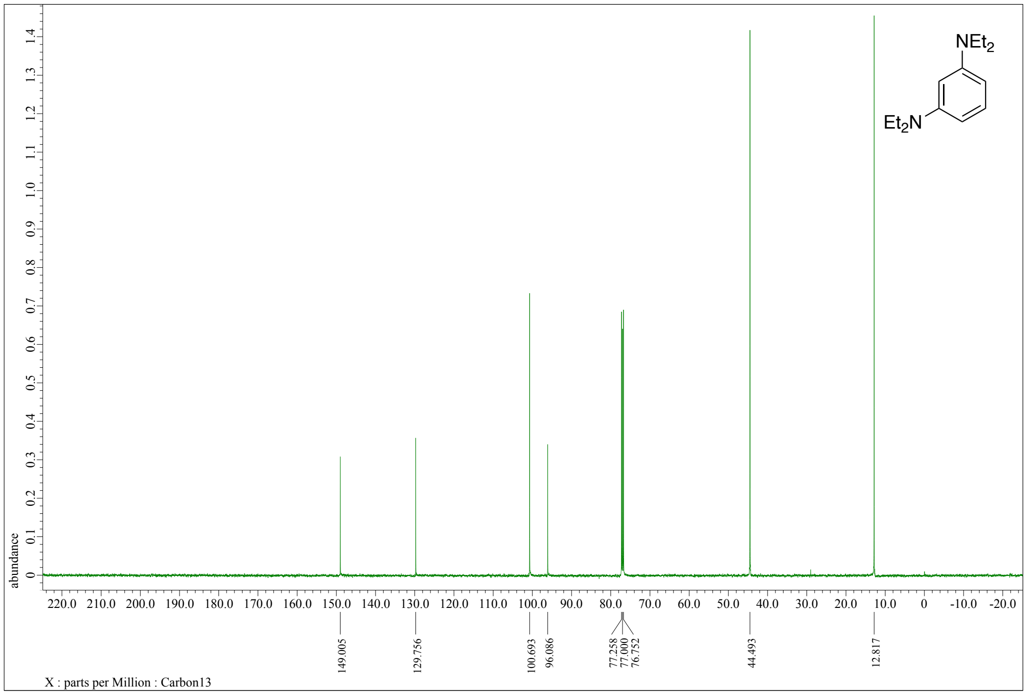

Supplementary Figure 19. ^{13}C NMR spectrum of **1m** (125 MHz, $CDCl_3$)

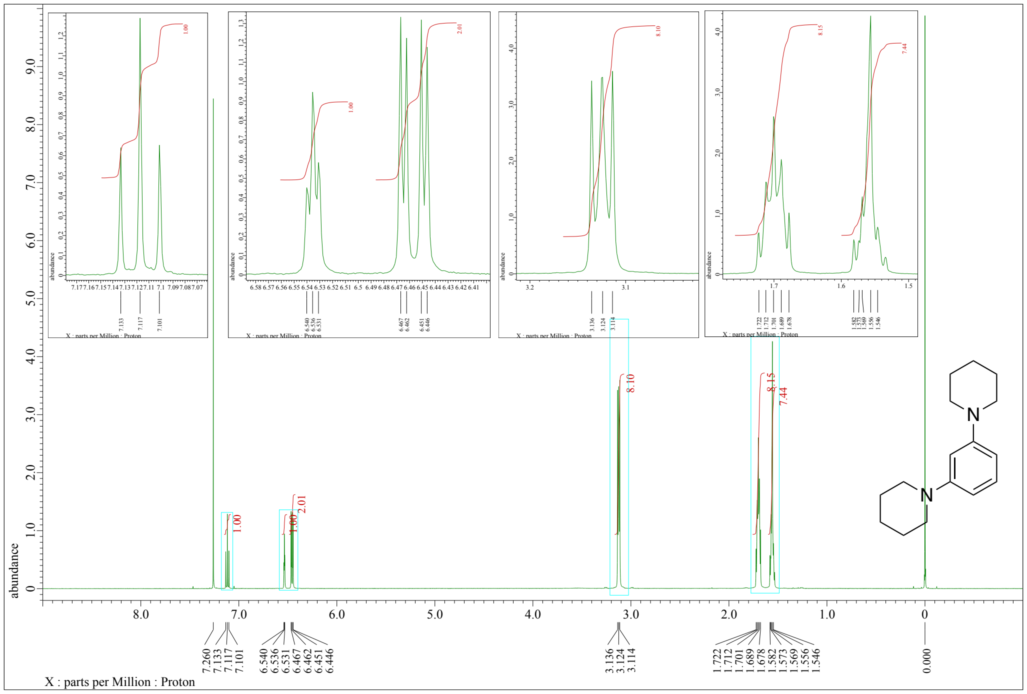

Supplementary Figure 20. ^{1}H NMR spectrum of **1n** (500 MHz, $CDCl_3$)

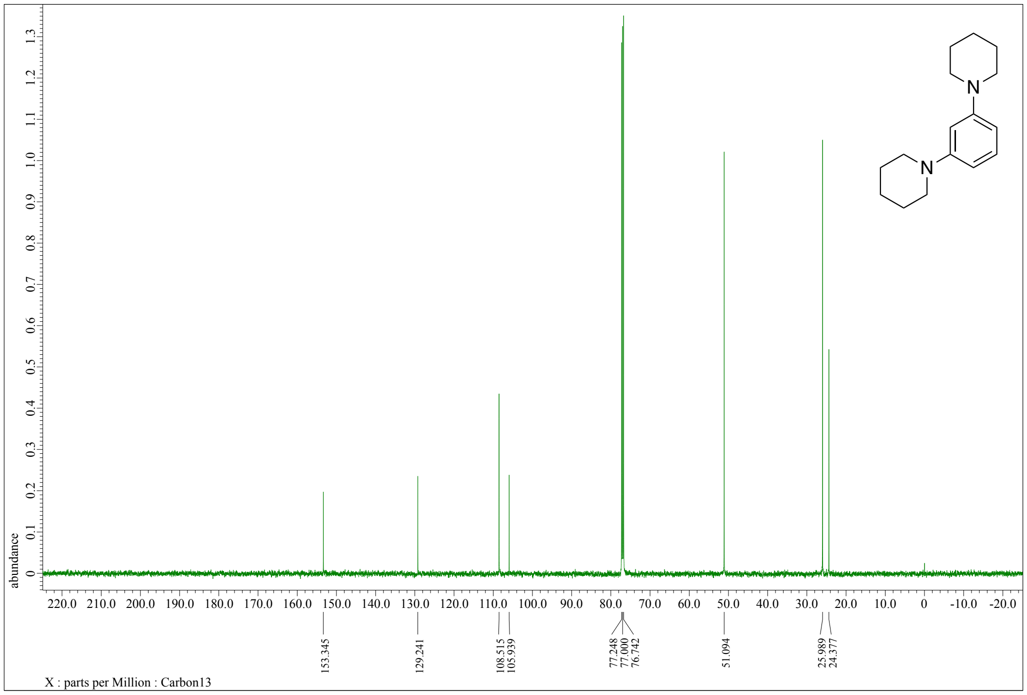

Supplementary Figure 21. ^{13}C NMR spectrum of **1n** (125 MHz, $CDCl_3$)

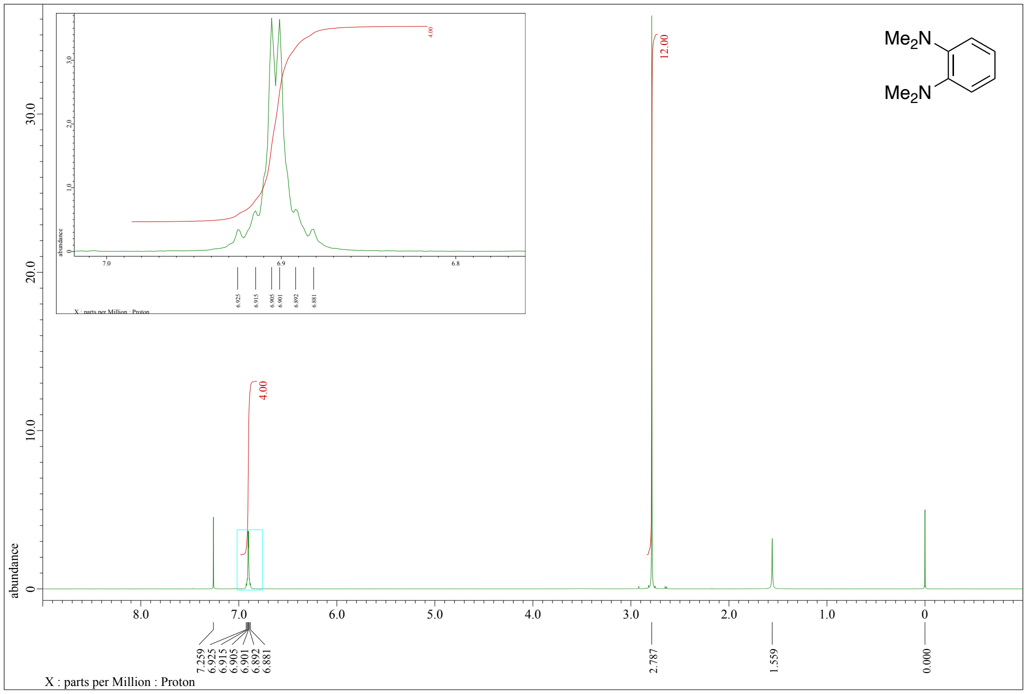

Supplementary Figure 22. ^{1}H NMR spectrum of **1o** (500 MHz, $CDCl_3$)

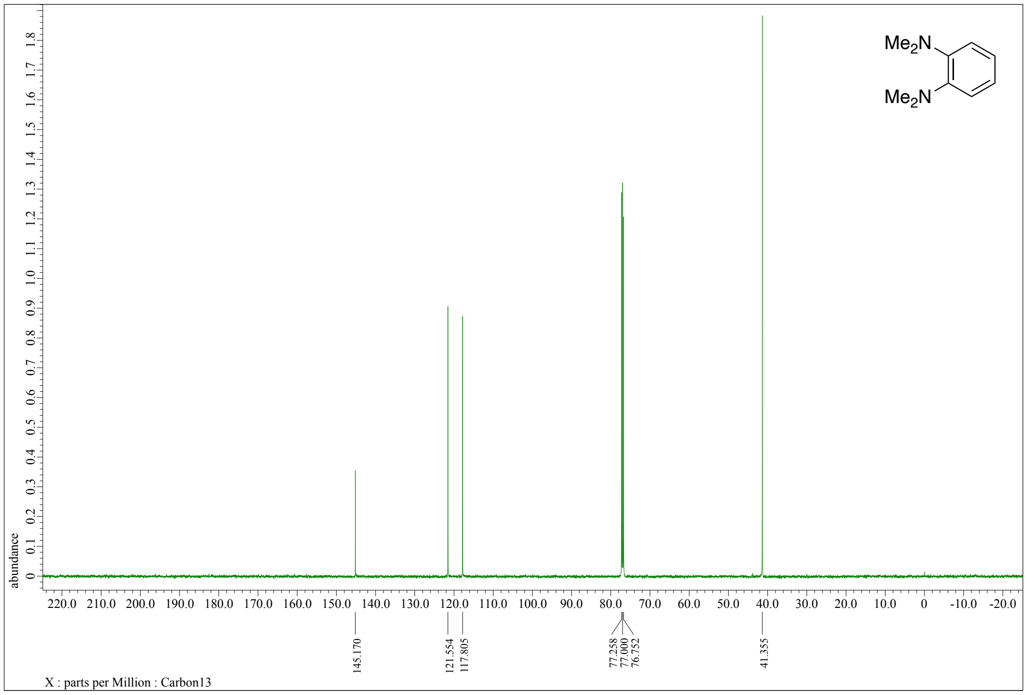

Supplementary Figure 23. ^{13}C NMR spectrum of **1o** (125 MHz, $CDCl_3$)

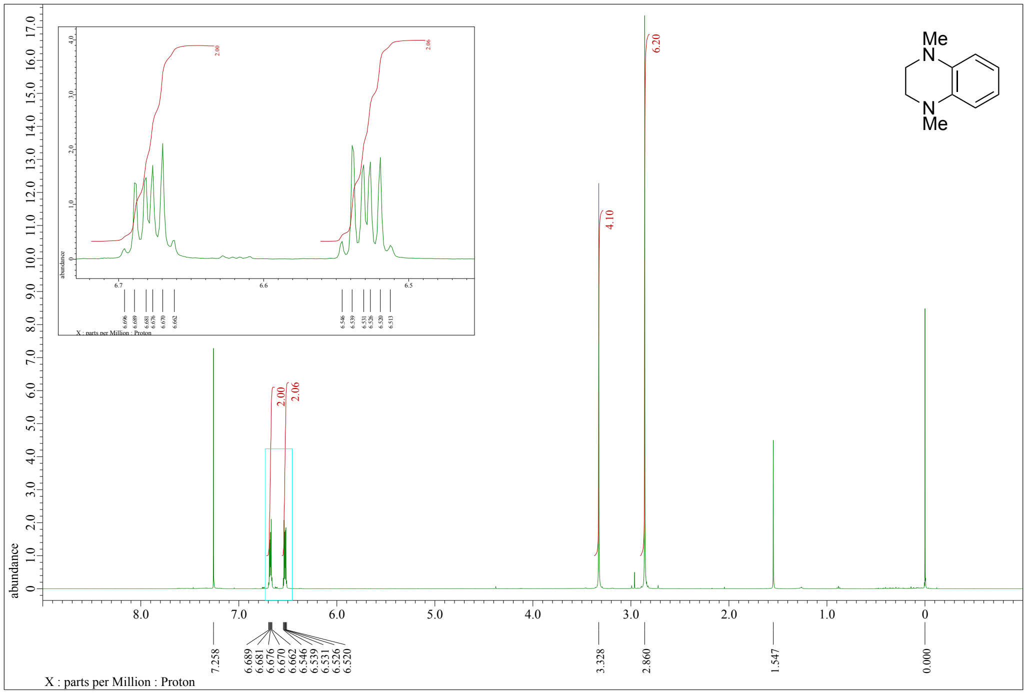

Supplementary Figure 24. ^{1}H NMR spectrum of **1p** (500 MHz, $CDCl_3$)

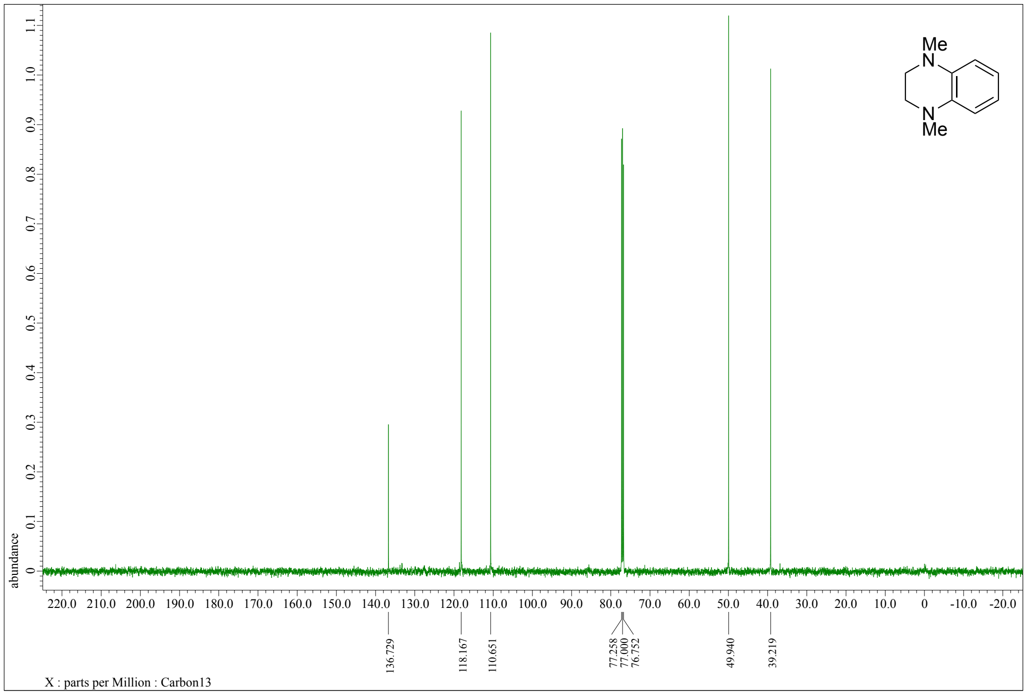

Supplementary Figure 25. ^{13}C NMR spectrum of **1p** (125 MHz, $CDCl_3$)

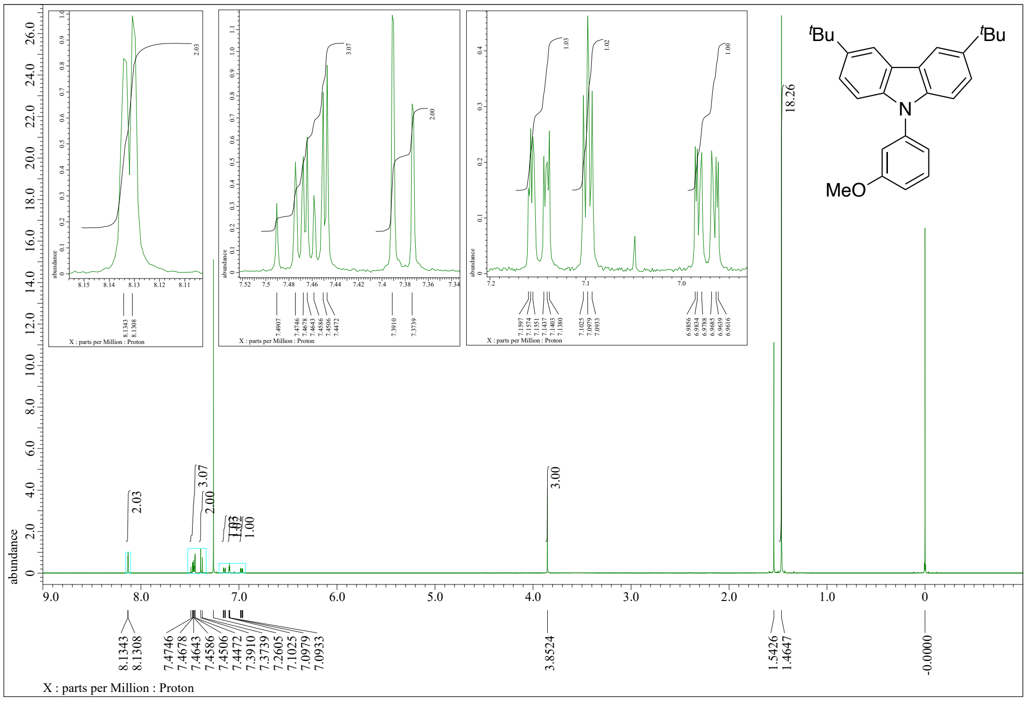

Supplementary Figure 26. ^{1}H NMR spectrum of **1q** (500 MHz, $CDCl_3$)

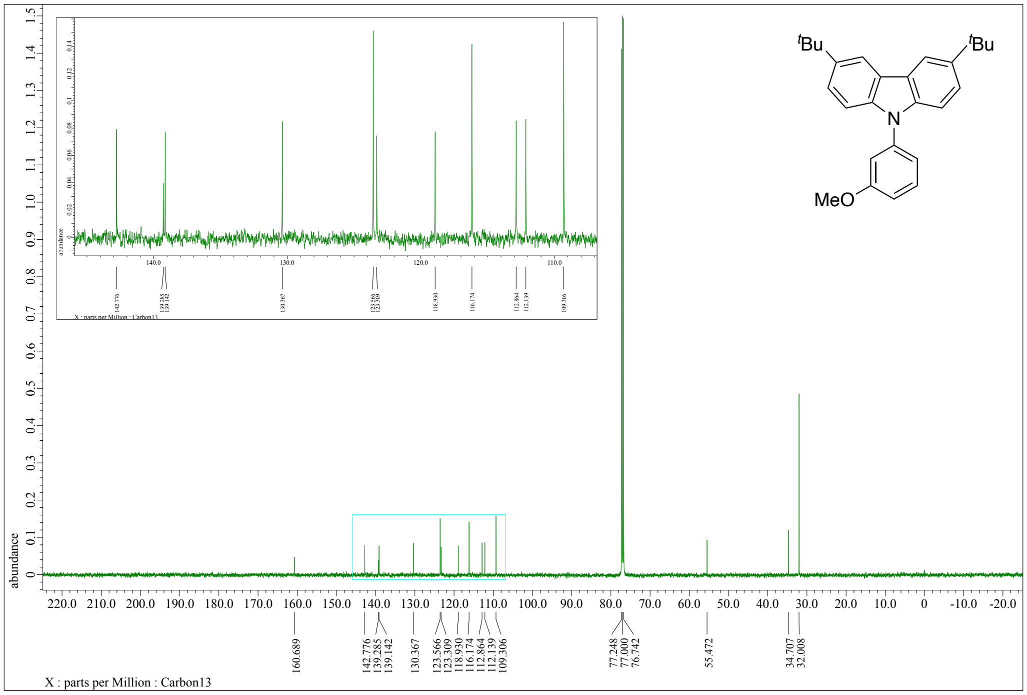

Supplementary Figure 27. ^{13}C NMR spectrum of **1q** (125 MHz, $CDCl_3$)

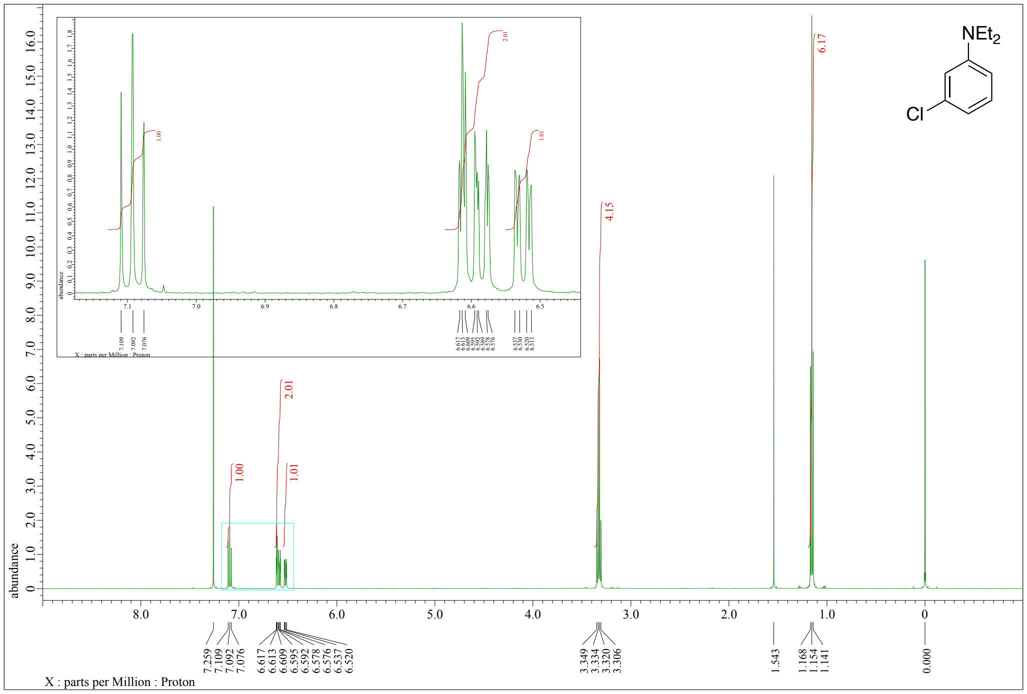

Supplementary Figure 28. ^{1}H NMR spectrum of **1v** (500 MHz, $CDCl_3$)

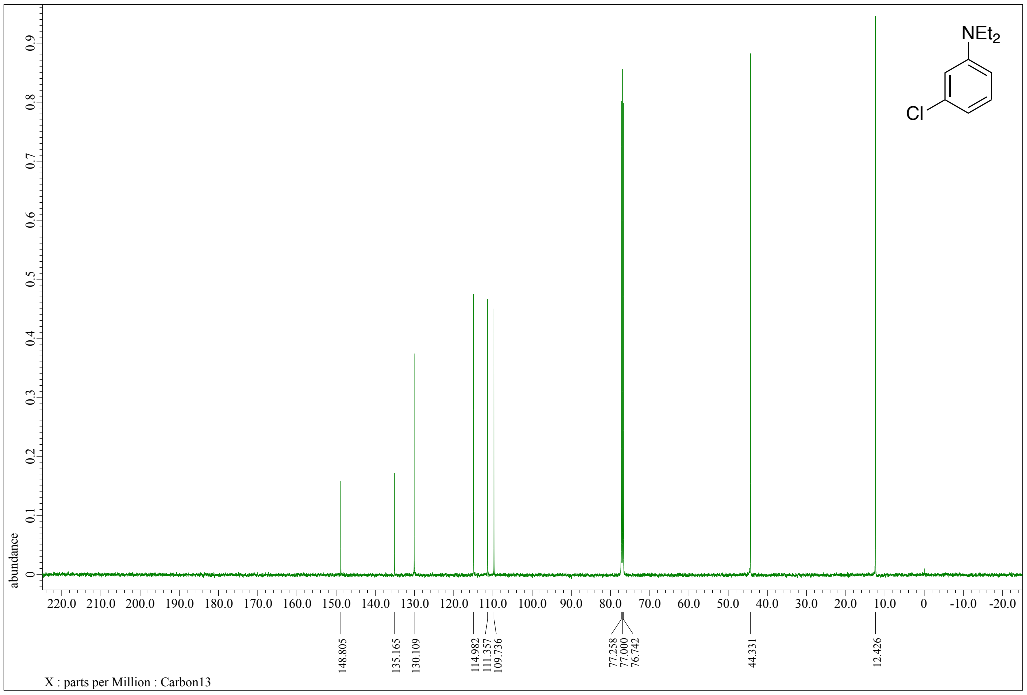

Supplementary Figure 29. ^{13}C NMR spectrum of **1v** (125 MHz, $CDCl_3$)

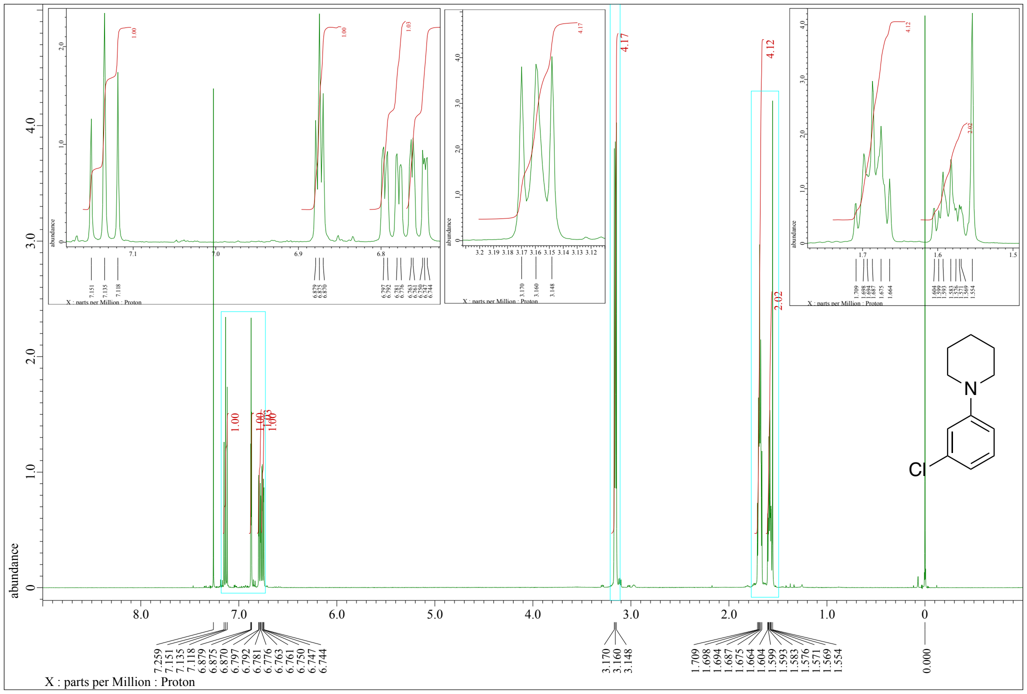

Supplementary Figure 30. ^{1}H NMR spectrum of **1w** (500 MHz, $CDCl_3$)

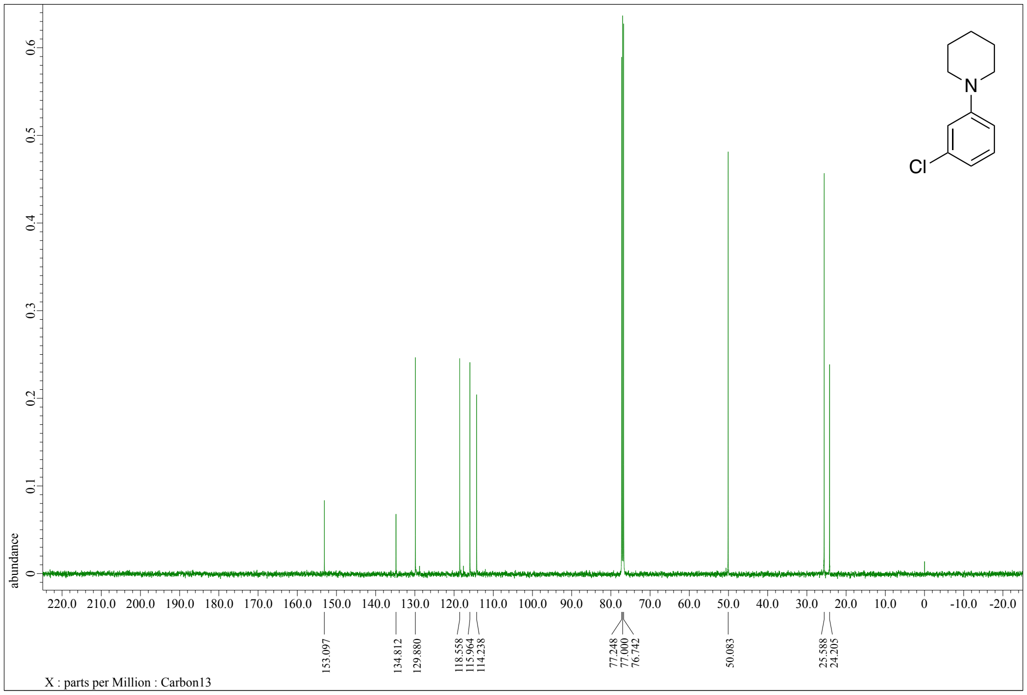

Supplementary Figure 31. ^{13}C NMR spectrum of **1w** (125 MHz, $CDCl_3$)

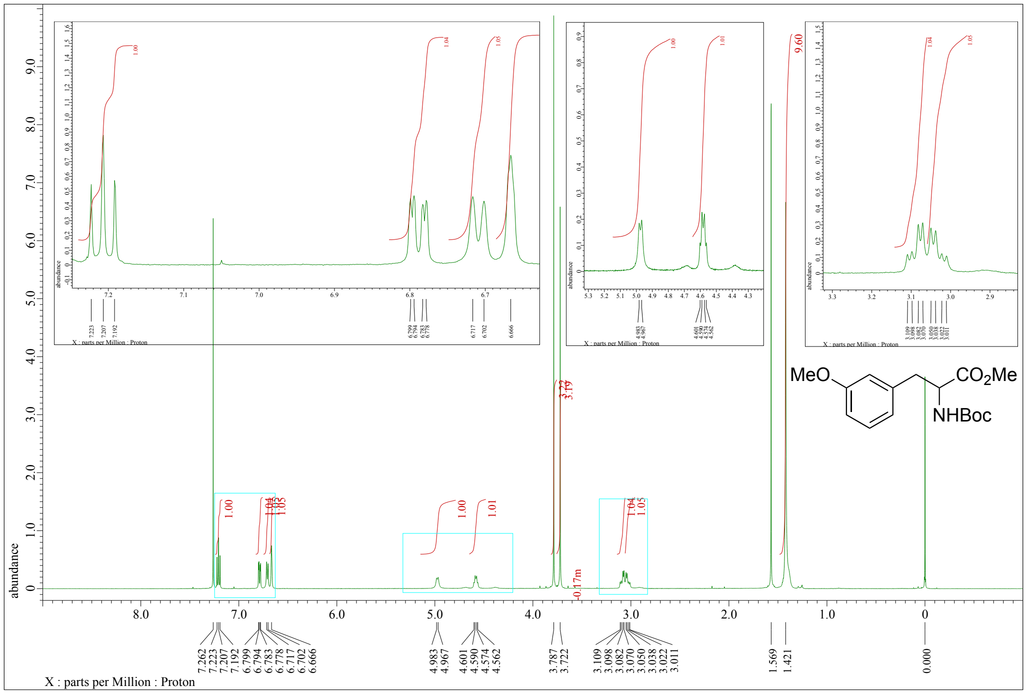

Supplementary Figure 32. ^{1}H NMR spectrum of **3b** (500 MHz, $CDCl_3$)

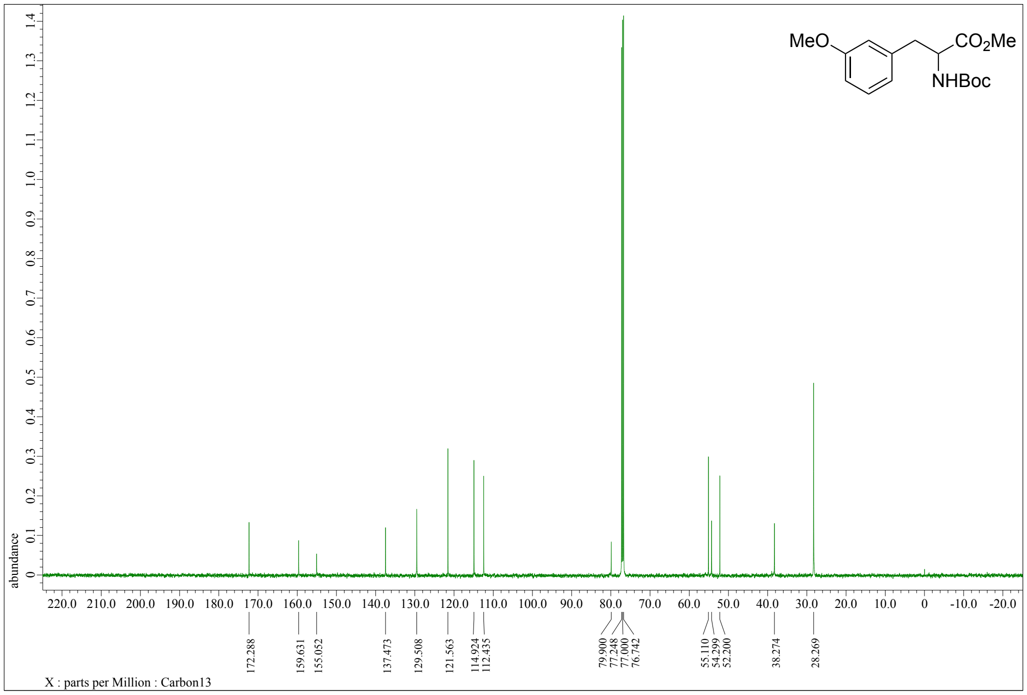

Supplementary Figure 33. ^{13}C NMR spectrum of **3b** (125 MHz, $CDCl_3$)

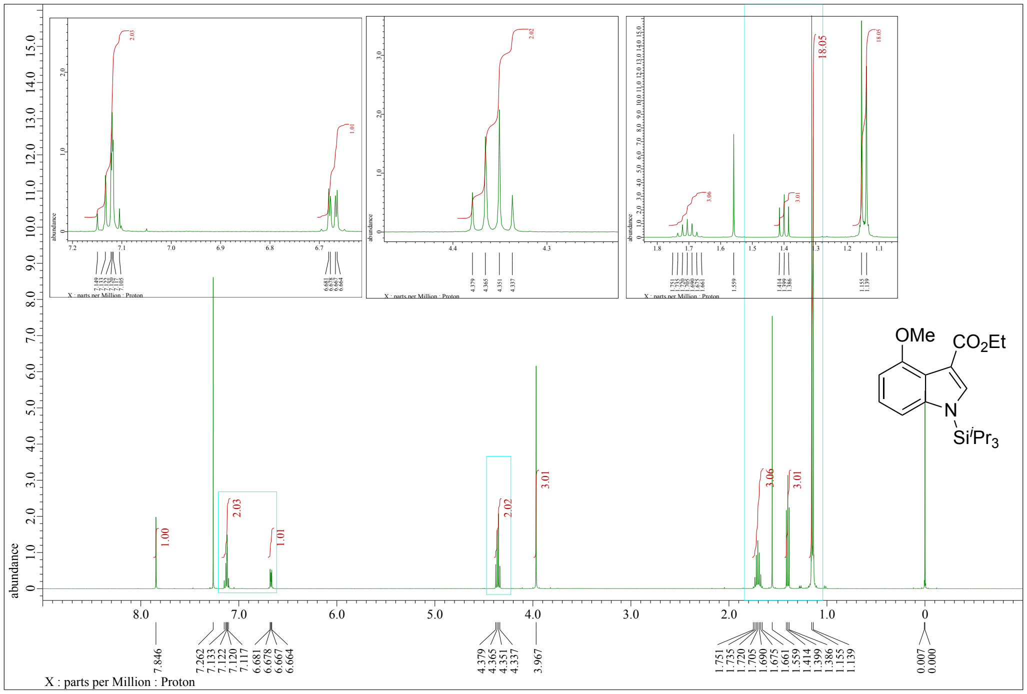

Supplementary Figure 34. ^{1}H NMR spectrum of **3c** (500 MHz, $CDCl_3$)

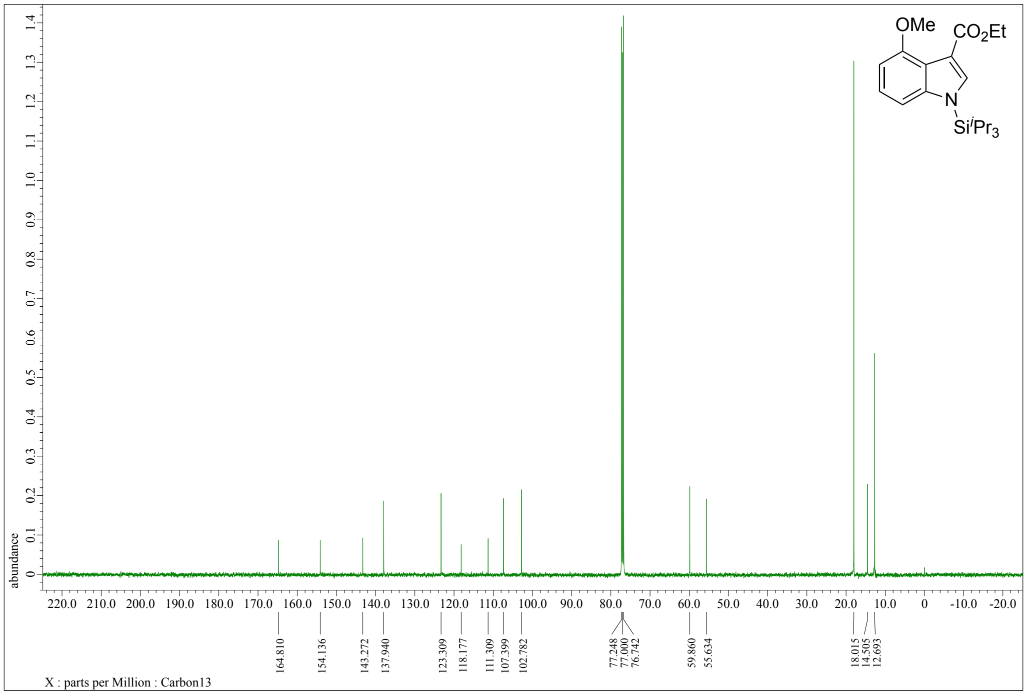

Supplementary Figure 35. ^{13}C NMR spectrum of **3c** (125 MHz, $CDCl_3$)

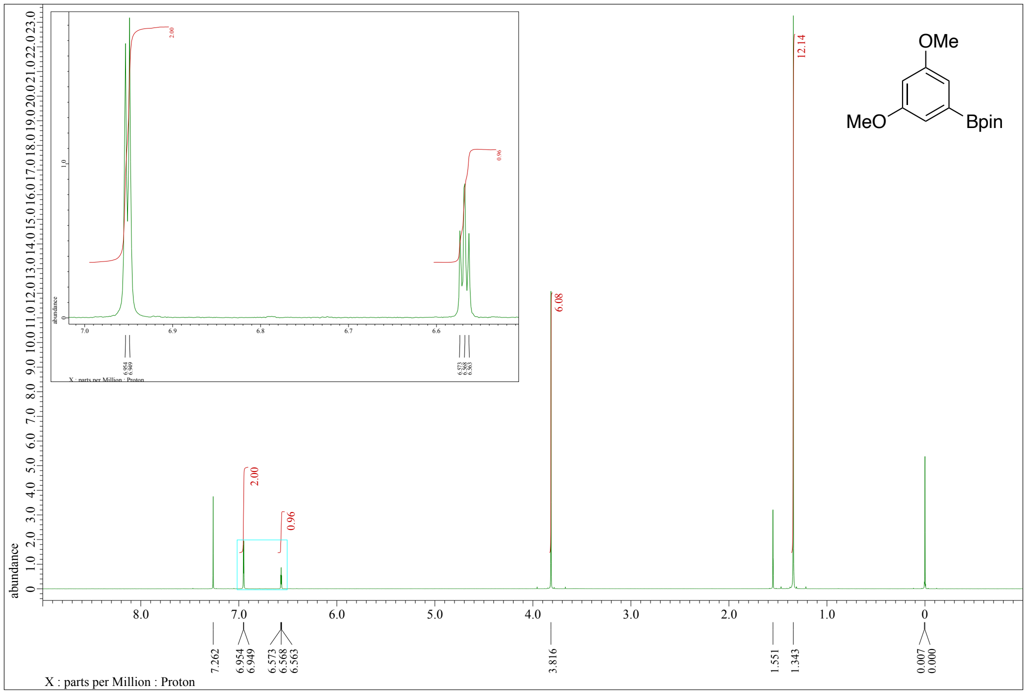

Supplementary Figure 36. ^{1}H NMR spectrum of **2a** (500 MHz, $CDCl_3$)

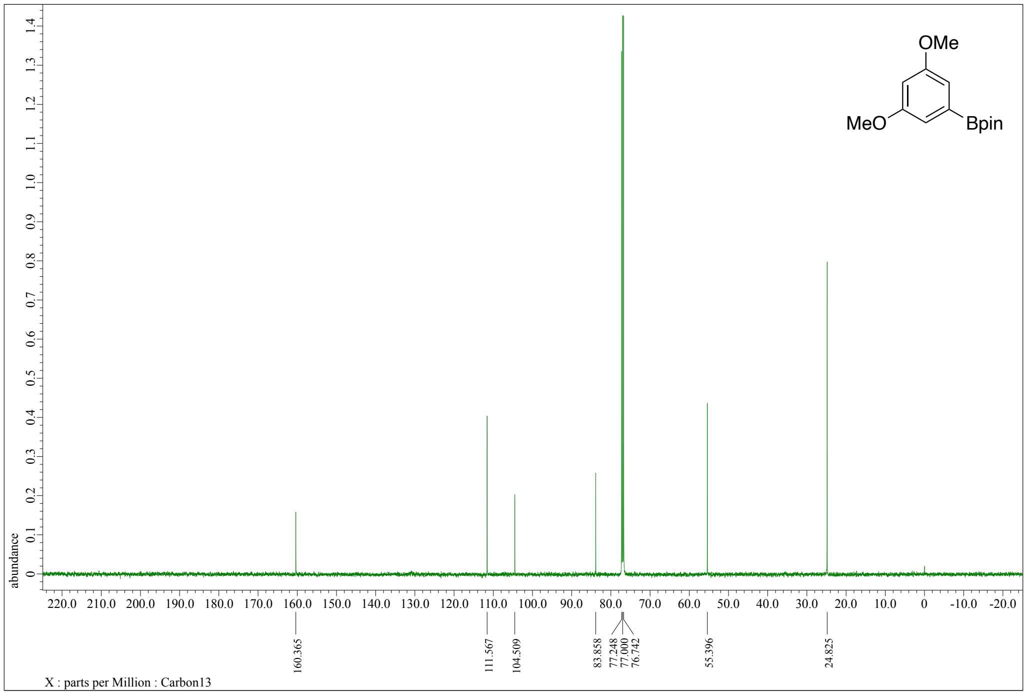

Supplementary Figure 37. ^{13}C NMR spectrum of **2a** (125 MHz, $CDCl_3$)

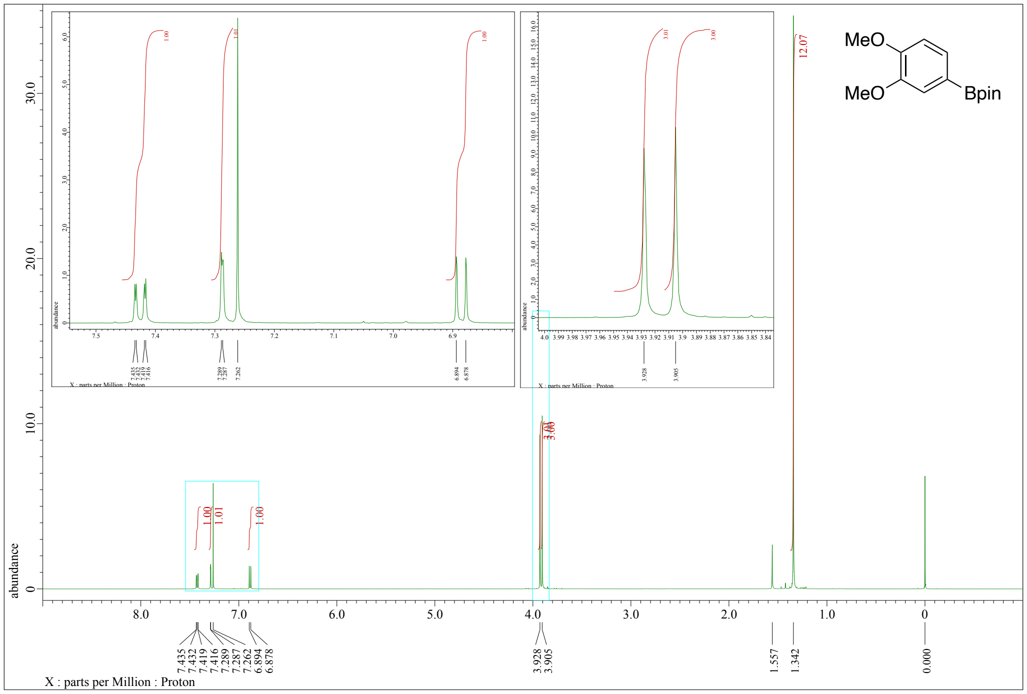

Supplementary Figure 38. ^{1}H NMR spectrum of **2b** (500 MHz, $CDCl_3$)

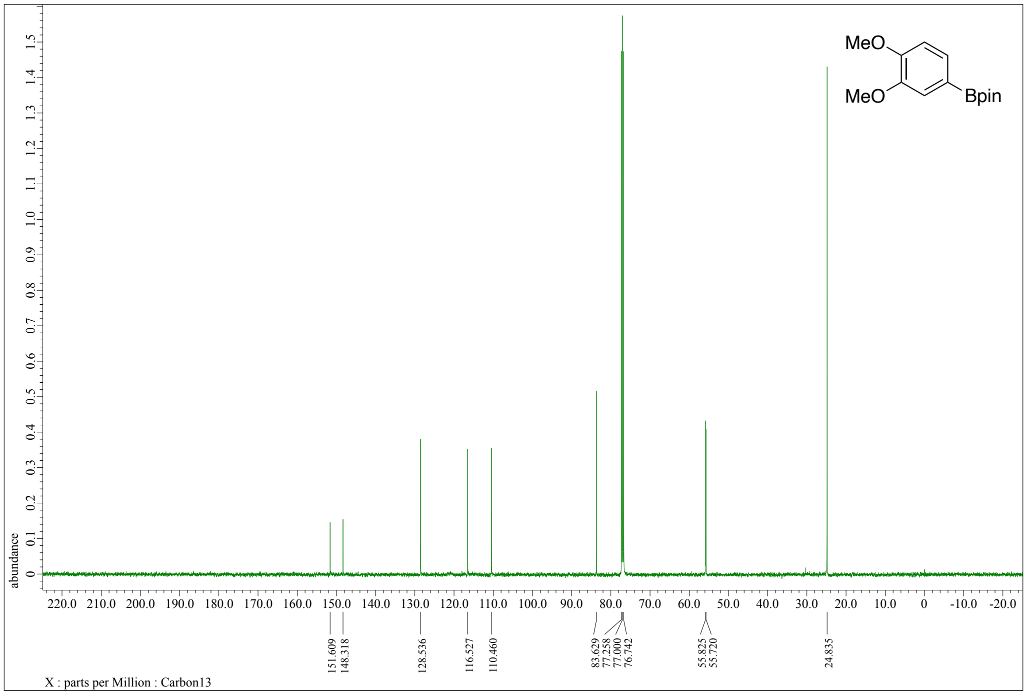

Supplementary Figure 39. ^{13}C NMR spectrum of **2b** (125 MHz, $CDCl_3$)

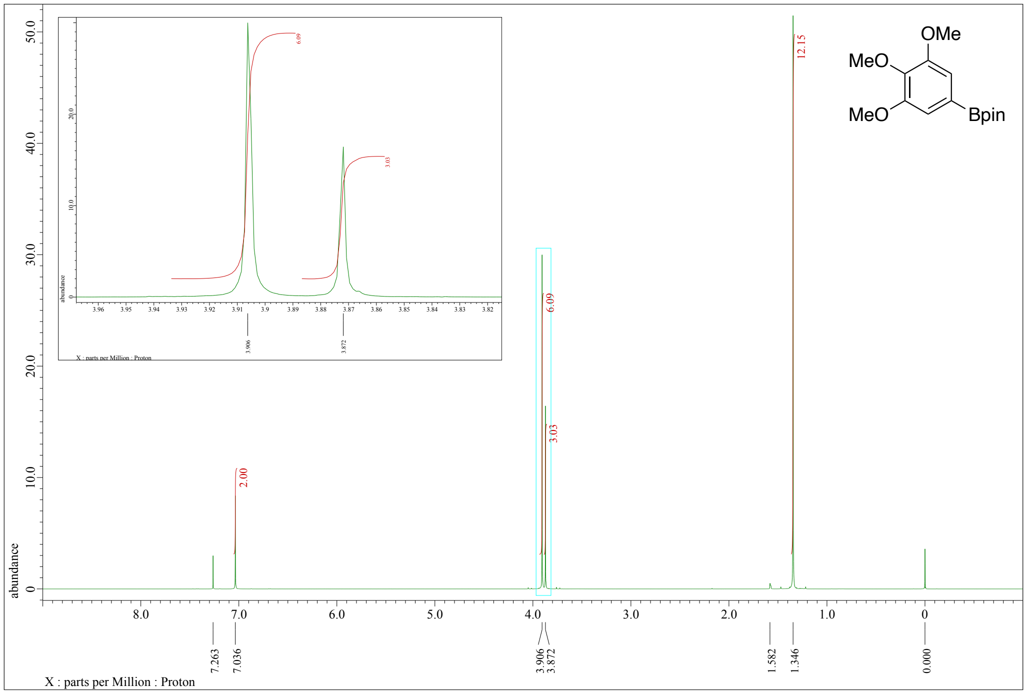

Supplementary Figure 40. ^{1}H NMR spectrum of **2c** (500 MHz, $CDCl_3$)

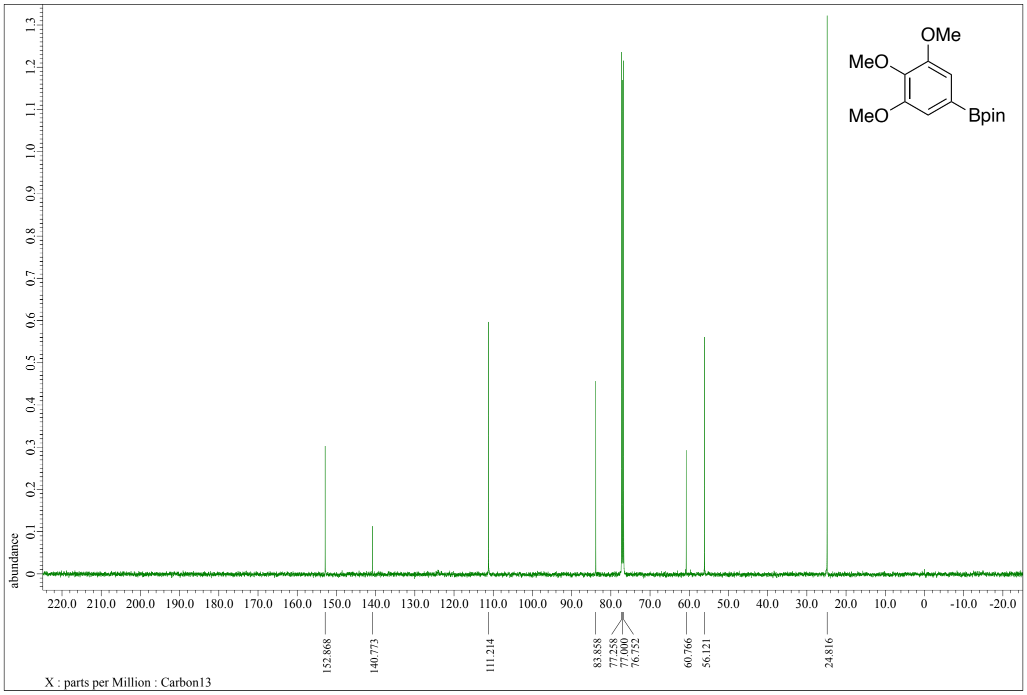

Supplementary Figure 41. ^{13}C NMR spectrum of **2c** (125 MHz, $CDCl_3$)

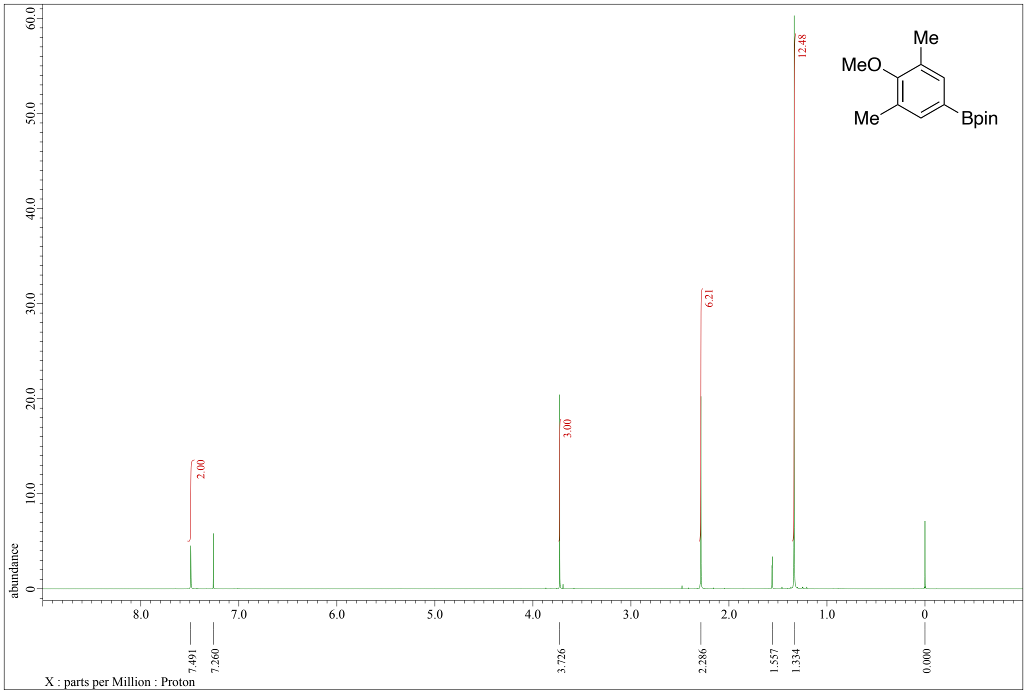

Supplementary Figure 42. ^{1}H NMR spectrum of **2d** (500 MHz, $CDCl_3$)

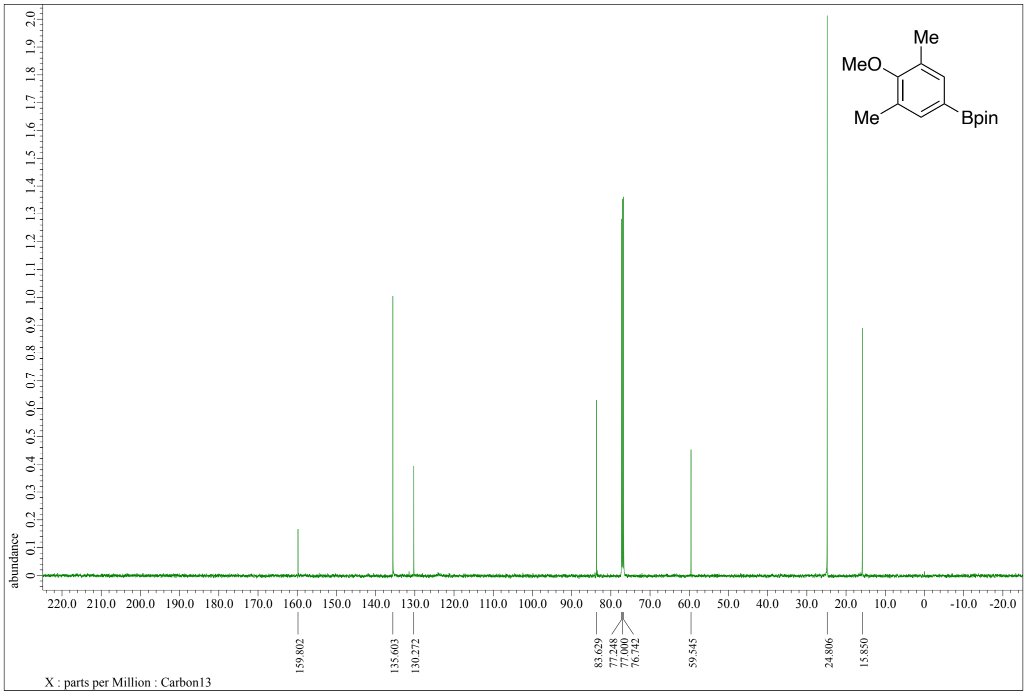

Supplementary Figure 43. ^{13}C NMR spectrum of **2d** (125 MHz, $CDCl_3$)

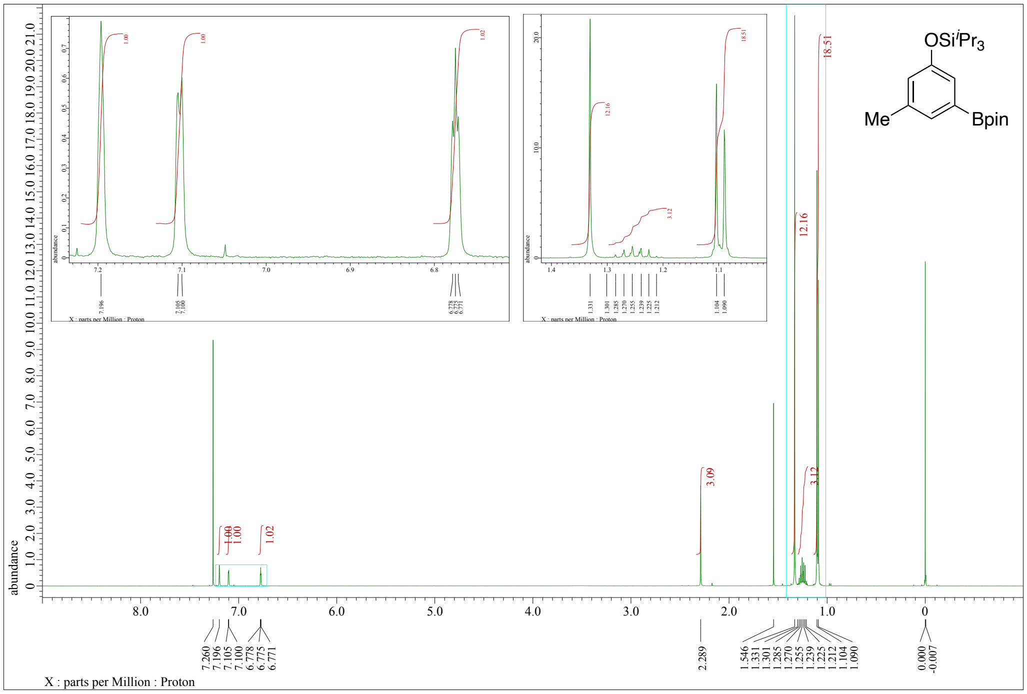

Supplementary Figure 44. ^{1}H NMR spectrum of **2e** (500 MHz, $CDCl_3$)

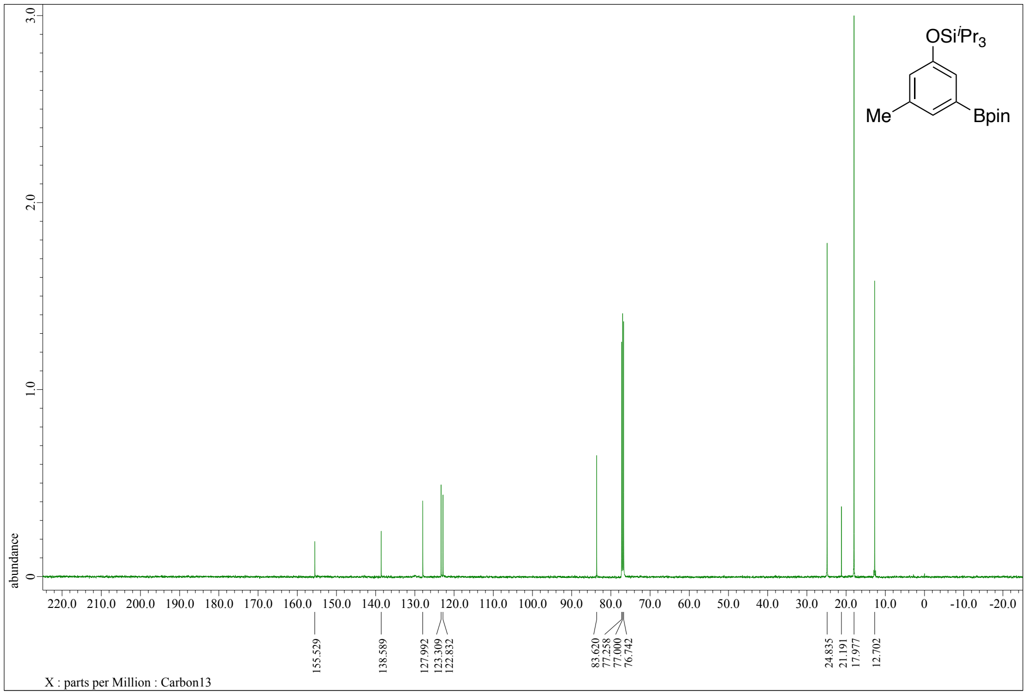

Supplementary Figure 45. ^{13}C NMR spectrum of **2e** (125 MHz, $CDCl_3$)

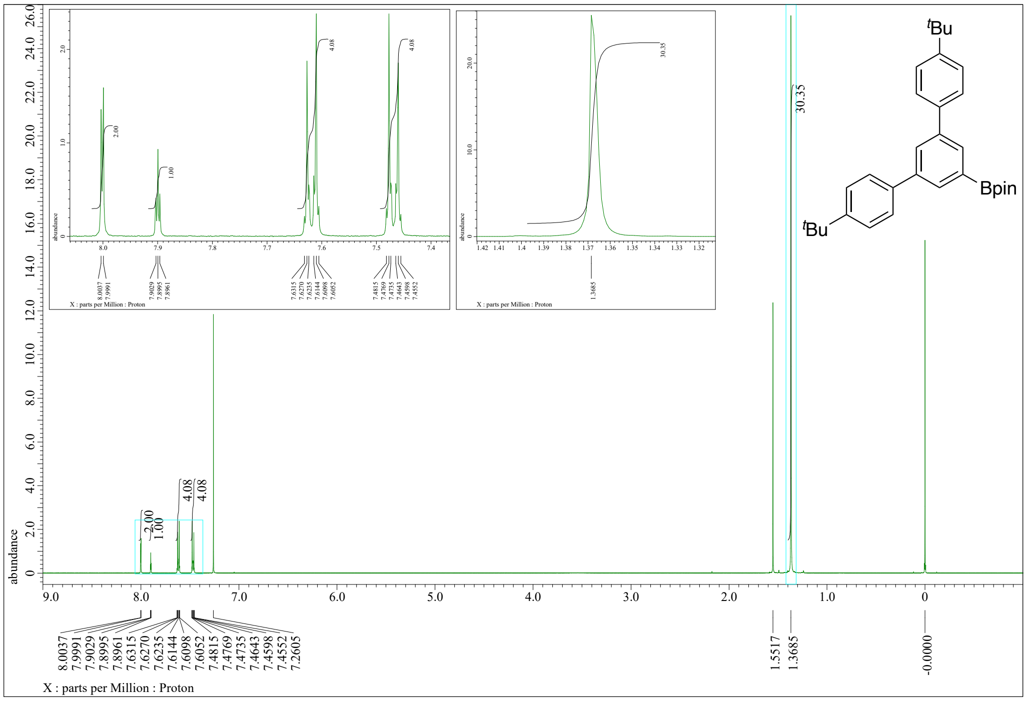

Supplementary Figure 46. ^{1}H NMR spectrum of **2f** (500 MHz, $CDCl_3$)

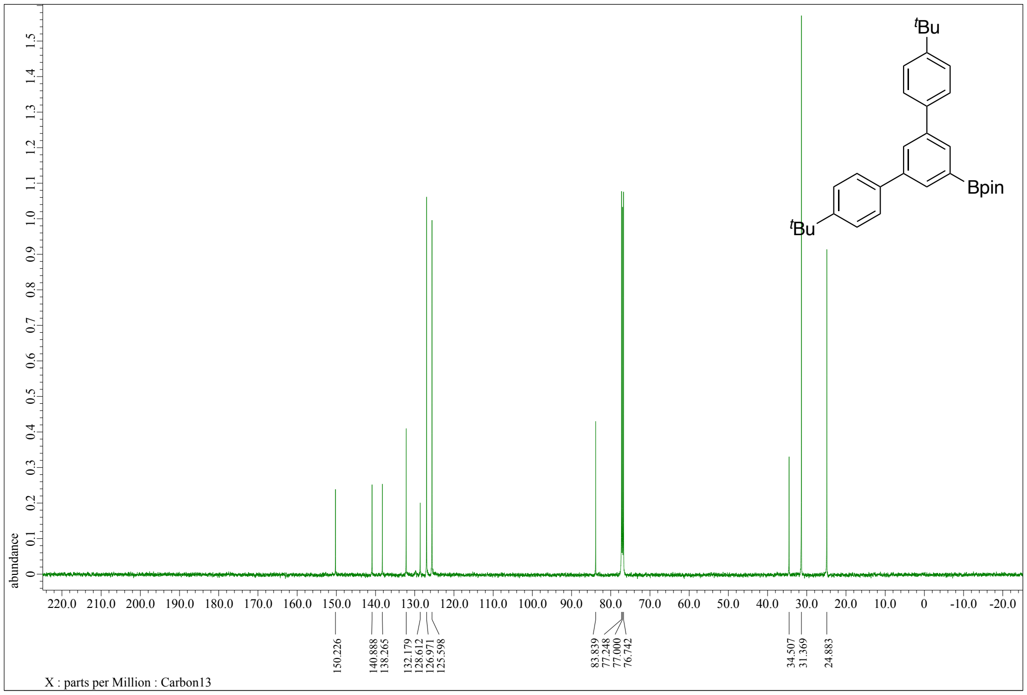

Supplementary Figure 47. ^{13}C NMR spectrum of **2f** (125 MHz, $CDCl_3$)

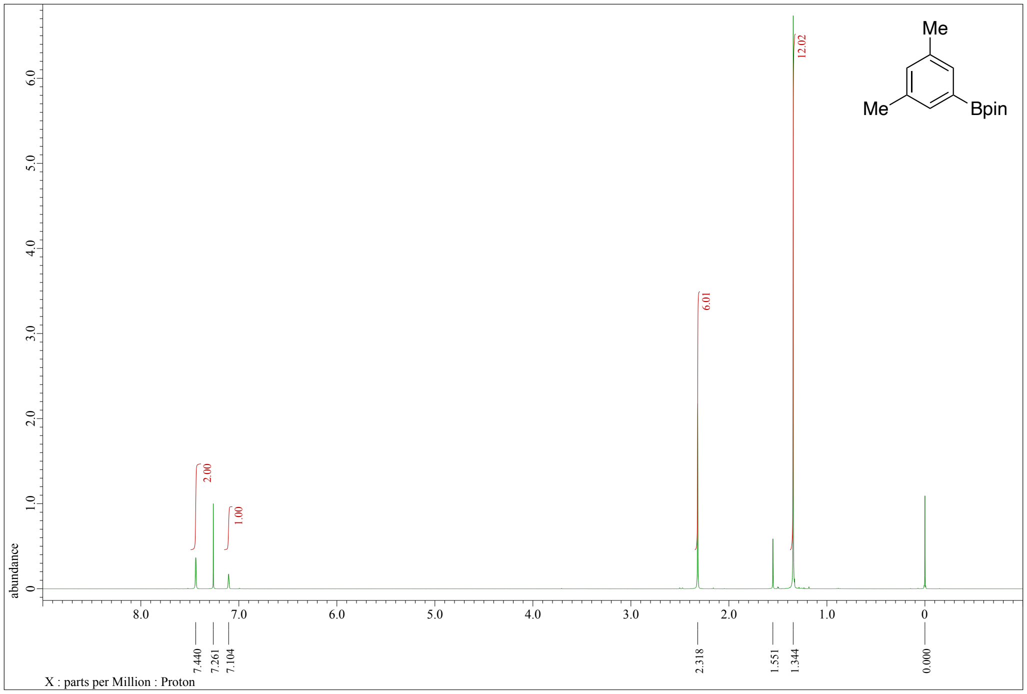

Supplementary Figure 48. ^{1}H NMR spectrum of **2g** (500 MHz, $CDCl_3$)

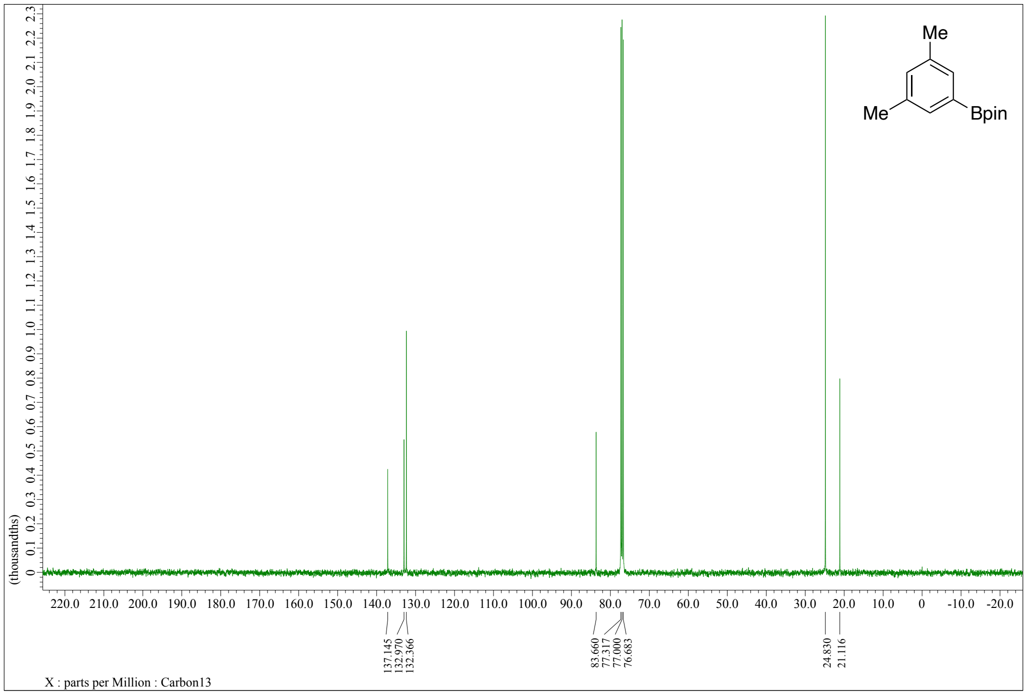

Supplementary Figure 49. ^{13}C NMR spectrum of **2g** (125 MHz, $CDCl_3$)

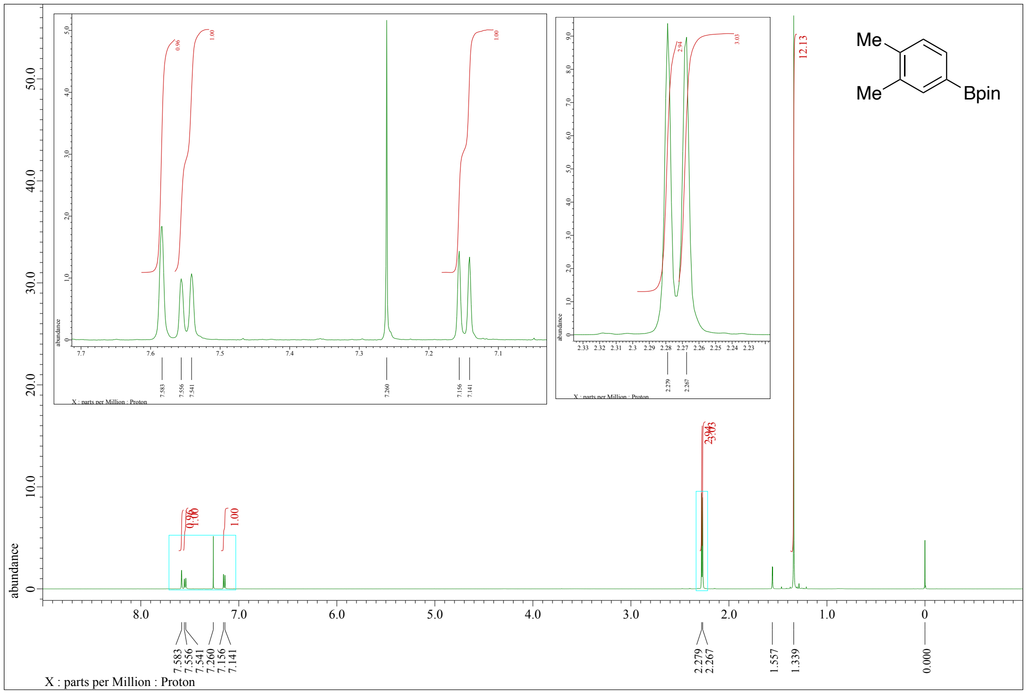

Supplementary Figure 50. ^{1}H NMR spectrum of **2h** (500 MHz, $CDCl_3$)

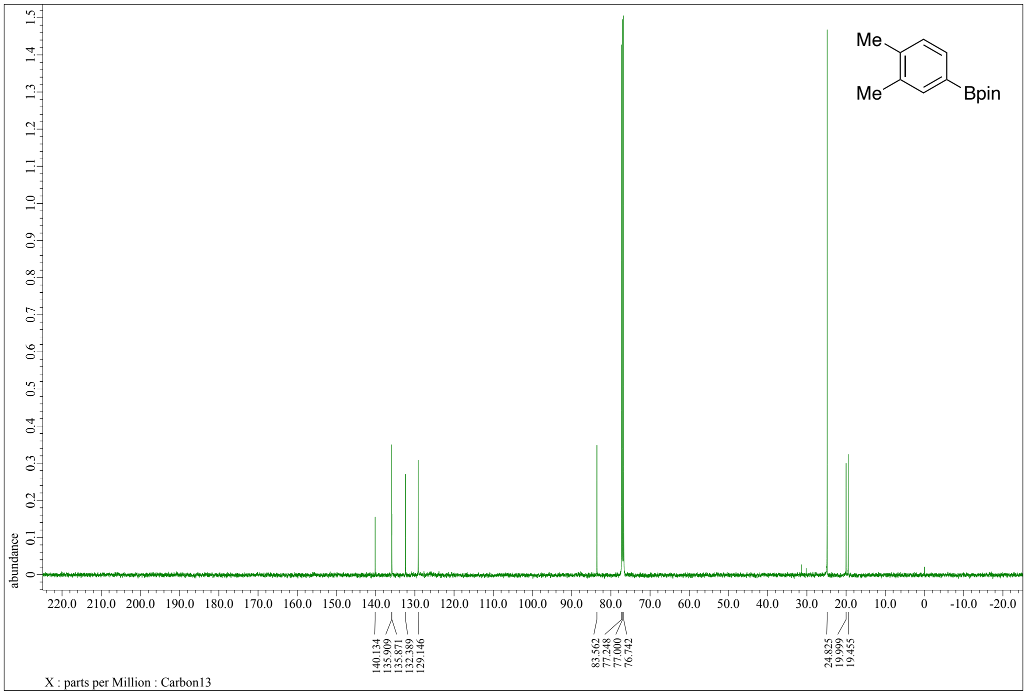

Supplementary Figure 51. ^{13}C NMR spectrum of **2h** (125 MHz, $CDCl_3$)

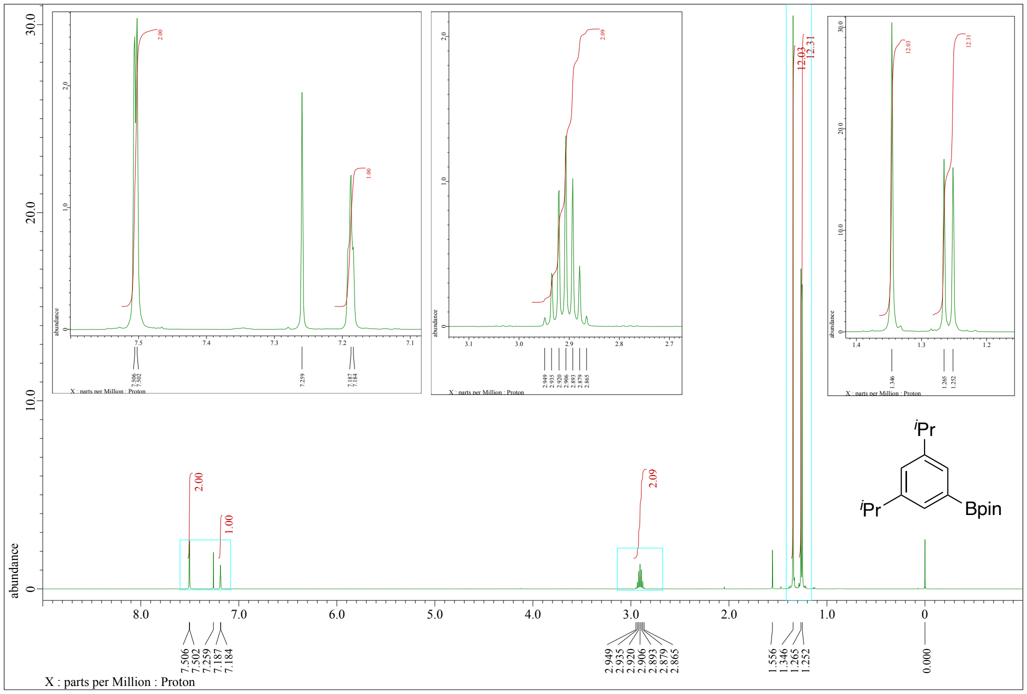

Supplementary Figure 52. ^{1}H NMR spectrum of **2i** (500 MHz, $CDCl_3$)

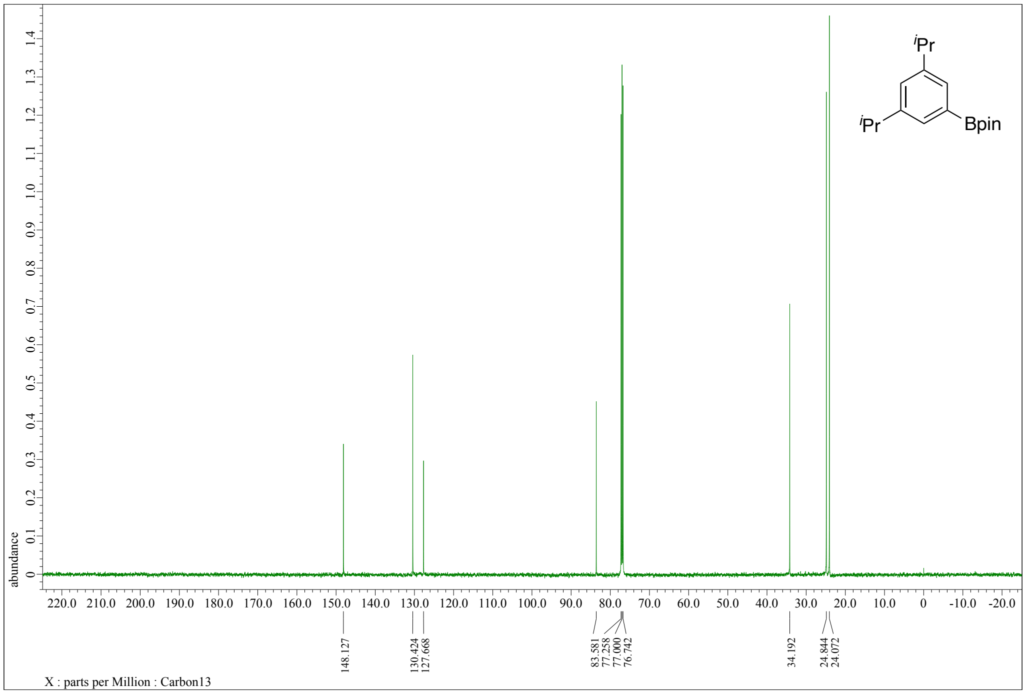

Supplementary Figure 53. ^{13}C NMR spectrum of **2i** (125 MHz, $CDCl_3$)

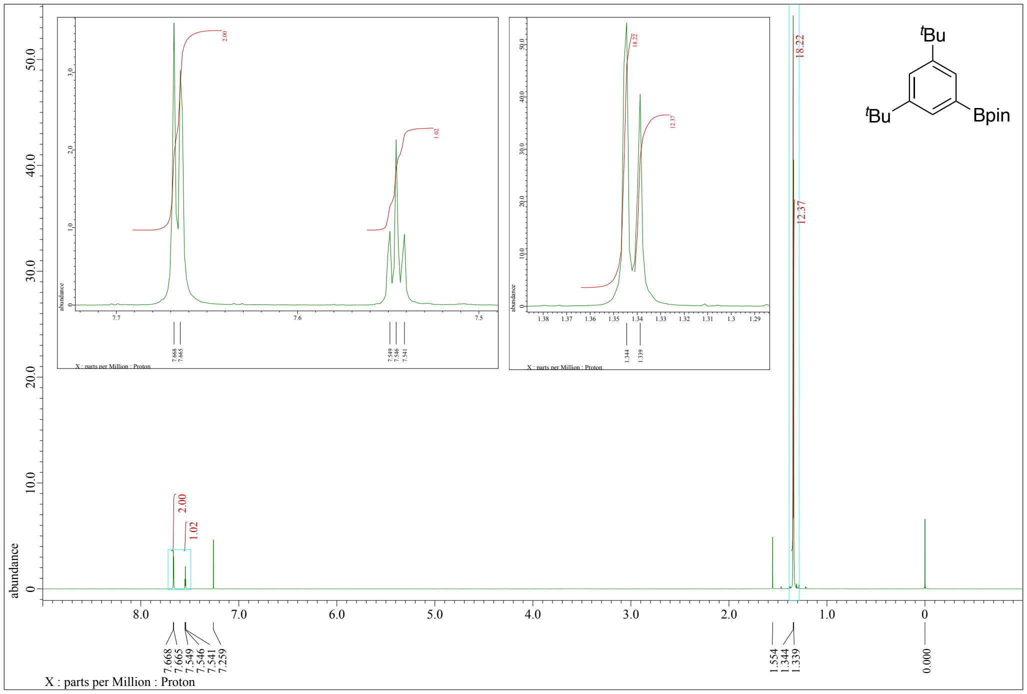

Supplementary Figure 54. ^{1}H NMR spectrum of **2j** (500 MHz, $CDCl_3$)

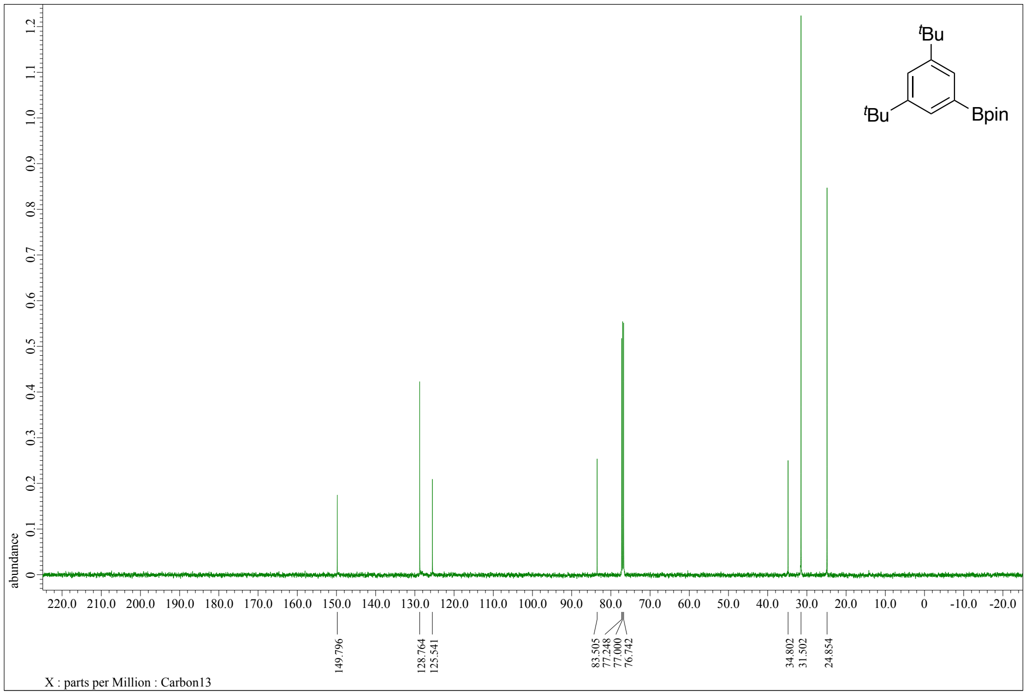

Supplementary Figure 55. ^{13}C NMR spectrum of **2j** (125 MHz, $CDCl_3$)

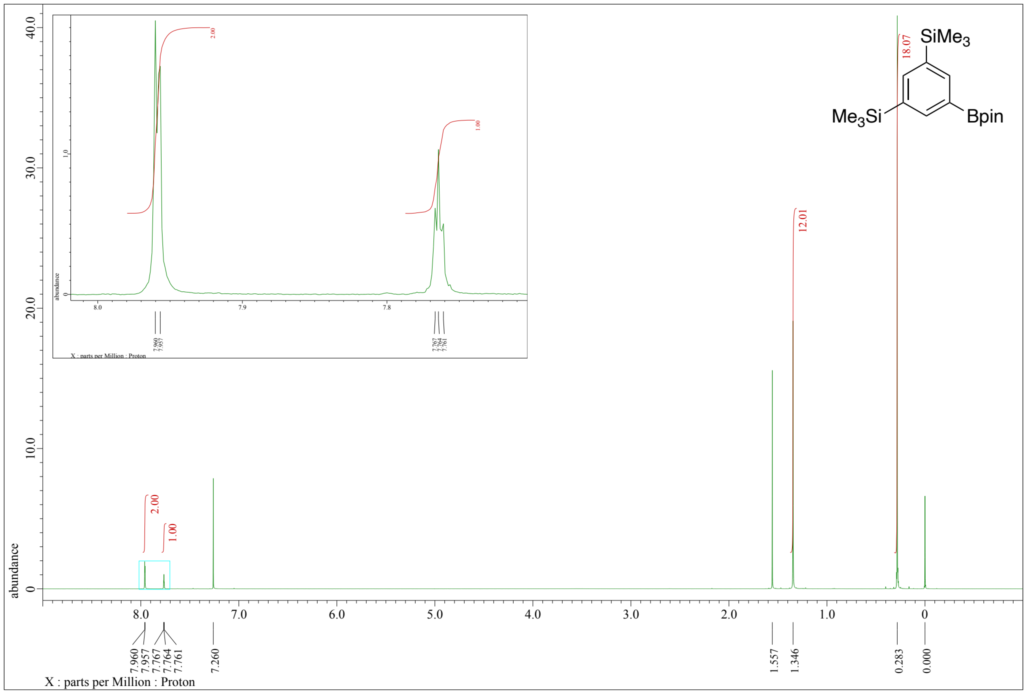

Supplementary Figure 56. ^{1}H NMR spectrum of **2k** (500 MHz, $CDCl_3$)

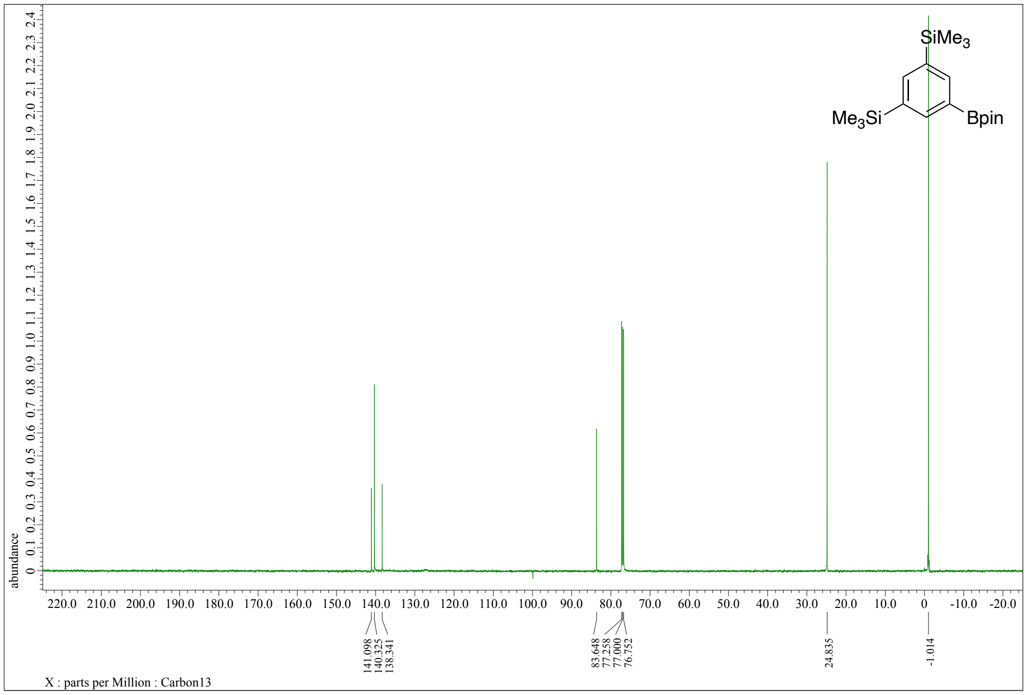

Supplementary Figure 57. ^{13}C NMR spectrum of **2k** (125 MHz, $CDCl_3$)

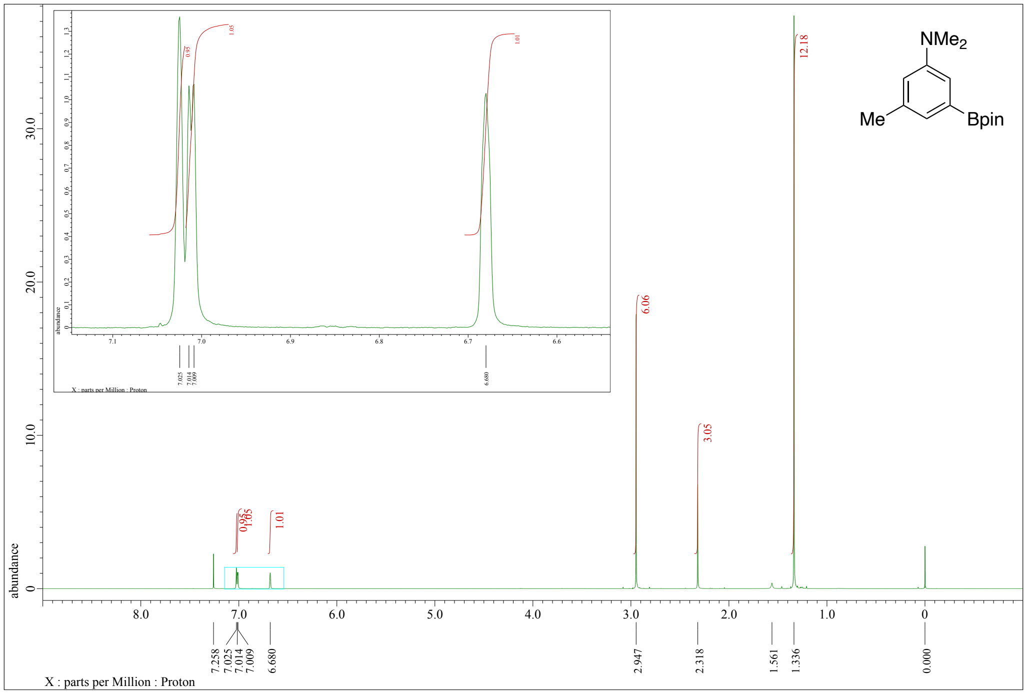

Supplementary Figure 58. ^{1}H NMR spectrum of **2l** (500 MHz, $CDCl_3$)

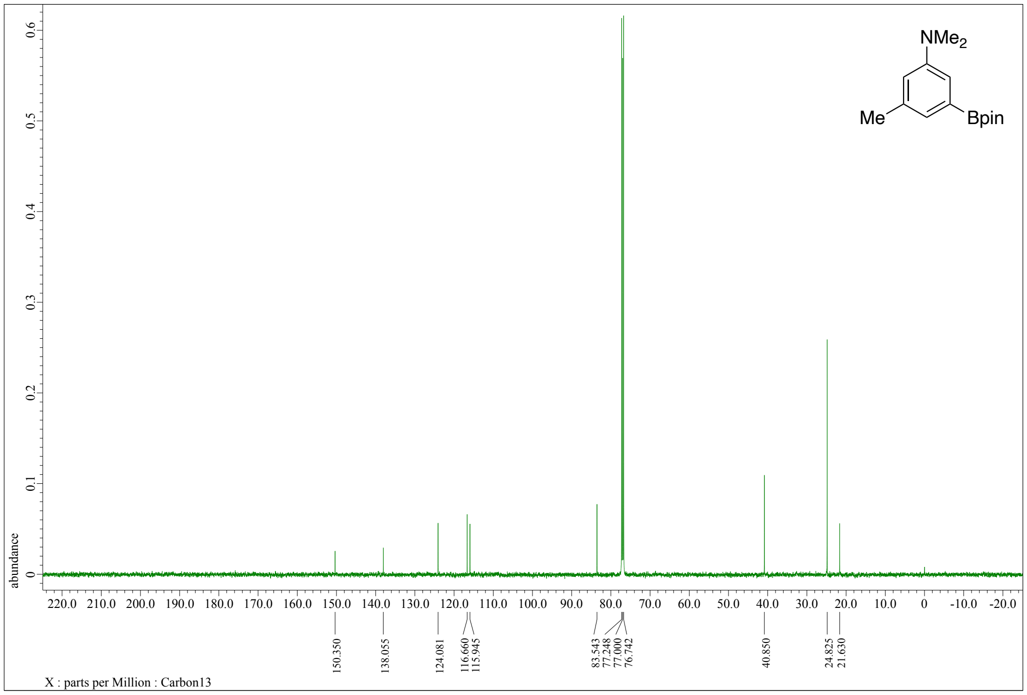

Supplementary Figure 59. ^{13}C NMR spectrum of **2l** (125 MHz, $CDCl_3$)

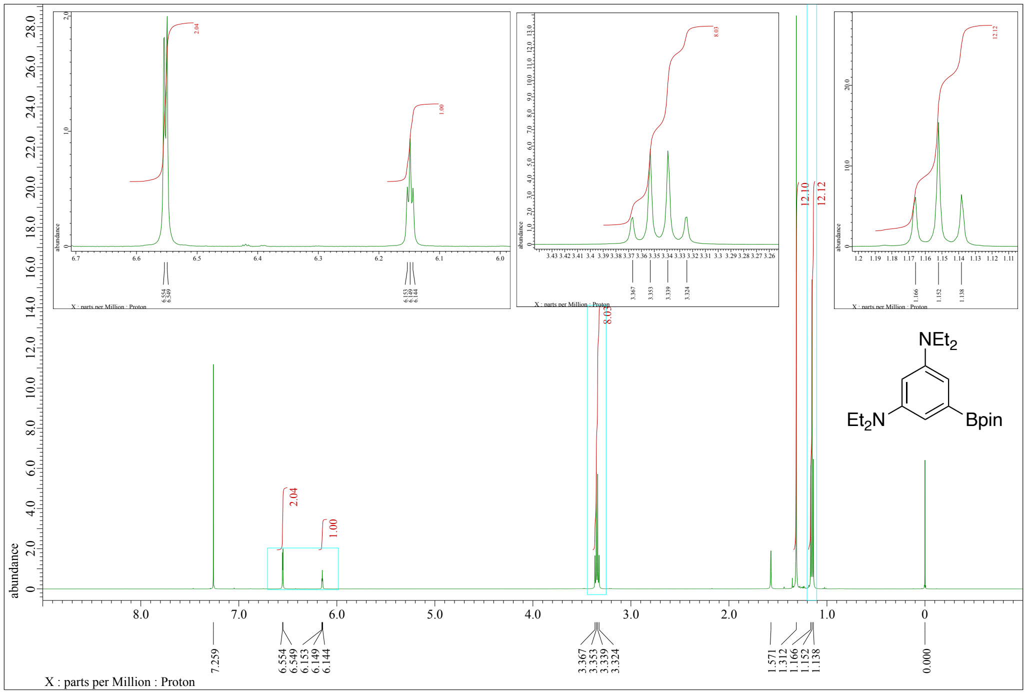

Supplementary Figure 60. ^{1}H NMR spectrum of **2m** (500 MHz, $CDCl_3$)

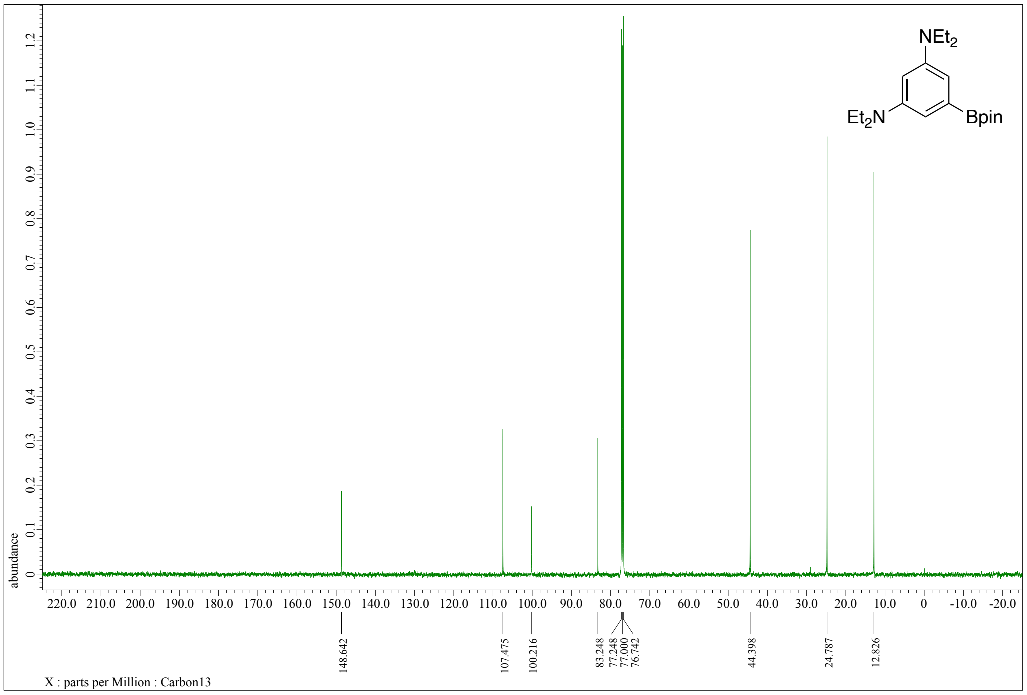

Supplementary Figure 61. ^{13}C NMR spectrum of **2m** (125 MHz, $CDCl_3$)

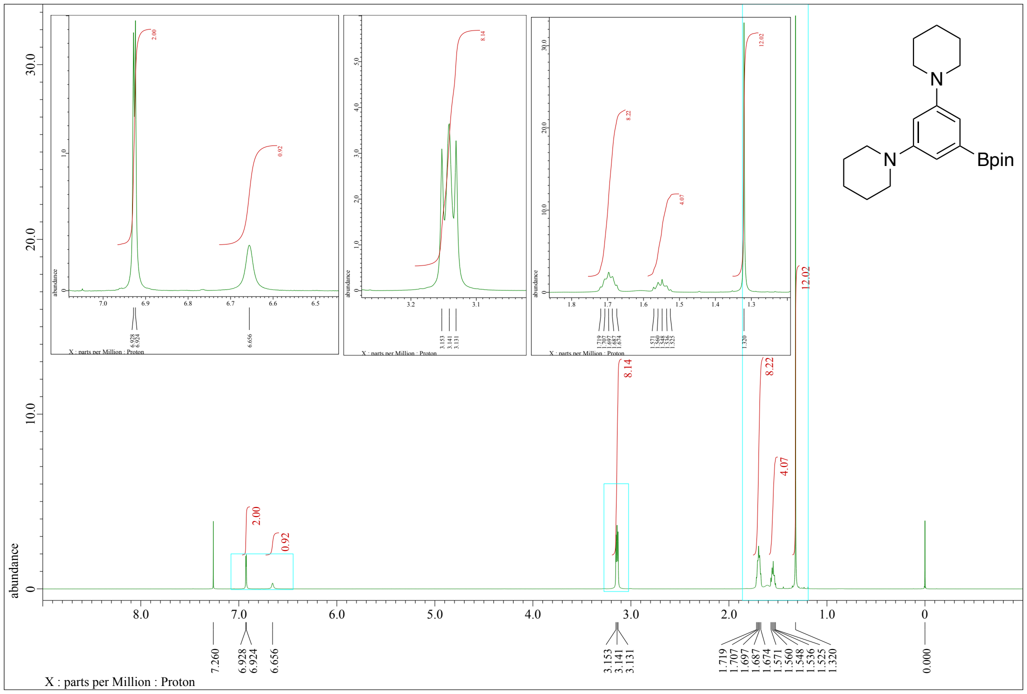

Supplementary Figure 62. ^{1}H NMR spectrum of **2n** (500 MHz, $CDCl_3$)

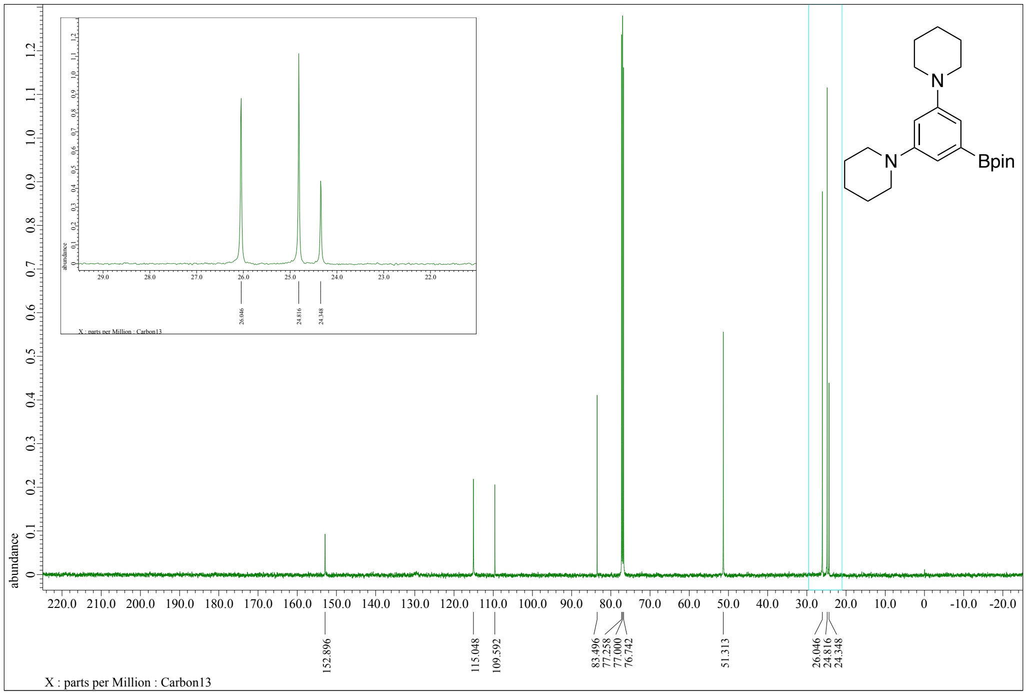

Supplementary Figure 63. ^{13}C NMR spectrum of **2n** (125 MHz, $CDCl_3$)

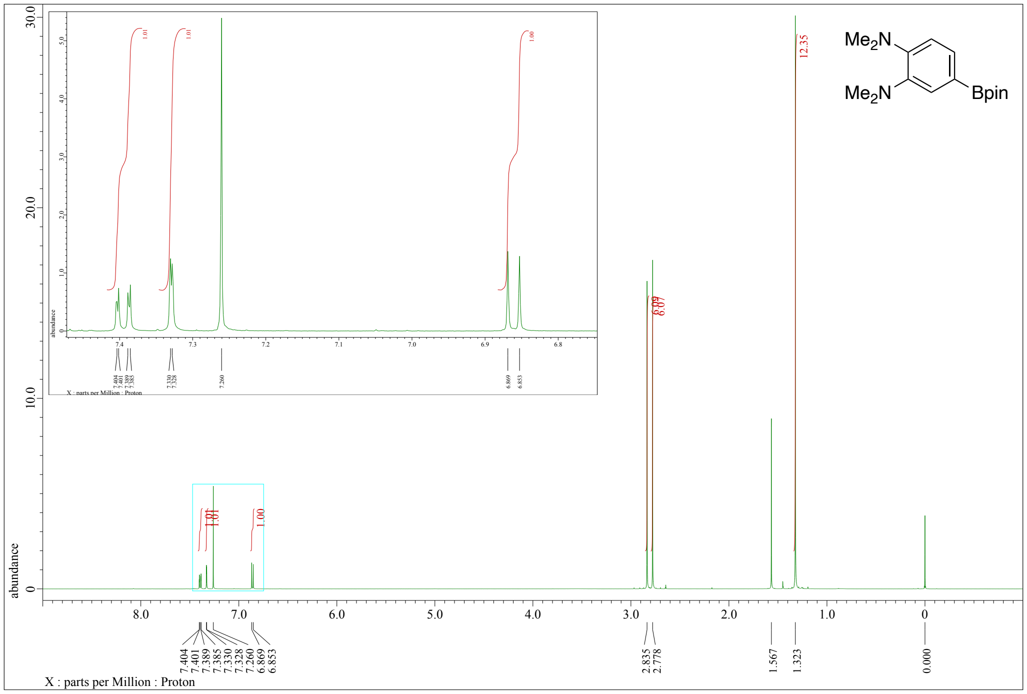

Supplementary Figure 64. ^{1}H NMR spectrum of **2o** (500 MHz, $CDCl_3$)

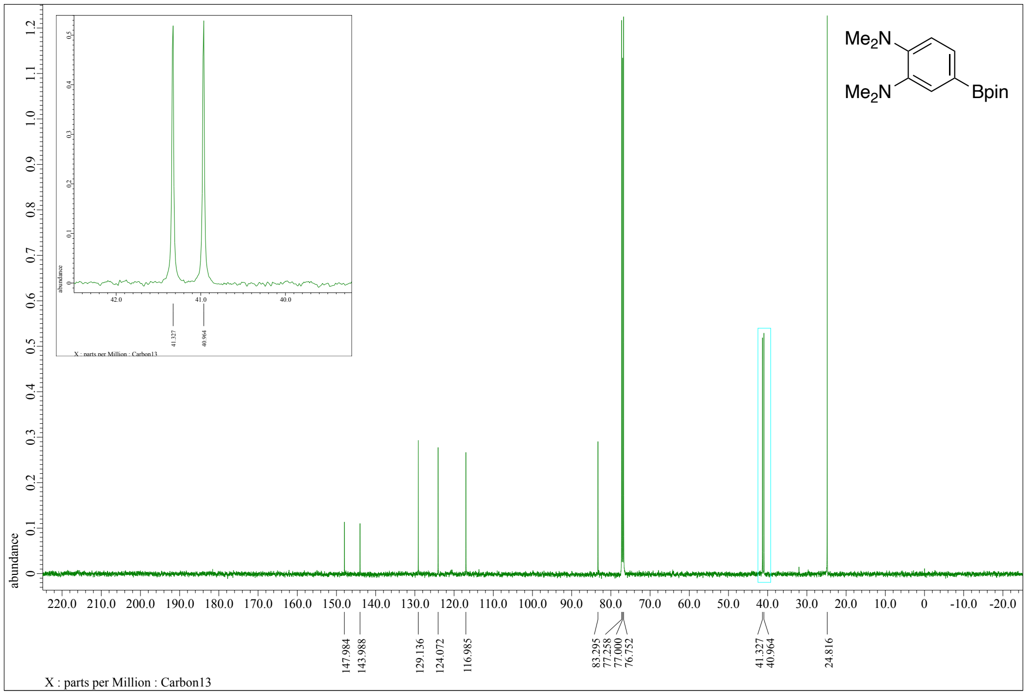

Supplementary Figure 65. ^{13}C NMR spectrum of **2o** (125 MHz, $CDCl_3$)

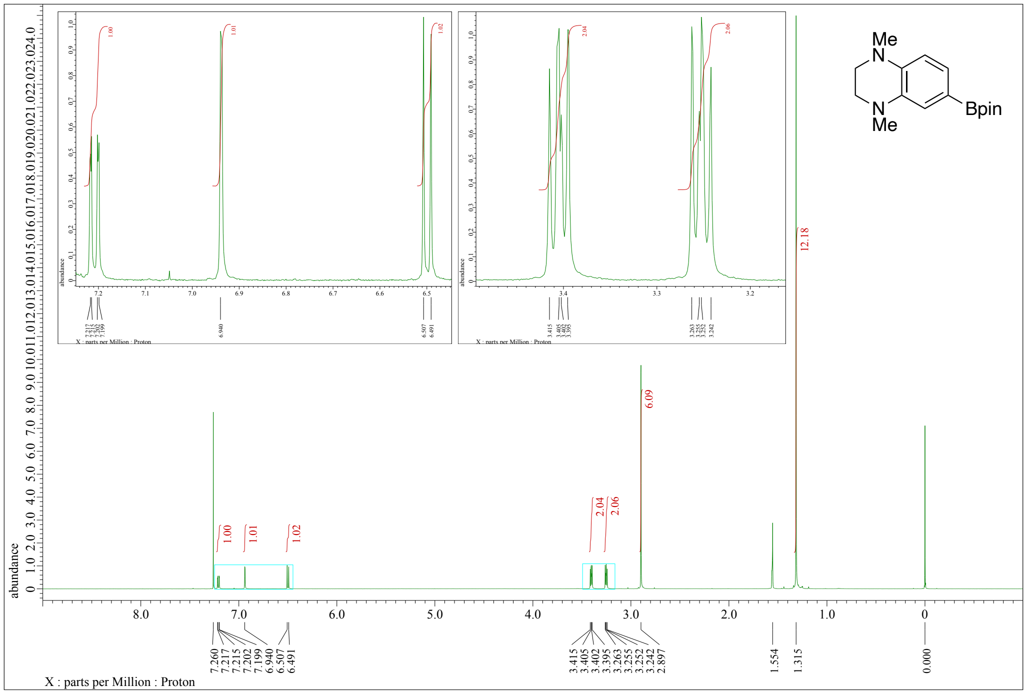

Supplementary Figure 66. ^{1}H NMR spectrum of **2p** (500 MHz, $CDCl_3$)

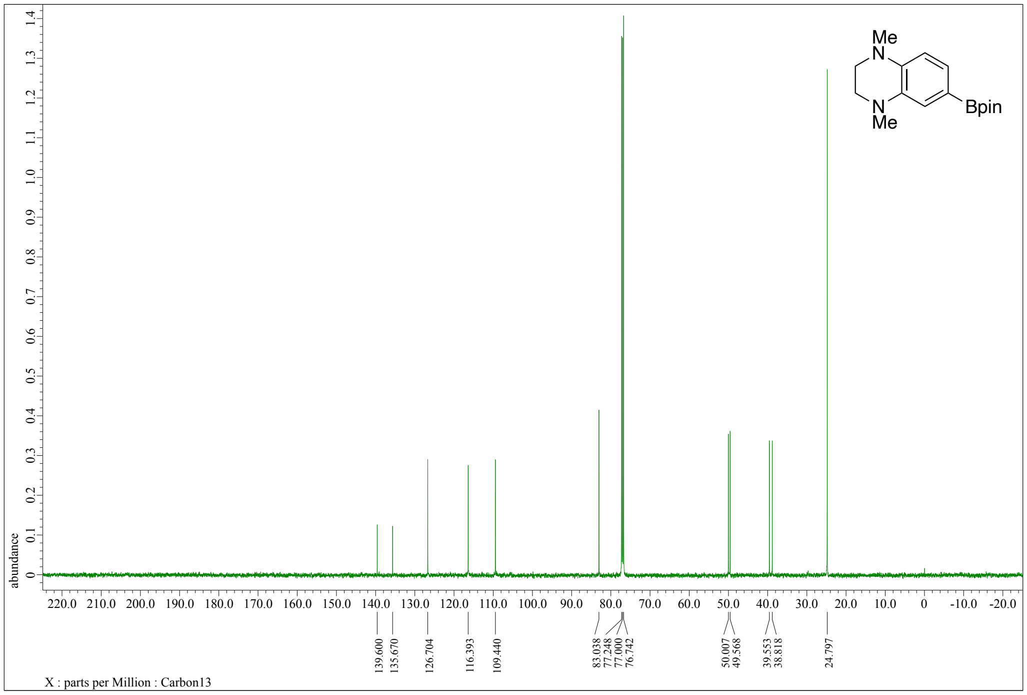

Supplementary Figure 67. ^{13}C NMR spectrum of **2p** (125 MHz, $CDCl_3$)

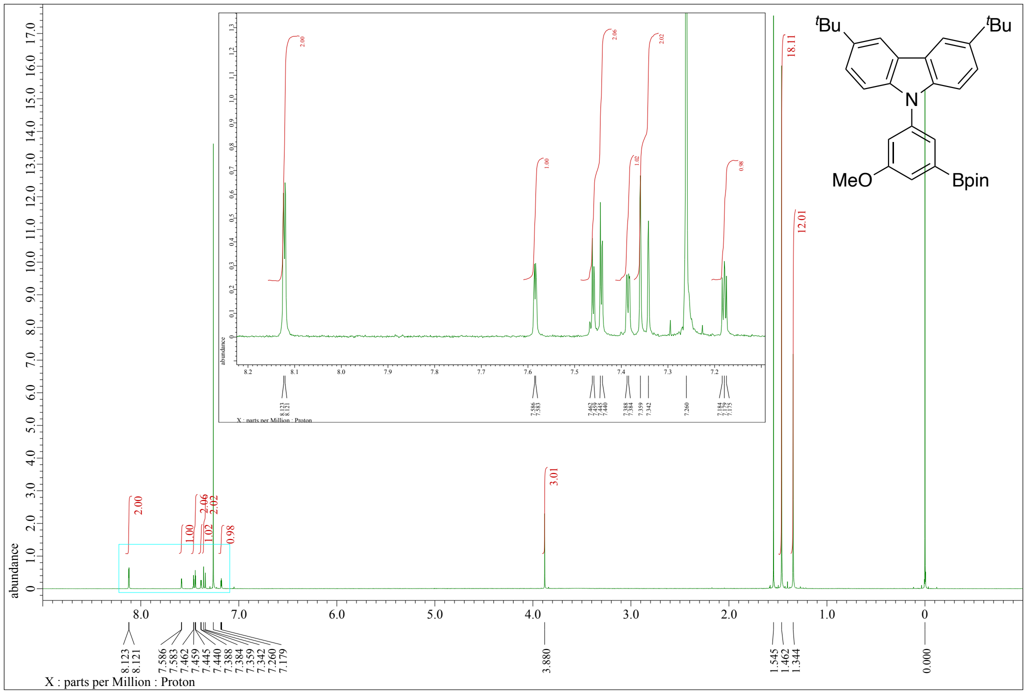

Supplementary Figure 68. ^{1}H NMR spectrum of **2q** (500 MHz, $CDCl_3$)

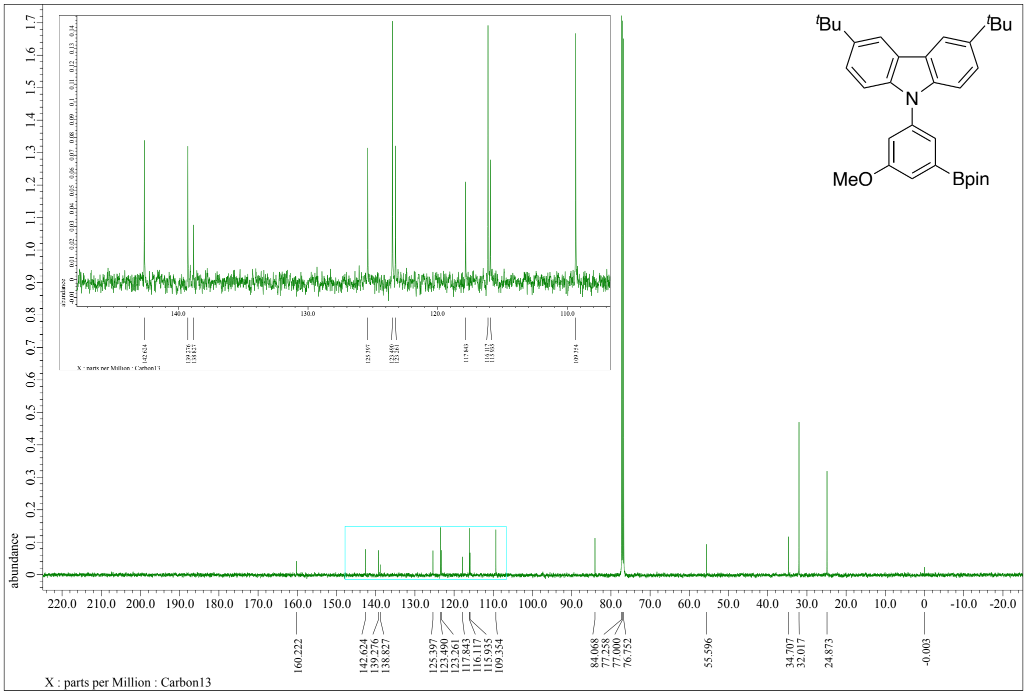

Supplementary Figure 69. ^{13}C NMR spectrum of **2q** (125 MHz, $CDCl_3$)

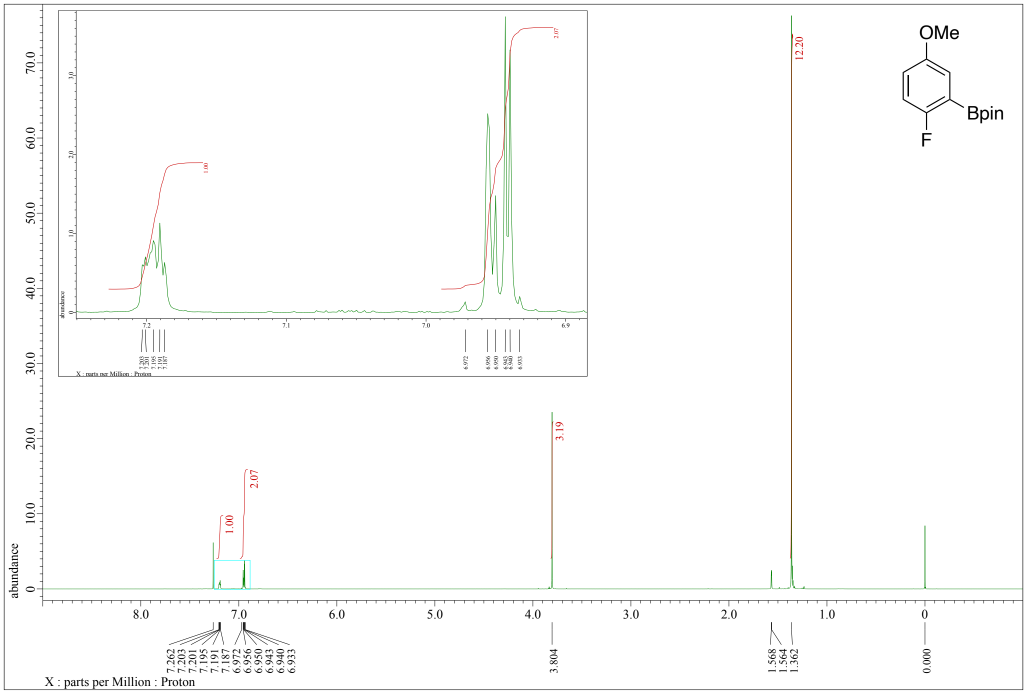

Supplementary Figure 70. ^{1}H NMR spectrum of **2r** (500 MHz, $CDCl_3$)

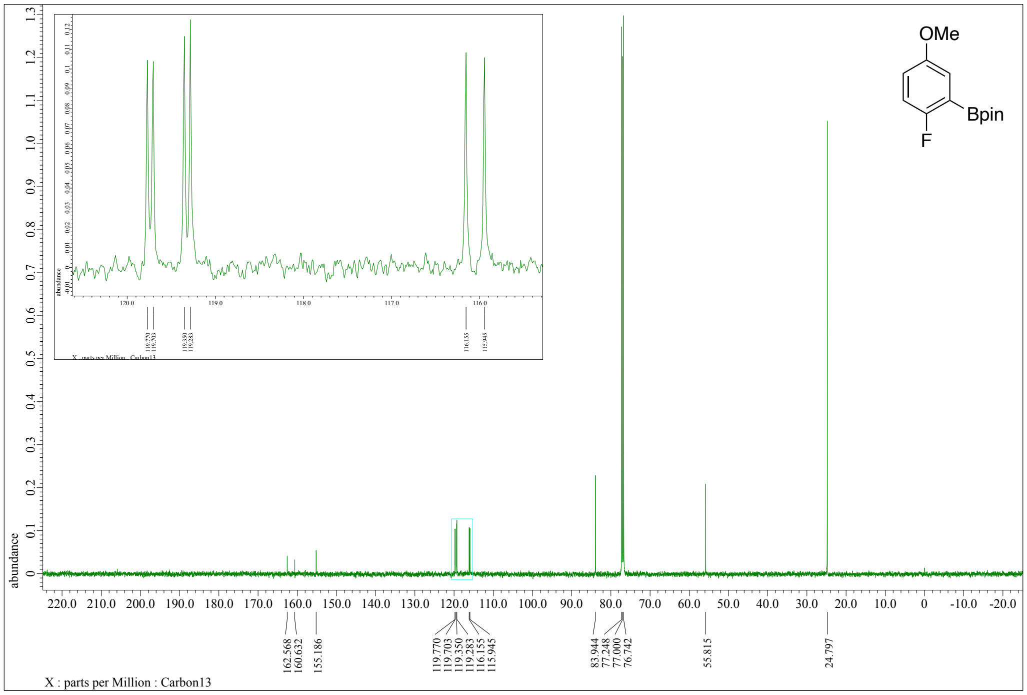

Supplementary Figure 71. ^{13}C NMR spectrum of **2r** (125 MHz, $CDCl_3$)

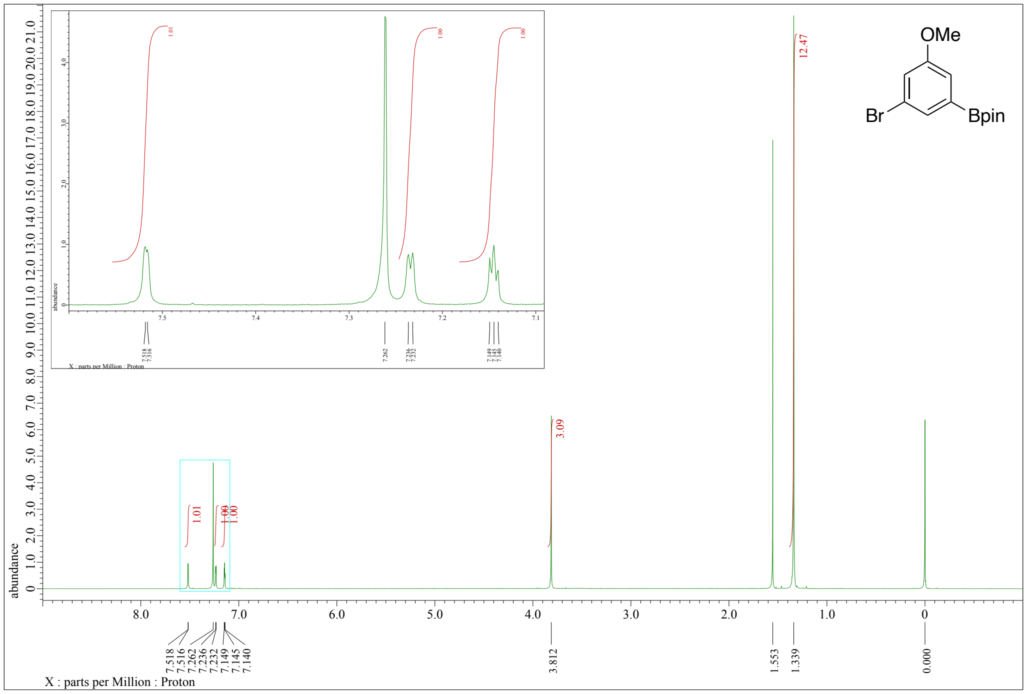

Supplementary Figure 72. ^{1}H NMR spectrum of **2s** (500 MHz, $CDCl_3$)

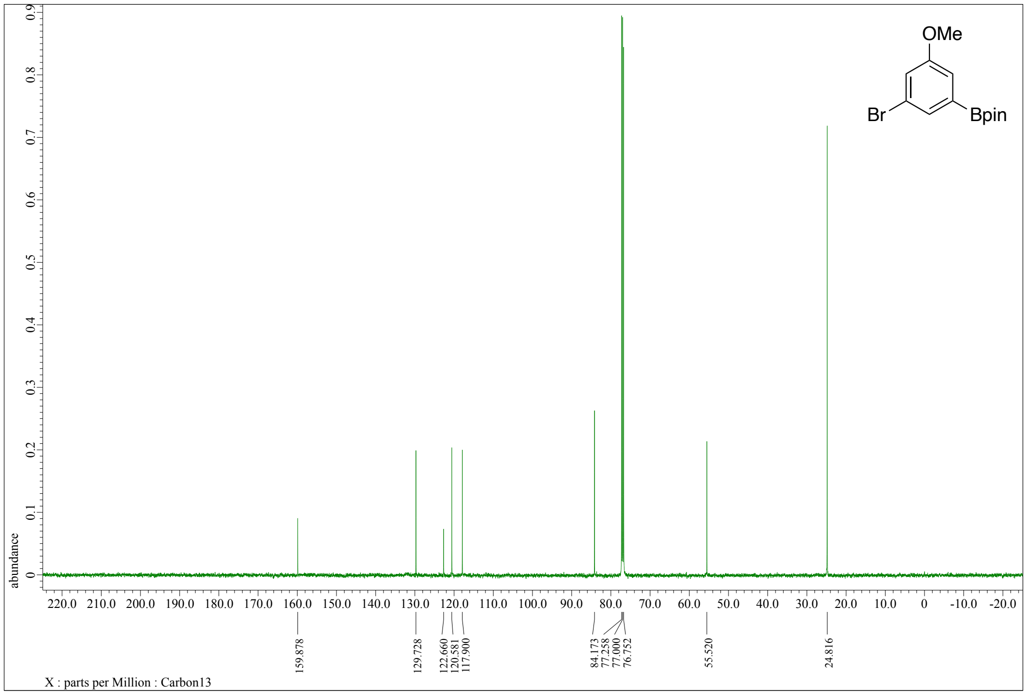

Supplementary Figure 73. ^{13}C NMR spectrum of **2s** (125 MHz, $CDCl_3$)

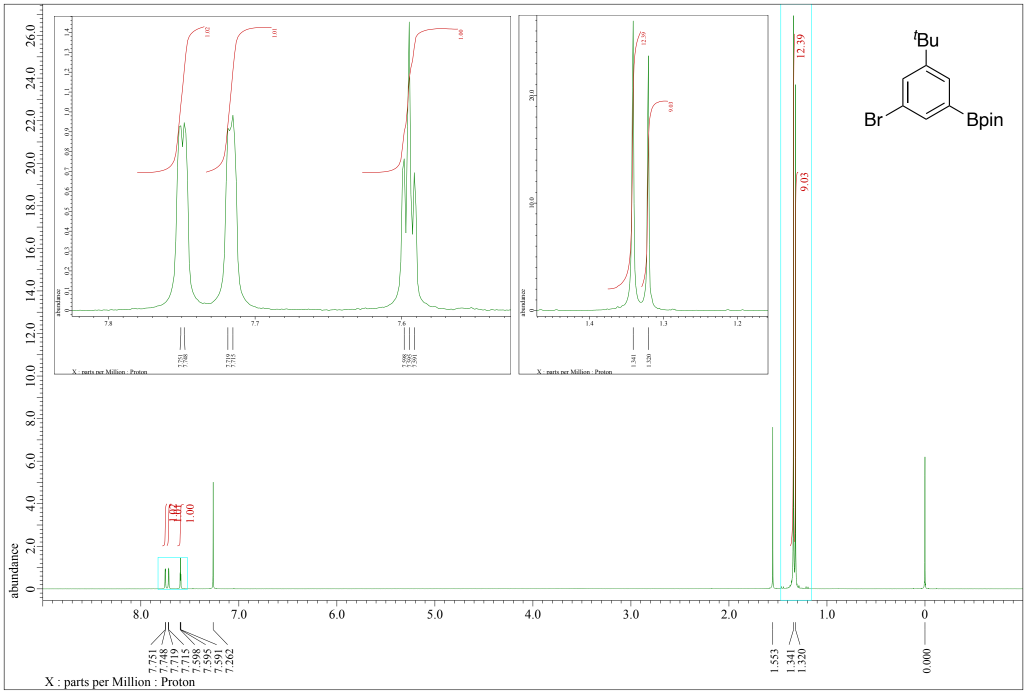

Supplementary Figure 74. ^{1}H NMR spectrum of **2t** (500 MHz, $CDCl_3$)

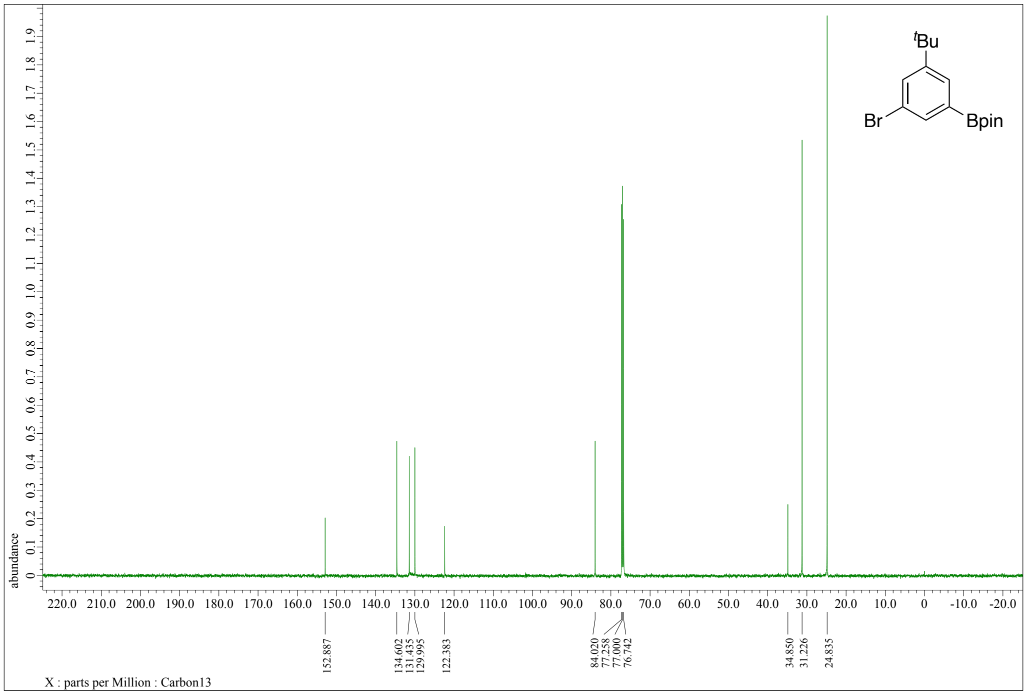

Supplementary Figure 75. ^{13}C NMR spectrum of **2t** (125 MHz, $CDCl_3$)

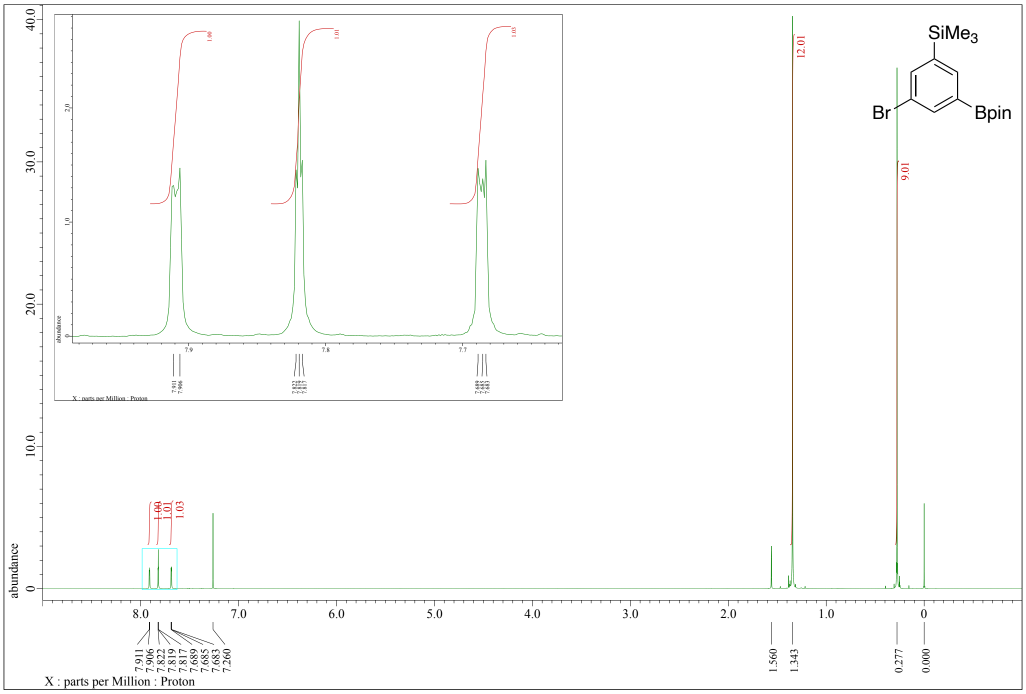

Supplementary Figure 76. ^{1}H NMR spectrum of **2u** (500 MHz, $CDCl_3$)

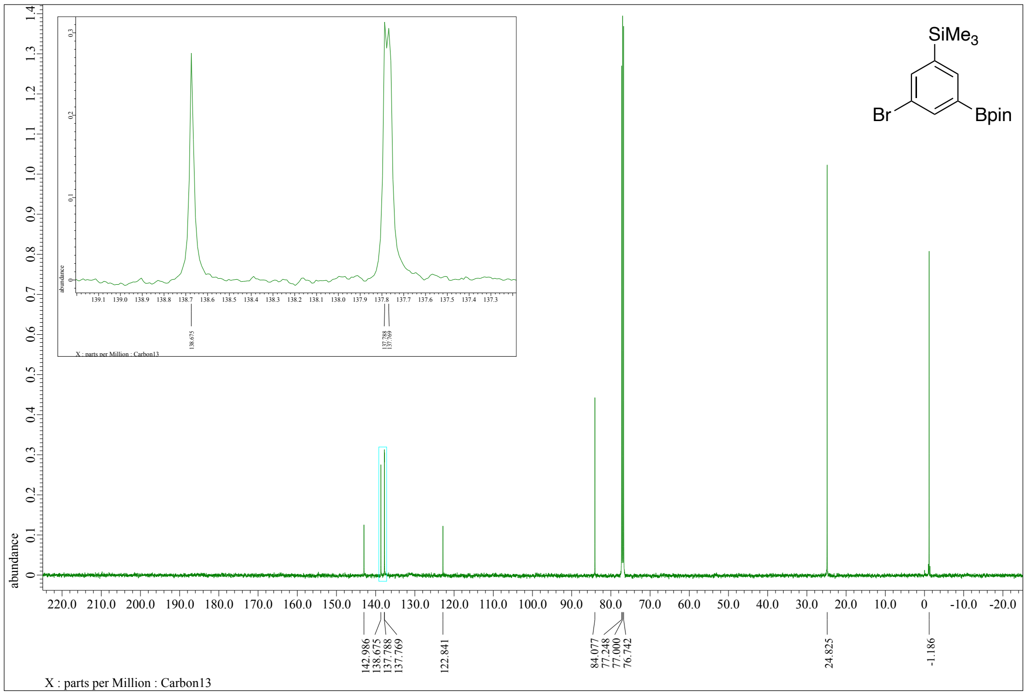

Supplementary Figure 77. ^{13}C NMR spectrum of **2u** (125 MHz, $CDCl_3$)

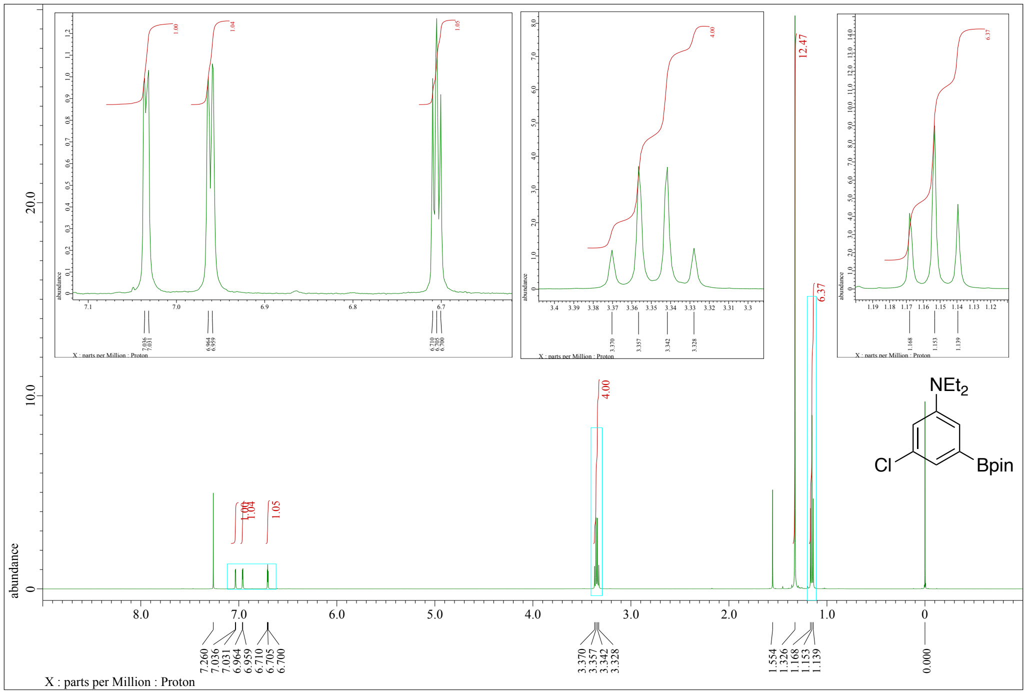

Supplementary Figure 78. ^{1}H NMR spectrum of **2v** (500 MHz, $CDCl_3$)

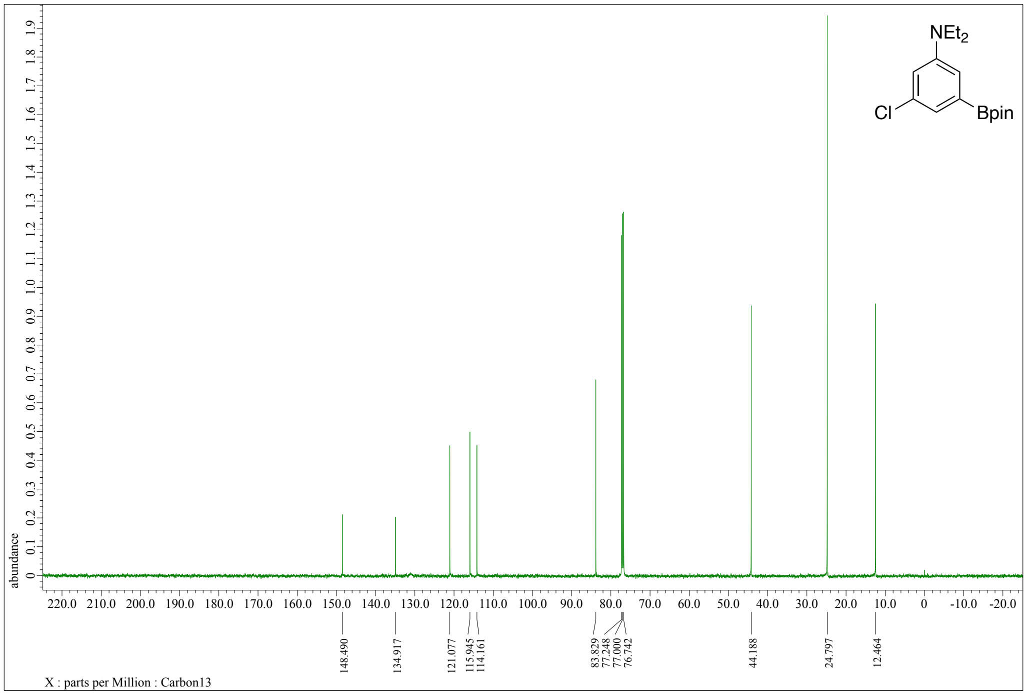

Supplementary Figure 79. ^{13}C NMR spectrum of **2v** (125 MHz, $CDCl_3$)

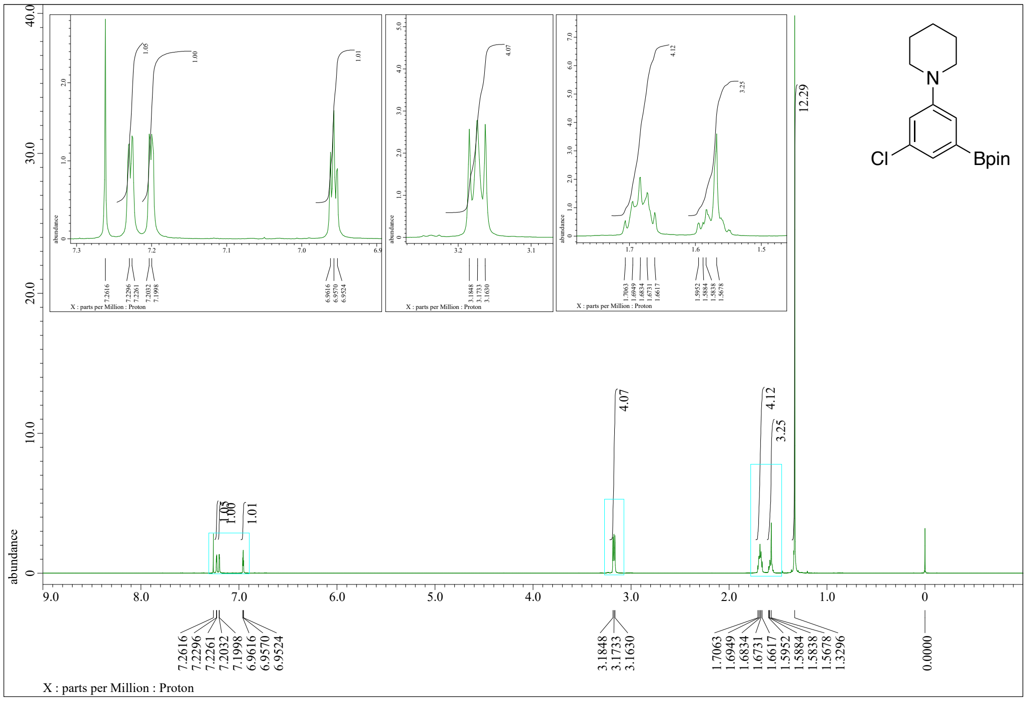

Supplementary Figure 80. ^{1}H NMR spectrum of **2w** (500 MHz, $CDCl_3$)

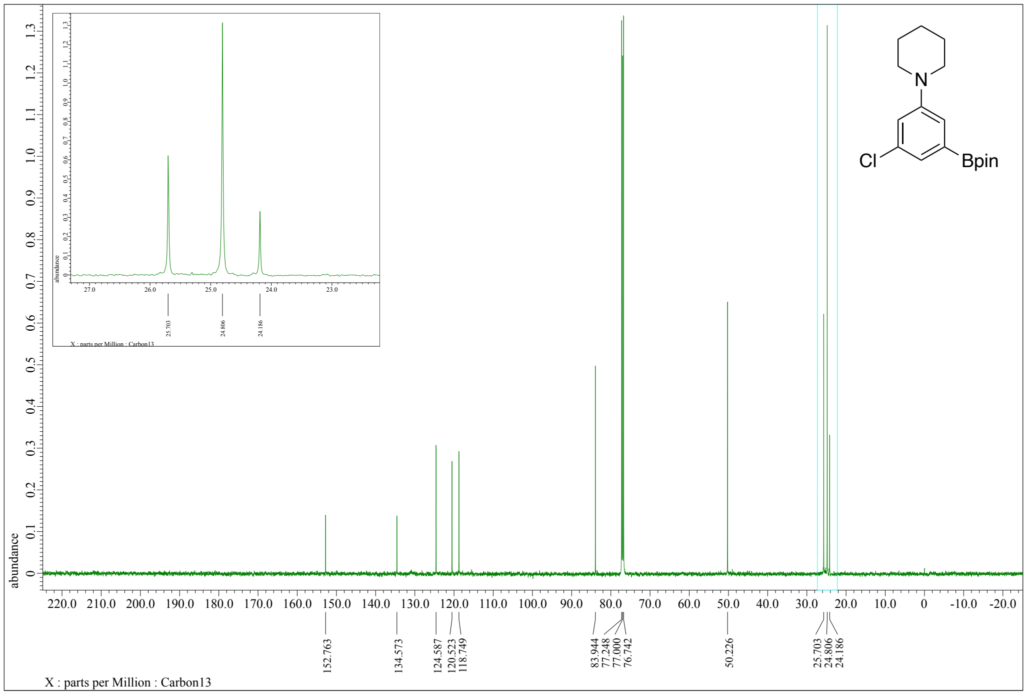

Supplementary Figure 81. ^{13}C NMR spectrum of **2w** (125 MHz, $CDCl_3$)

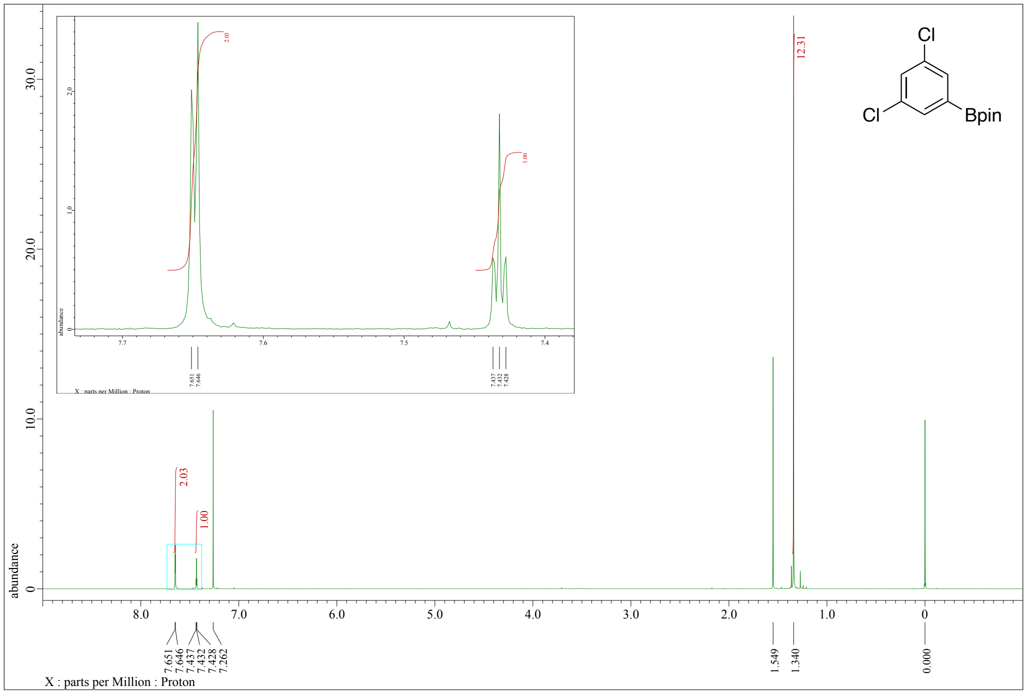

Supplementary Figure 82. ^{1}H NMR spectrum of **2x** (500 MHz, $CDCl_3$)

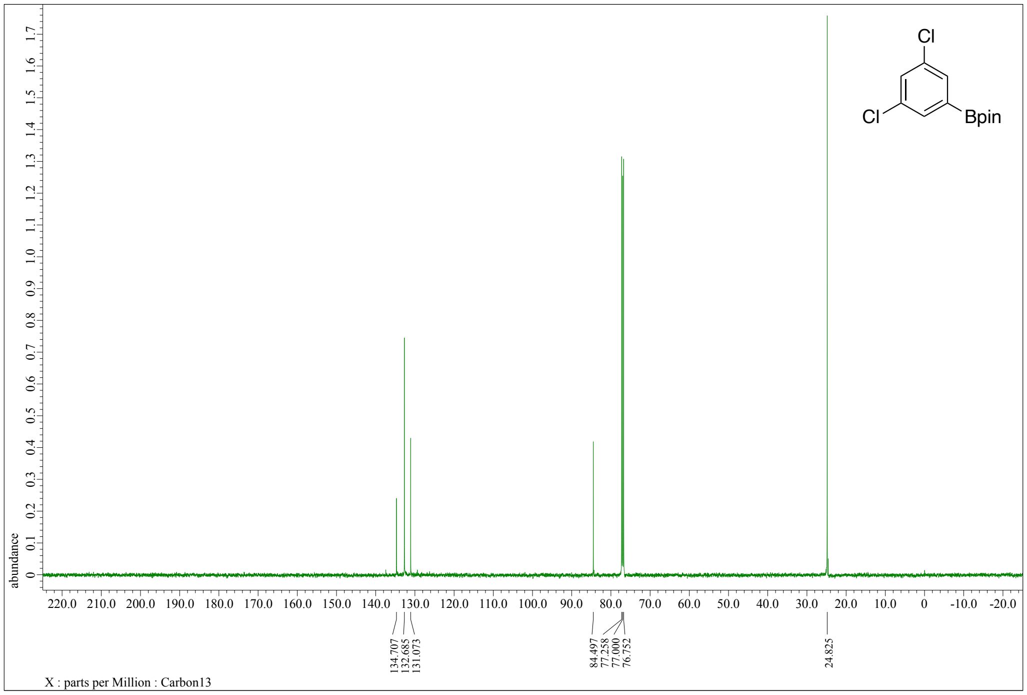

Supplementary Figure 83. ^{13}C NMR spectrum of **2x** (125 MHz, $CDCl_3$)

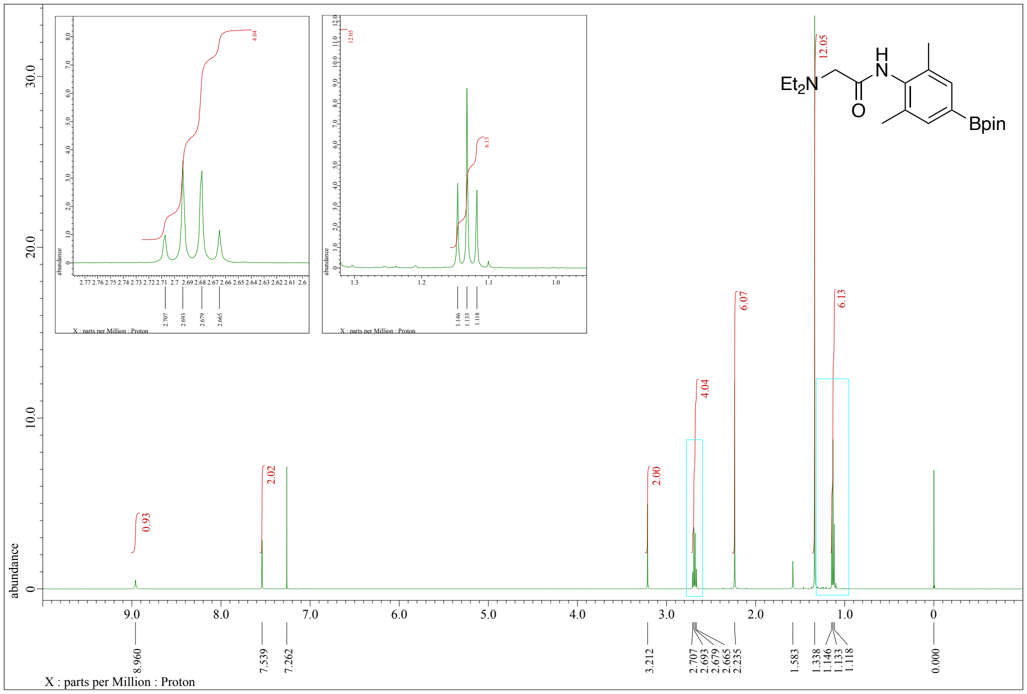

Supplementary Figure 84. ^{1}H NMR spectrum of **4a** (500 MHz, $CDCl_3$)

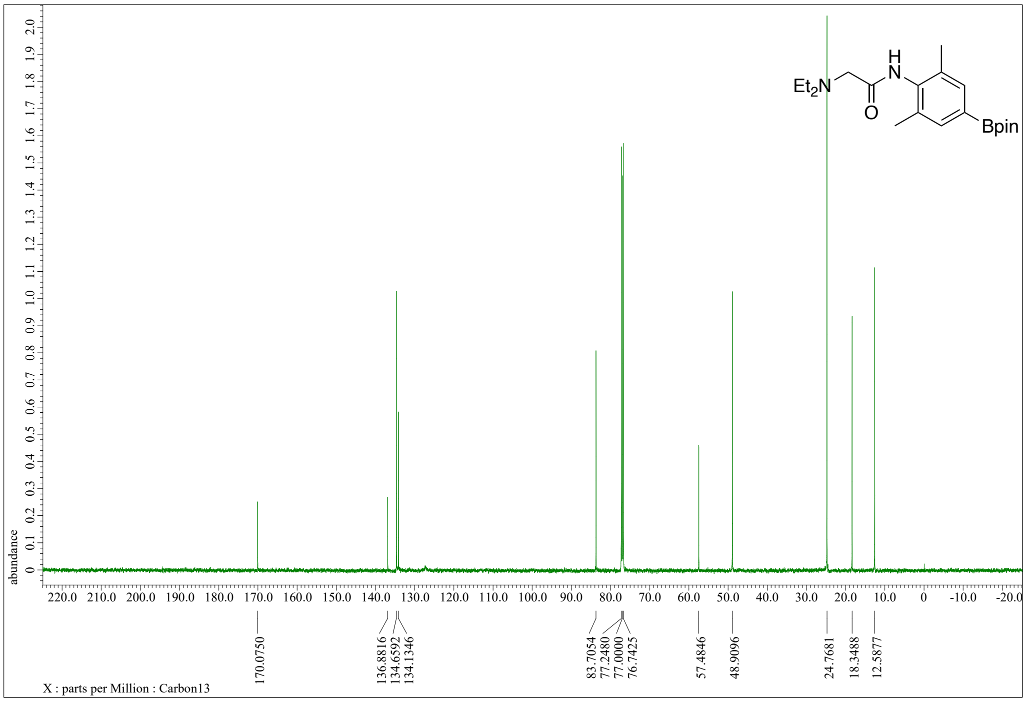

Supplementary Figure 85. ^{13}C NMR spectrum of **4a** (125 MHz, $CDCl_3$)

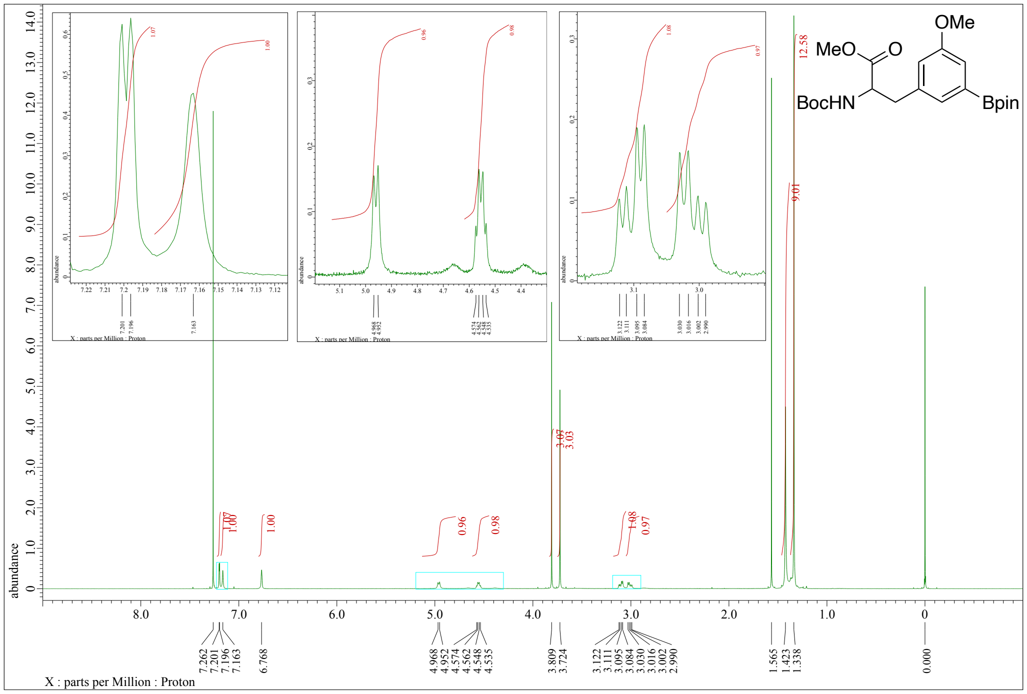

Supplementary Figure 86. ^{1}H NMR spectrum of **4b** (500 MHz, $CDCl_3$)

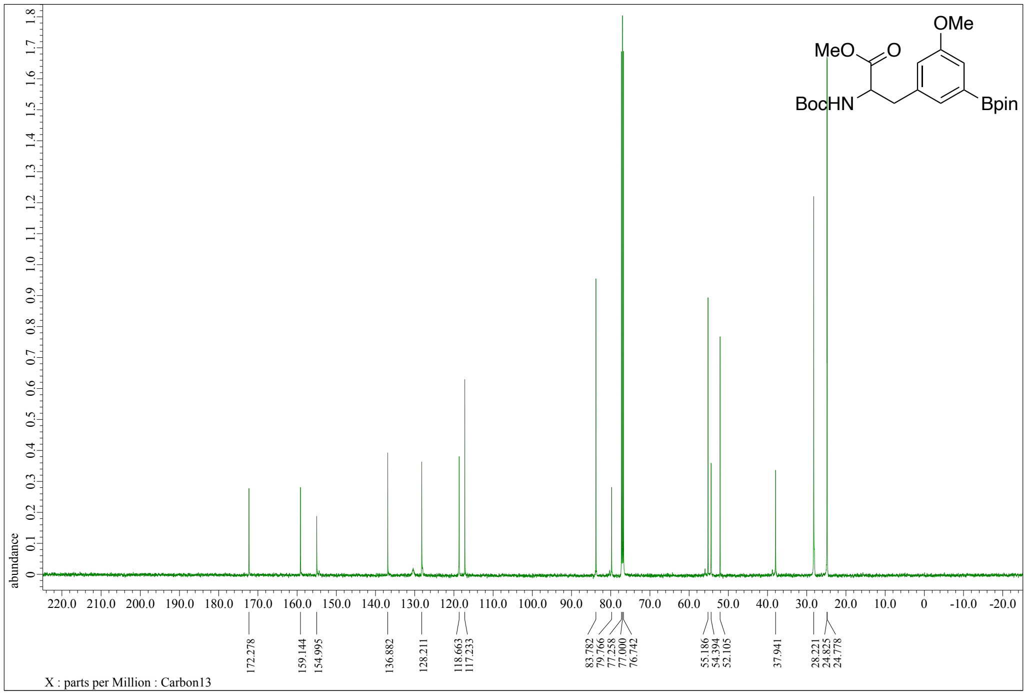

Supplementary Figure 87. ^{13}C NMR spectrum of **4b** (125 MHz, $CDCl_3$)

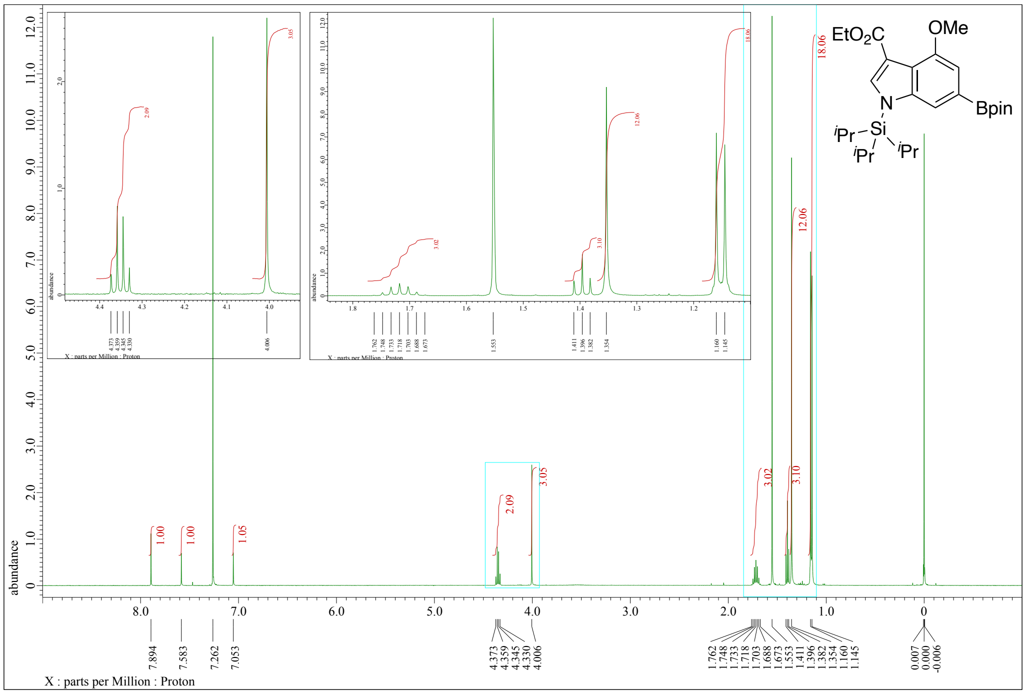

Supplementary Figure 88. ^{1}H NMR spectrum of **4c** (500 MHz, $CDCl_3$)

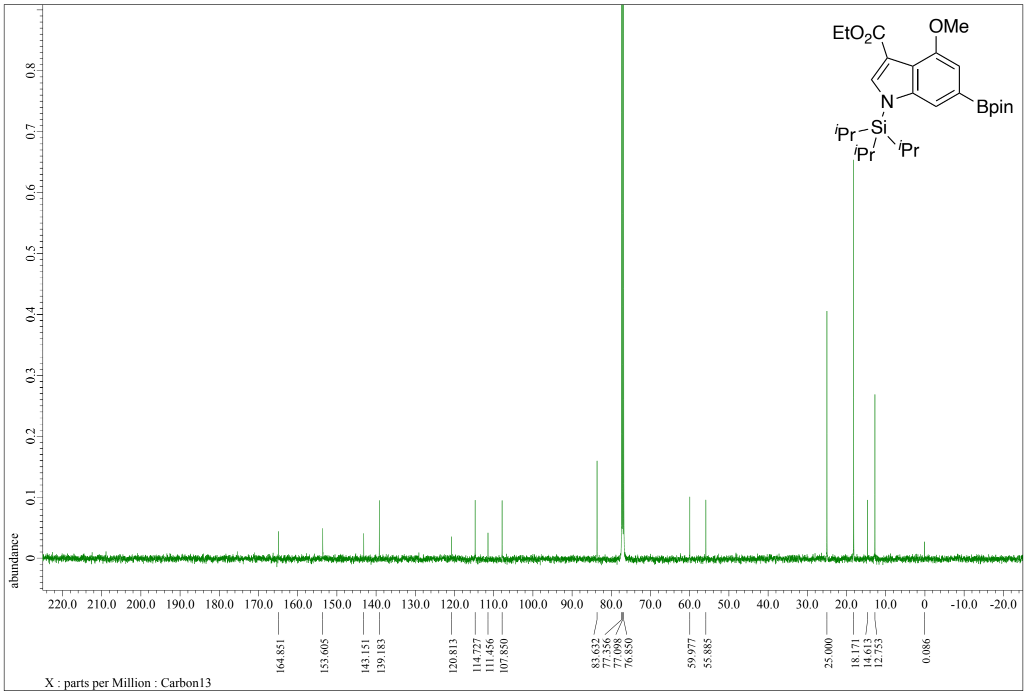

Supplementary Figure 89. ^{13}C NMR spectrum of **4c** (125 MHz, $CDCl_3$)

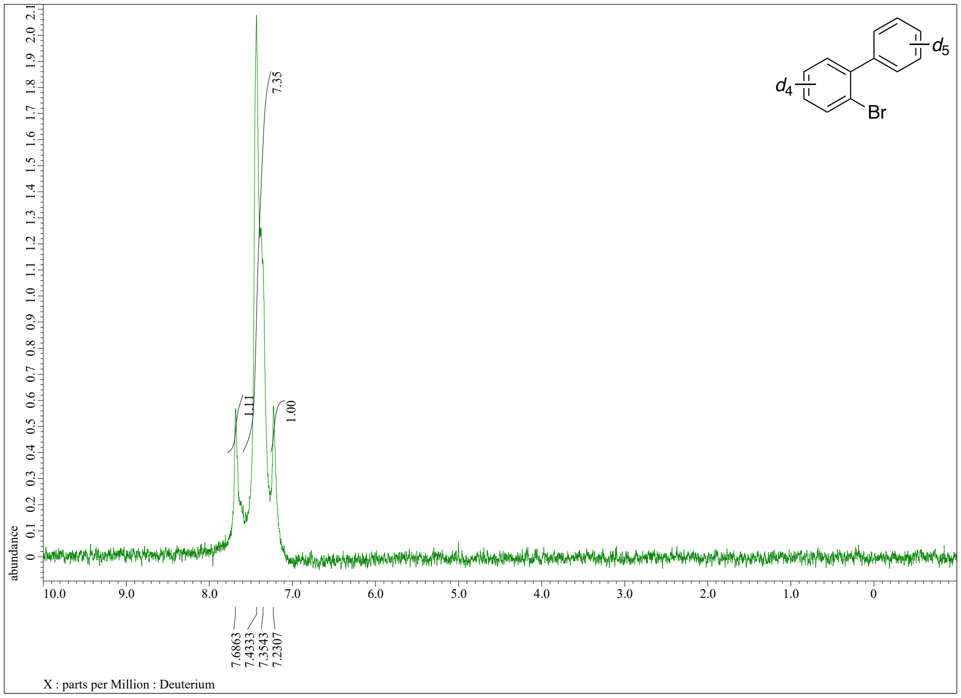

Supplementary Figure 90. ^{2}H NMR spectrum of **S1** (77 MHz, $CHCl_3$)

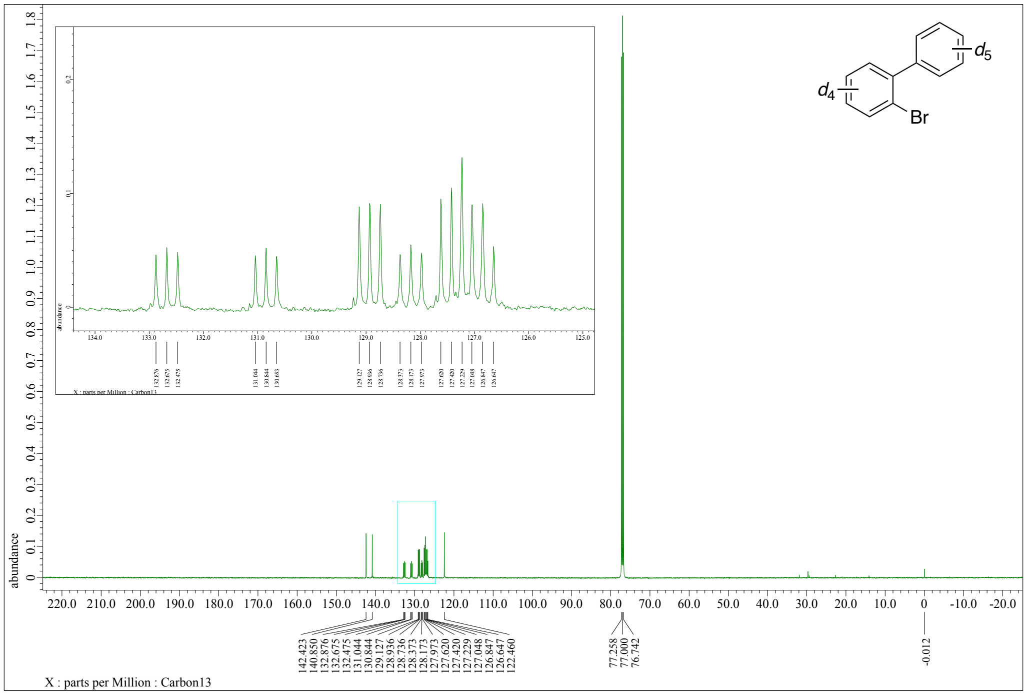

Supplementary Figure 91. ^{13}C NMR spectrum of **S1** (125 MHz, $CDCl_3$)

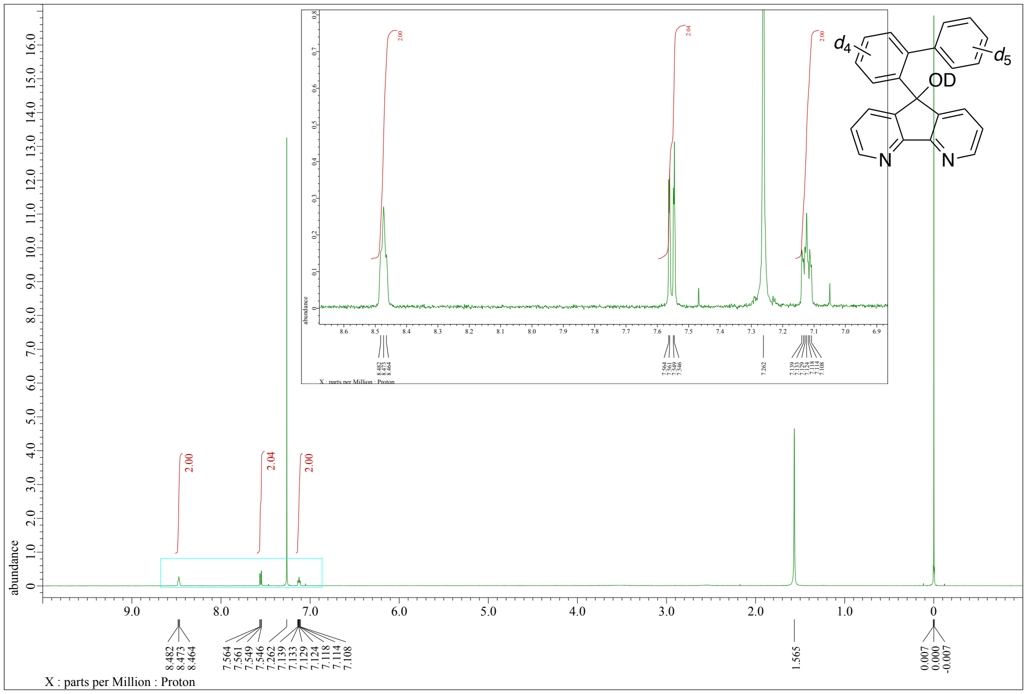

Supplementary Figure 92. ^{1}H NMR spectrum of **S2** (500 MHz, $CDCl_3$)

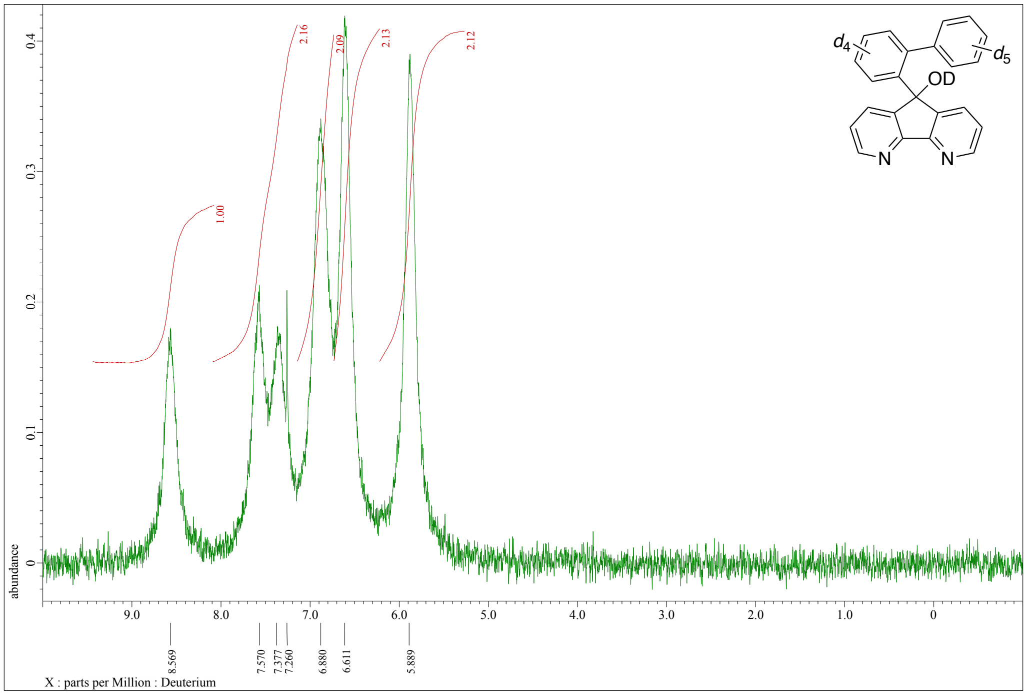

Supplementary Figure 93. ^{2}H NMR spectrum of **S2** (77 MHz, $CHCl_3$)

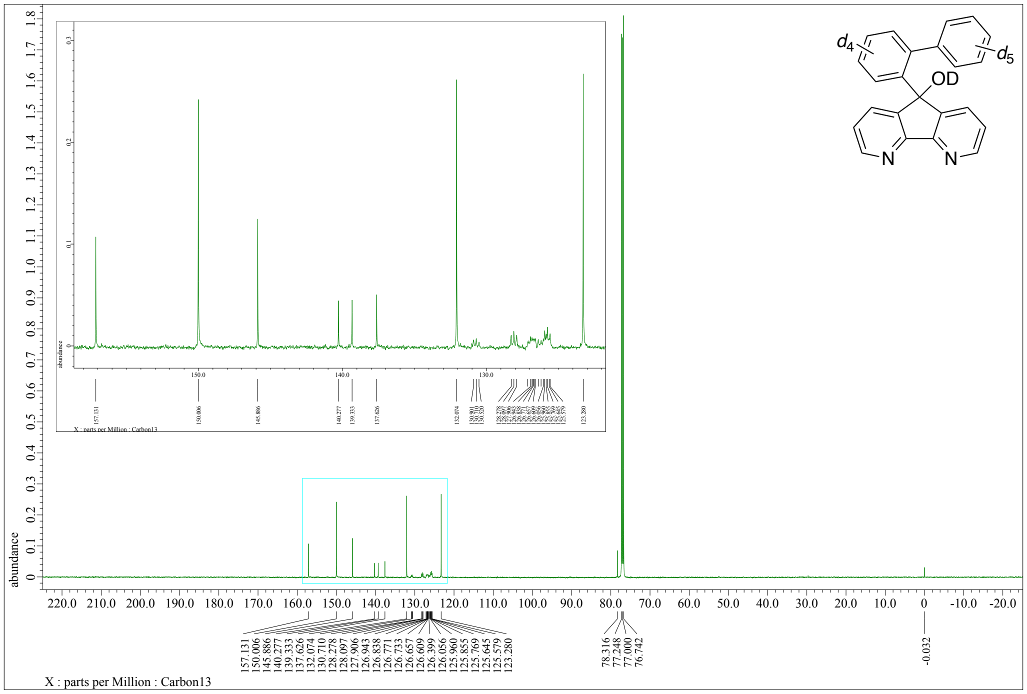

Supplementary Figure 94. ^{13}C NMR spectrum of **S2** (125 MHz, $CDCl_3$)

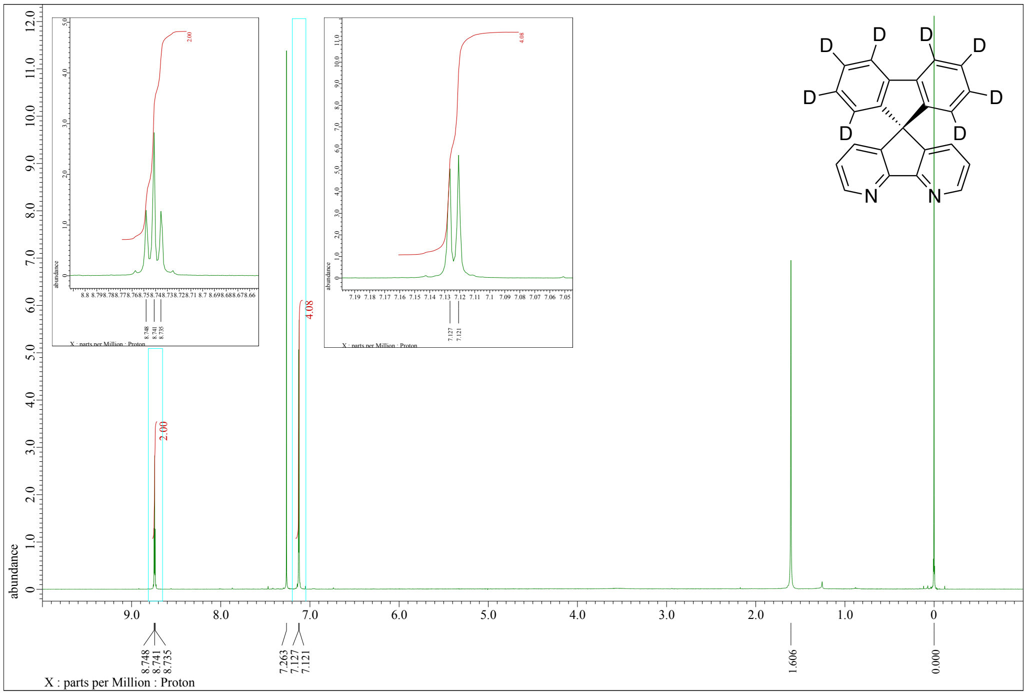

Supplementary Figure 95. ^{1}H NMR spectrum of **SpiroBpy-*d*$_8$** (500 MHz, $CDCl_3$)

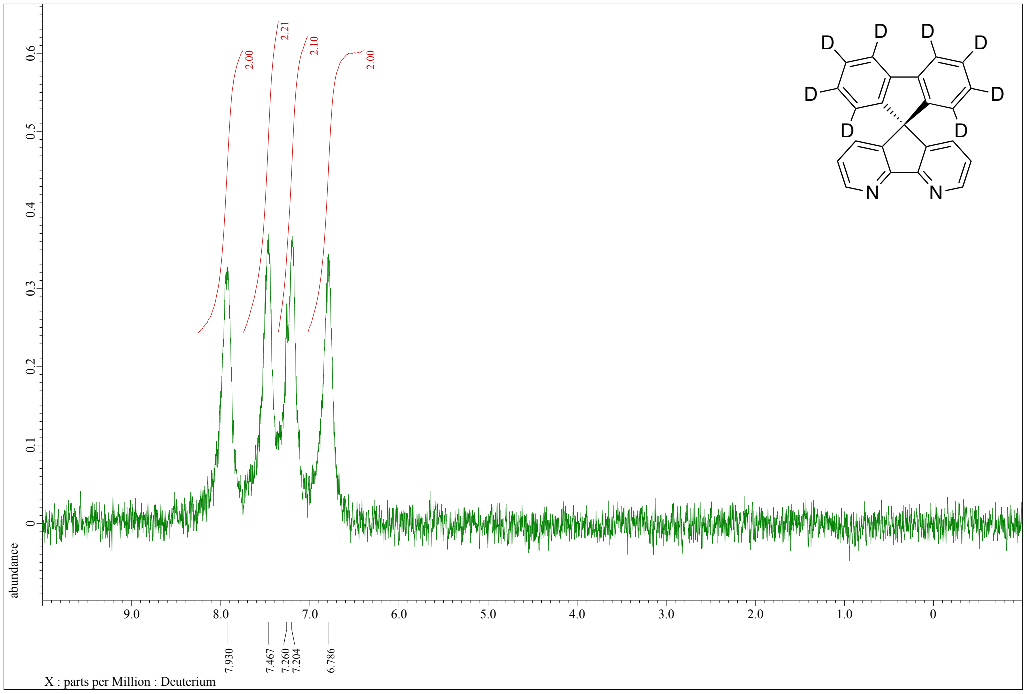

Supplementary Figure 96. ^{2}H NMR spectrum of **SpiroBpy-*d*$_8$** (77 MHz, $CHCl_3$)

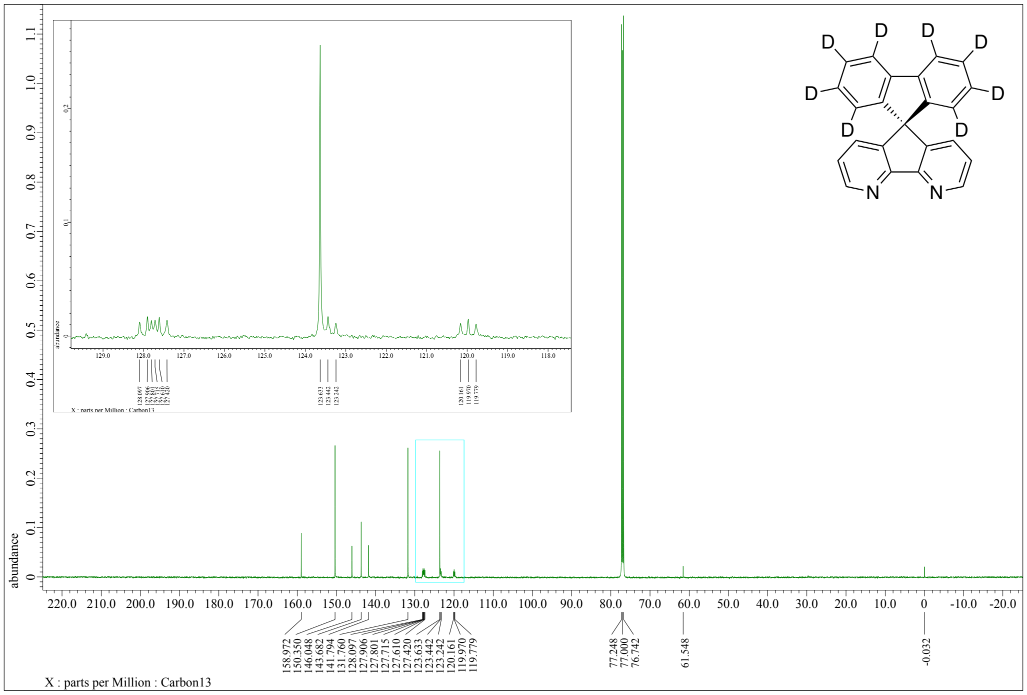

Supplementary Figure 97. ^{13}C NMR spectrum of **SpiroBpy-*d*$_8$** (125 MHz, $CDCl_3$)

II. Supplementary References

1 Still, W. C.; Kahn, M.; Mitra, A. Rapid Chromatographic Technique for Preparative Separations with Moderate Resolution. *J. Org. Chem.* **1978**, *43*, 2923–2925.

2 Pangborn, A. B.; Giardello, M. A.; Grubbs, R. H.; Rosen, R. K.; Timmers, F. J. Safe and Convenient Procedure for Solvent Purification. *Organometallics* **1996**, *15*, 1518–1520.

3 Ramadoss, B.; Jin, Y.; Asako, S.; Ilies, L. Remote Steric Control for Undirected *meta*-Selective C–H Activation of Arenes. *Science* **2022**, *375*, 658–663.

4 Chen, C.-T.; Chao, W.-S.; Liu, H.-W.; Wei, Y.; Jou, J.-H.; Kumar, S. Spirally Configured *cis*-Stilbene/Fluorene Hybrids as Ambipolar, Fluorescent Materials for Organic Light Emitting Diode Applications. *RSC Adv.* **2013**, *3*, 9381–9390.

5 Hood, J. C.; Anokhin, M. V.; Klumpp, D. A. Friedel-Crafts Reactions with *N*-Heterocyclic Alcohols. *J. Org. Chem.* **2023**, *88*, 10483–10493.

6 Liu, T.; Shao, X.; Wu, Y.; Shen, Q. Highly Selective Trifluoromethylation of 1,3-Disubstituted Arenes through Iridium-Catalyzed Arene Borylation. *Angew. Chem. Int. Ed.* **2012**, *51*, 540–543.

7 Dhankhar, J.; Gonźalez-Fernández, E.; Dong, C.-C.; Mukhopadhyay, T. K.; Linden, A.; Čorić, I. Spatial Anion Control on Palladium for Mild C–H Arylation of Arenes. *J. Am. Chem. Soc.* **2020**, *142*, 19040–19046.

8 Haubold, W.; Herdtle, J.; Gollinger, W.; Einholz, W. Darstellung von Arylhalogenboranen. *J. Organometal. Chem.* **1986**, *315*, 1–8.

9 Beller, M.; Breindl, C.; Riermeier, T. H.; Tillack, A. Synthesis of 2,3-Dihydroindoles, Indoles, and Anilines by Transition Metal-Free Amination of Aryl Chlorides. *J. Org. Chem.* **2001**, *66*, 1403–1412.

10 Liao, W.; Xu, C., Wu, X.; Liao, Q.; Xiong, Y.; Li, Z.; Tang, H. Photobleachable Cinnamoyl Dyes for Radical Visible Photoinitiators. *Dyes Pigm.* **2020**, *178*, 108350.

11 Saitoh, T.; Yoshida, S.; Ichikawa, J. Naphthalene-1,8-diylbis(diphenylmethylium) as an Organic Two-Electron Oxidant: Benzidine Synthesis via Oxidative Self-Coupling of N,N-Dialkylanilines. *J. Org. Chem.* **2006**, *71*, 6414–6419.

12 Shu, X.-Z.; Xia, X.-F.; Yang, Y.-F.; Ji, K.-G.; Liu, X.-Y.; Liang, Y.-M. Selective Functionalization of sp^3 C–H Bonds Adjacent to Nitrogen Using (Diacetoxyido)benzene (DIB). *J. Org. Chem.* **2000**, *74*, 7464–7469.

13 Wolfe, J. P.; Buchwald, S. L. Scope and Limitations of the Pd/BINAP-Catalyzed Amination of Aryl Bromides. *J. Org. Chem.* **2000**, *65*, 1144–1157

14 Li, Y.; Fang, X.; Junge, K.; Beller, M. A General Catalytic Methylation of Amines Using Carbon Dioxide. *Angew. Chem. Int. Ed.* **2013**, *52*, 9568–9571.

15 Shen, G.; Zhao, L.; Zhao, X.; Huangfu, X.; Li, Z.; Wang, R.; Zhang, T. Synthesis of Quinoxaline Derivatives via Copper(I)-Catalyzed Cross-Coupling Reaction of 1,2-Dihalobenzenes with N,N'-Disubstituted Ethane-1,2-diamines under Ligand- and Solvent-Free Conditions. *Synlett* **2017**, *28*, 1111–1115.

16 Wong, K.-T.; Chen, H.-F.; Fang, F.-C. Novel Spiro-Configurated PET Chromophores Incorporating 4,5-Diazafluorene Moiety as an Electron Acceptor. *Org. Lett.* **2006**, *8*, 3501–3504.

17 Pertegás, A. Shavaleev, N. M.; Tordera, D.; Ortí, E.; Nazeeruddin, M. K.; Bolink, H. J. Host-Guest Blue Light-Emitting Electrochemical Cells. *J. Mater. Chem. C* **2014**, *2*, 1605–1611.

18 Tredwell, M.; Preshlock, S. M.; Taylor, N. L.; Gruber, S.; Huiban, M.; Passchier, J.; Mercier, J.; Génicot, C.; Gouverneur, V. A General Copper-Mediated Nucleophilic ^{18}F Fluorination of Arenes. *Angew. Chem. Int. Ed.* **2014**, *53*, 7751–7755.

19 Amat, M.; Seffar, F.; Llor, N.; Bosch, J. Preparation and Reactions of 4-, 5-, and 6-Methoxy Substituted 3-Lithioindoles and 3-Indolylzinc Derivatives. *Synthesis* **2001**, *2*, 267–275.

20 Zhang, X.; King-Smith, E.; Renata, H. Total Synthesis of Tambromycin by Combining Chemocatalytic and Biocatalytic C–H Functionalization. *Angew. Chem. Int. Ed.* **2018**, *57*, 5037–5041.

21 Preshlock, S. M.; Ghaffari, B.; Maligres, P. E.; Krska, S. W.; Maleczka, R. E.; Smith, M. R. High-Throughput Optimization of Ir-Catalyzed C–H Borylation: A Tutorial for Practival Applications. *J. Am. Chem. Soc.* **2013**, *135*, 7572–7582.

22 Wang, G.; Xu, L.; Li, P. Double N,B-Type Bidentate Boryl Ligands Enabling a Highly Active Iridium Catalyst for C–H Borylation. *J. Am. Chem. Soc.* **2015**, *137*, 8058–8061.

23 Kim, J.; Choi, J.; Shin, K.; Chang, S. Copper-Mediated Sequential Cyanation of Aryl C–B and Arene C–H Bonds Using Ammonium Iodide and DMF. *J. Am. Chem. Soc.* **2012**, *134*, 2538–2531.

24 Manna, K.; Zhang, T.; Lin, W. Postsynthetic Metalation of Bipyridyl-Containing Metal–Organic Frameworks for Highly Efficient Catalytic Organic Transformations. *J. Am. Chem. Soc.* **2014**, *136*, 6566–6569.

25 Murai, M.; Nishinaka, N.; Enoki, T.; Takai, K. Regioselective Sequential Silylation and Borylation of Aromatic Aldimines as a Strategy for Programing Synthesis of Multifunctionalized Benzene Derivatives. *Org. Lett.* **2020**, *22*, 316–321.

26 Takaya, J.; Ito, S.; Nomoto, H.; Saito, N.; Kirai, N.; Iwasawa, N. Fluorine-Controlled C–H Borylation of Arenes Catalyzed by a PSiN-Pincer Platinum Complex. *Chem. Commun.* **2015**, *51*, 17662–17665.

27 Cooze, C. J. C.; McNutt, W.; Schoetz, M. D.; Sosunovych, B.; Grigoryan, S.; Lundgren, R. J. Diastereo-, Enantio-, and *Z*-Selective α,δ-Difunctionalization of Electron-Deficent Dienes Initiated by Rh-Catalyzed Conjugated Addition. *J. Am. Chem. Soc.* **2021**, *143*, 10770–10777.

28 Wu, X.; Han, X.; Liu, Y.; Liu, Y.; Cui, Y. Control Interlayer Stacking and Chemical Stability of Two-Dimensional Covalent Organic Frameworks via Steric Tuning. *J. Am. Chem. Soc.* **2018**, *140*, 16124–16133.

29 Meyer, F.-M.; Liras, S.; Guzman-Perez, A.; Perreault, C.; Bian, J.; James, K. Functionalization of Aromatic Amino Acids via Direct C–H Activation: Generation of Versatile Building Blocks for Accessing Novel Peptide Space. *Org. Lett.* **2010**, *12*, 3870–3873.

30 Gandeepan, P.; Cheng, C.-H. Allylic Carbon-Carbon Double Bond Directed Pd-Catalyzed Oxidative *ortho*-Olefination of Arenes. *J.Am. Chem. Soc.* **2012**, *134*, 5738–5741.

31 Morse, P. M.; Spencer, M. D.; Wilson, S. R.; Girolami, G. S. A Static Agostic α-CH•••M Interaction Observable by NMR Spectroscopy: Synthesis of the Chromium(II) Alkyl $[Cr_2(CH_2SiMe_3)_6]^{2-}$ and Its Conversion to the Unusual "Windowpane" Bis(metallacycle) Complex $[Cr(\kappa^2C,C'\text{-}CH_2SiMe_2CH_2)_2]^{2-}$. *Organometallic* **1994**, *13*, 1646–1655.

32 Gaussian 16, Revision C.01, M. J. Frisch, G. W. Trucks, H. B. Schlegel, G. E. Scuseria, M. A. Robb, J. R. Cheeseman, G. Scalmani, V. Barone, G. A. Petersson, H. Nakatsuji, X. Li, M. Caricato, A. V. Marenich, J. Bloino, B. G. Janesko, R. Gomperts, B. Mennucci, H. P. Hratchian, J. V. Ortiz, A. F. Izmaylov, J. L. Sonnenberg, D. Williams-Young, F. Ding, F. Lipparini, F. Egidi, J. Goings, B. Peng, A. Petrone, T. Henderson, D. Ranasinghe, V. G. Zakrzewski, J. Gao, N. Rega, G. Zheng, W. Liang, M. Hada, M. Ehara, K. Toyota, R. Fukuda, J. Hasegawa, M. Ishida, T. Nakajima, Y. Honda, O. Kitao, H. Nakai, T. Vreven, K. Throssell, J. A. Montgomery, Jr., J. E. Peralta, F. Ogliaro, M. J. Bearpark, J. J. Heyd, E. N. Brothers, K. N. Kudin, V. N. Staroverov, T. A. Keith, R. Kobayashi, J. Normand, K. Raghavachari, A. P. Rendell, J. C. Burant, S. S. Iyengar, J. Tomasi, M. Cossi, J. M. Millam, M. Klene, C. Adamo, R. Cammi, J. W. Ochterski, R. L. Martin, K. Morokuma, O. Farkas, J. B. Foresman, D. J. Fox, Gaussian, Inc., Wallingford CT, 2019.

33 Lu, T.; Chen, Q. Independent gradient model based on Hirshfeld partition: A new method for visual study of interactions in chemical systems. *J. Comput. Chem.* **2022**, *43*, 539-555.

34 Johnson, E. R.; Keinan, S.; Mori-Sánchez, P.; Contreras-García, J.; Cohen, A. J.; Yang, W. Revealing Noncovalent Interactions. *J. Am. Chem. Soc.* **2010**, *132*, 6498–6506.

35 Lu, T.; Chen, F. Multiwfn: A Multifunctional Wavefunction Analyzer. *J. Comput. Chem*. **2012**, *33*, 580–592.

36 Humphrey W.; Dalke A.; Schulten K. VMD: Visual Molecular Dynamics. *J. Mol. Graphics* **1996**, *14*, 33– 38.

37 NBO 7.0. Glendening, E. D.; Badenhoop, J, K.; Reed, A. E.; Carpenter, J. E.; Bohmann, J. A.; Morales, C. M.; Karafiloglou, P.; Landis, C. R.; Weinhold, F. Theoretical Chemistry Institute, University of Wisconsin, Madison (2018).
